# Climate change risks on key open marine and coastal mediterranean ecosystems

**DOI:** 10.1038/s41598-025-07858-x

**Published:** 2025-07-10

**Authors:** Abed El Rahman Hassoun, Meryem Mojtahid, Mohammad Merheb, Piero Lionello, Jean-Pierre Gattuso, Wolfgang Cramer

**Affiliations:** 1 GEOMAR Helmholtz Centre for Ocean Research Kiel, Kiel, Germany; 2 National Centre for Marine Sciences, National Council for Scientific Research in Lebanon (CNRS-L), Beirut, Lebanon; 3 Laboratoire de Planétologie et Géosciences, Univ Angers, Univ Nantes, Univ Le Mans, CNRS, LPG, UMR 6112, 2 Bd Lavoisier, 49045 Angers Cedex, France; 4 Institut Agro. INRAE, SAS, 65 Rue de Saint Brieuc, 35000 Rennes, France; 5 DiSTeBA (Department of Biological and Environmental Sciences and Technologies), University of Salento, 73100 Lecce, Italy; 6 Laboratoire d’Océanographie de Villefranche, Sorbonne Université, CNRS, 181 Chemin du Lazaret, 06230 Villefranche-sur-Mer, France; 7 Institute for Sustainable Development and International Relations, Sciences, Po, 27 rue Saint Guillaume, 75007 Paris, France; 8 Institut Méditerranéen de Biodiversité et d’Ecologie marine et continentale (IMBE), Aix Marseille Université, CNRS, IRD, Avignon Université, Technopôle Arbois-Méditerranée, Bât. Villemin – BP 80, 13545 Aix-en-Provence Cedex 04, France; 9 Université d’Avignon, 74 Rue Louis Pasteur, 84029 Avignon, France

**Keywords:** Climate change, Open marine ecosystems, Coastal ecosystems, Scenarios, Mediterranean sea, Risk assessment, Climate change, Ocean sciences, Marine biology, Biodiversity, Climate-change ecology

## Abstract

Mediterranean open marine and coastal ecosystems face multiple risks that impact their unique biodiversity, with climate change representing a major ongoing threat. While these ecosystems are also under pressure from non-climatic anthropogenic drivers (e.g., overfishing, pollution), this study primarily focuses on risks related to climate change. To assess these risks and evaluate their confidence levels, we adopt the scenario-based approach of the Intergovernmental Panel on Climate Change (IPCC), relying on a review of literature projecting changes in Mediterranean Sea ecosystems. The main drivers of environmental change are sea level rise, ocean warming and acidification. Similar to global conditions, all Mediterranean ecosystems face high risks under all climate scenarios, with coastal ecosystems being more strongly impacted than open marine ecosystems. For these coastal ecosystems, risk levels are expected to become very high already once global warming exceeds 0.8 °C with respect to the 1976–2005 period. A few Mediterranean ecosystems (e.g., coralligenous and rocky coasts) are relatively more resilient compared to others, probably because of their long evolutionary history and the presence of a variety of climatic and hydrological conditions. However, high-emission scenarios in specific sub-basins, in addition to acidification impacts, could reduce this resilience, decreasing both habitat extent and ecosystem function dramatically. Overall, due to the higher observed and projected rates of climate change in the Mediterranean, compared to global trends, for variables such as seawater temperature and pH, marine ecosystems (particularly coastal) are projected to be under higher risks compared to the global ocean.

**Supplementary Information:**

The online version contains supplementary material available at 10.1038/s41598-025-07858-x.

## Introduction

The Mediterranean Sea is one of the most important regions in the world in terms of marine biodiversity and is home to more than 17,000 marine species, almost 18% of all known marine temperate and subtropical species^1^. Among these species, ~ 20–30% are endemic^2^, making it one of the main marine biodiversity hotspots of the world. Many key resident and transient marine organisms such as fish, shellfish, and top predators (e.g., cetaceans, tuna, swordfish) are ecologically and economically important. They play crucial roles in the food web and in supporting a variety of human activities, including tourism and fisheries, which are economically important (for income and employment) in most Mediterranean countries^3^. Socio-economic and political disparities in the region are large and they influence the future development and management of Mediterranean ecosystems^4,5^.

Climate change, through changes in average as well as extremes in water temperature, pH and sea level, is one of the main drivers of risk for marine and coastal habitats worldwide^6,7^. Due to its unique geological, climatic and hydrological features^1,8^, the Mediterranean Sea is critically affected by climate change^9–12^. The projected additional impacts of climate change on marine and coastal ecosystems threaten the livelihoods of millions, as these ecosystems play a significant role in food security and coastal protection^13–15^.

One of the most important drivers of regional ecosystem change is atmospheric warming which exceeds global mean values compared to the pre-industrial period, reaching ~ 1.5 °C in 2020^16^. Regional warming will very likely continue to exceed the global mean value by 20% and may reach 5.6 °C at the end of the 21st century under a high emission scenario (RCP8.5;^17^). Warming will be particularly strong in summer, likely to exceed the global annual rates by 50%^18^. An accumulated warming of 1.3 °C has been estimated for the Mediterranean Sea surface temperature (SST) from 1982 to 2019^19^, less in the Western than the Eastern Mediterranean Basin with an increase rate that varies between + 0.29 to + 0.44 °C decade^− 1^^20–22^. For the period 1980–2020, the SST increase is more than 2-fold higher in the Mediterranean than globally (1.3 °C in the Mediterranean vs. 0.60 °C globally;^23^). The SST increase is strongest in the Eastern Basin, where some areas warmed up to + 1.2 °C in the period 2000–2017 compared to 1980–1999^16^. SST in the Mediterranean Sea is expected to increase by 0.6–1.3 °C and 2.7–3.8 °C by 2050 and 2100, under the RCP4.5 and RCP8.5 scenarios, respectively^24^. Intermediate and deep-sea temperature and salinity (below 400 m) are also significantly increasing^25–27^. By the end of the century, the projected temperature change ranges are 0.81–3.71 °C in the upper layer (0–150 m), 0.82–2.97 °C in the intermediate layer (150–600 m) and 0.15–0.18 °C in the deep layer (600 m-bottom), strongly depending on the adopted scenarios and global forcing^28^.

Marine heat waves are projected to become longer, more intense, and more frequent^21,29–32^. Although the intensity of precipitation extremes is projected to increase in some areas of the Northern Mediterranean^17,33,34^, total annual precipitation is expected to decrease over most of the basin (the average reduction rate is approximately 4% per each degree of global warming) under RCP8.5^16,18^.

Warming enhances ocean thermal stratification^35^, which may influence biogeochemical processes such as nutrient cycling and oxygen distribution. In stratified conditions, reduced vertical mixing can limit nutrient supply to surface waters and decrease oxygen replenishment at depth, potentially contributing to eutrophication and oxygen depletion in some areas. Additionally, increased dissolved organic carbon concentrations in the surface layer may play a role in these dynamics, although the mechanisms and regional expressions remain complex and context-dependent^36–38^. Increasing atmospheric CO_2_ results in acidification of both surface and deep waters^5^. Ocean acidification of Mediterranean waters (upper 80 m) occurs at rates of -0.001 to -0.009 pH units y^− 1^ depending on regions (Eastern vs. Western basin) and time period^5^. By the end of the current century, pH is expected to drop 0.28 to 0.462 pH units below the pre-industrial values depending on scenarios, with some differences between sub-basins and depths^5,39–41^. This pH decrease is ~ 1.5-fold more pronounced than the average global ocean (~ 0.3–0.4 units by the year 2100;^42^), according to most pessimistic scenarios^40,41^. Some Mediterranean sub-basins might experience more exacerbated acidification trends than the global ocean in the future^5,12,43^.

Sea level rise (SLR) has major consequences on coastal ecosystems including more frequent and/or intensive flooding along low-lying coasts, particularly in deltas and lagoons, wetlands, and some islands^1,44^, and coastal erosion^45,46^. During inundations and storm surges, SLR affects coastal infrastructures and coastal communities. Sea level has risen at a rate of about 1.2–1.3 mm yr^− 1^ since the end of the 19th century^47^ and of 1.7 mm yr^− 1^ since the mid-20th century^48^, similar to the global trend, increasing to about 2.57 mm yr^− 1^ since 1993 (based on satellite altimetry^49^), . Mediterranean sea level is projected to rise by 20 to 110 cm by the end of the 21st century compared to the 2000s^16^. Although sea level change can differ regionally due to ocean circulation and mass redistribution, projections suggest that these factors will largely balance out in the Mediterranean, leading to sea level rise rates similar to the global average^50^.

Combined impacts of climate-related habitat changes and non-climatic stressors—such as pollution, overfishing, habitat degradation, and invasive species—pose unprecedented risks to Mediterranean Sea biodiversity and ecosystem resilience, potentially pushing many species beyond the environmental conditions necessary for acclimation or adaptation. Key open marine and coastal ecosystems are already impacted, threatening their diversity, as well as the services and resources they provide^51,52^. These risks faced by the Mediterranean Sea open marine and coastal ecosystems need to be well defined to understand the implications on the health and viability of its key species. Here, we focus on climatic drivers and present an integrated overview of the main related risks that are threatening key Mediterranean open marine and coastal ecosystems. We build on the assessment performed using expert judgment conducted in the preparation of the MedECC report^1^ to evaluate the responses of key populations and ecosystems, towards various climate change risks under multiple climate change scenarios by the end of the 21st century.

## Methods

### General approach

**Fig. 1:**
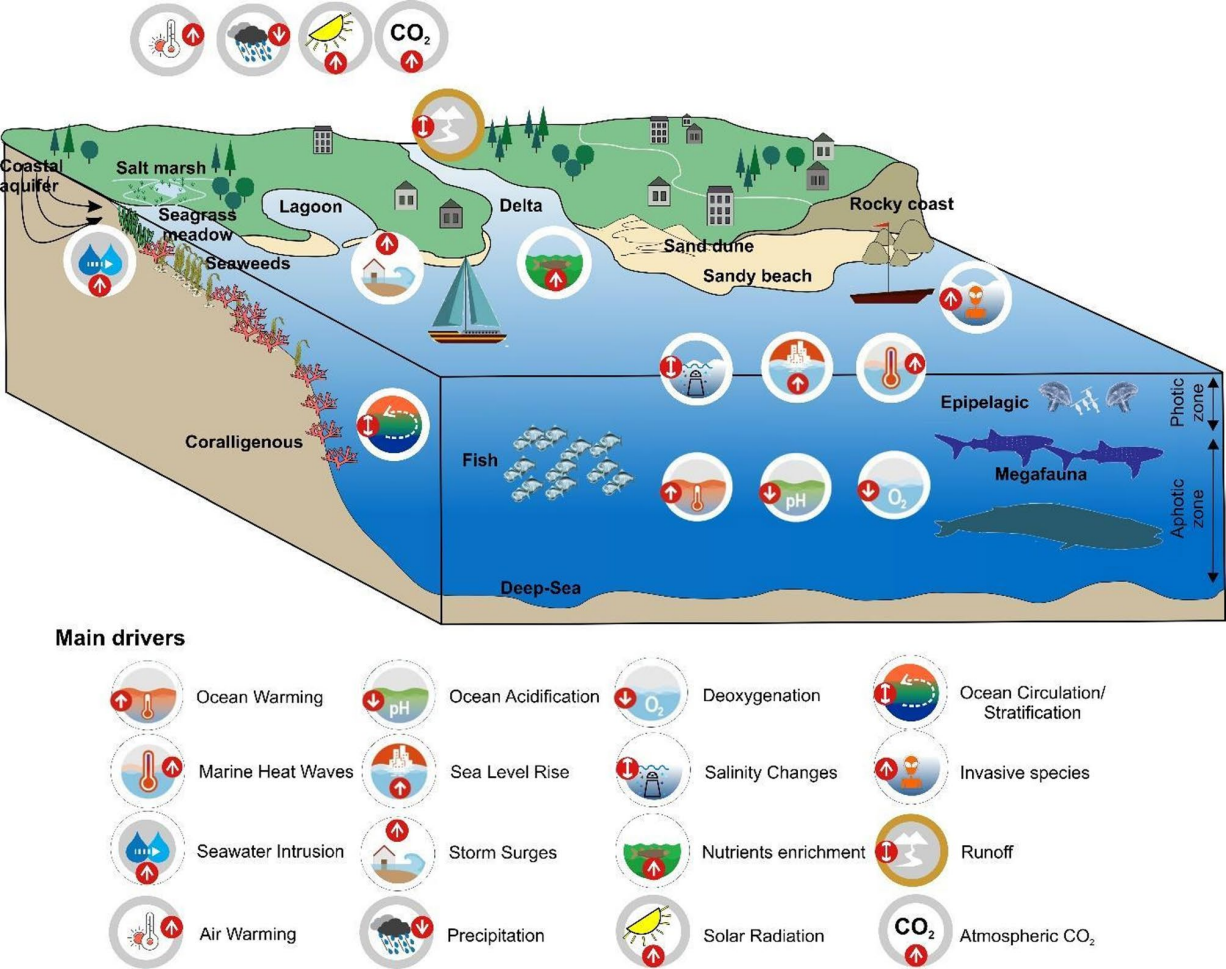
Schematic illustration of (i) the different open marine and coastal ecosystems for which we assessed risk levels, and (ii) the main drivers taken into consideration in this assessment.

This study addresses both the open Mediterranean Sea and its coastal zone. Many definitions exist to determine the spatial extent of the coastal zone. Here, we define the coastal zone as the area up to an elevation of 10 m above mean sea level (i.e. “Low-Elevation Coastal Zone” LECZ, a term used in sensitivity studies with respect to the projected sea level rise;^3,53^. The coastal ecosystems here include sandy beaches and sand dunes, rocky coasts, coastal lagoons and deltas, salt marshes and coastal aquifers (Fig. 1). The group “open marine ecosystems” comprise epipelagic ecosystems, coralligenous ecosystems, seagrass meadows, fish populations, seaweeds and megafaunal populations (Fig. 1). For simplification and within these two groups (coastal and open marine ecosystems), the term ‘ecosystems’ refers to well-defined marine and coastal systems (e.g., epipelagic zone, coralligenous formations, seagrass meadows). ‘Habitats’ denote specific biophysical conditions within ecosystems. ‘Species’ are considered at the taxonomic level, whereas ‘populations’ refer to geographically or genetically distinct groups of species. Fish were assessed as a separate group due to their ecological and socio-economic significance but are not considered ecosystems themselves. The detailed definition of each entity is provided in Supplementary Material (S1).

**Fig. 2:**
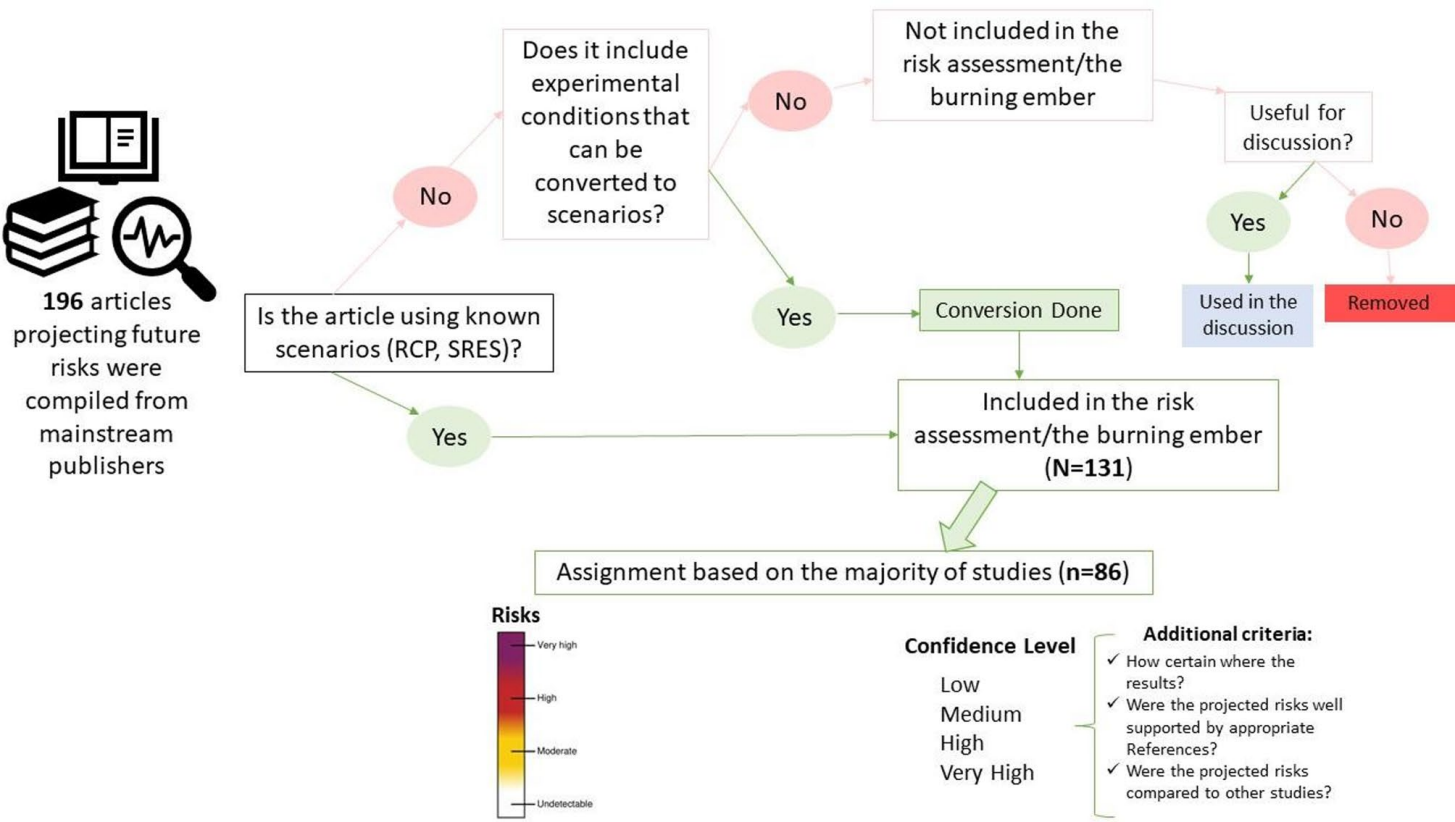
Work-flow diagram resuming the systematic approach used, from the literature assessment to the assignment of risks and confidence levels. *Literature has been compiled from various sources*, *such as Google Scholar*, *Scopus and ResearchGate.*

A literature review was conducted, searching for peer-reviewed publications that highlight projections for any of the ecosystems of interest (Fig. 2). Risk levels for biodiversity loss under various warming scenarios for six open marine ecosystems and six coastal ecosystems were developed and visualized through “burning embers”, a widely-used qualitative IPCC plot featuring risk levels (‘Undetectable’, ‘Moderate’, ‘High’, ‘Very high’). Based on the levels of evidence and agreement, a confidence level has been assigned to each projected risk^54^. Throughout the paper, the following definitions are used: **Effects** refer to observed or projected changes in species, communities, or ecosystems due to climate-related stressors (e.g., shifts in species distribution, physiological responses). **Impacts** describe broader ecological or functional consequences resulting from these effects, including changes in ecosystem structure, function, and services. Whereas, **risk** is defined following the IPCC framework, as the result of the interaction between hazard (climate stressors), exposure (species/ecosystem presence in affected areas), and vulnerability (susceptibility and adaptive capacity).

### Data compilation

Our literature review covered 196 publications (until August 2023) compiled using academic search engines (Google Scholar, Scopus and ResearchGate) to capture all available studies projecting changes in Mediterranean Sea ecosystems (Fig. 2). The searched keywords comprised the following terms: projections, forecasts, scenarios, Mediterranean Sea, with variable keywords depending on the ecosystem we were looking for (e.g., corals, fish, seaweeds, etc.). After assessing these papers, we only kept the ones that have clearly identified scenario projections (*N* = 131), the other publications were used as additional resources for discussion (Fig. 2). More specifically, we prioritized studies that provided scenario-based projections of climate change impacts on Mediterranean ecosystems, excluding those focused solely on past or present observations. The selection process emphasized predictive assessments, including modeling, laboratory experiments, mesocosm studies, and controlled in situ research, as these were essential for constructing the burning embers risk assessment. To ensure realism, we included studies that aligned with recognized climate scenarios (e.g., RCP, SSP) and IPCC methodologies, used regionally relevant temperature and pH projections, and incorporated cross-validation with in situ data. Geographic, temporal, and spatial disparities were addressed by assigning confidence levels based on study agreement and methodological robustness, with limitations explicitly discussed in the manuscript. The decision tree selection process (Fig. 2) follows a stepwise approach, prioritizing studies based on methodological robustness, emission scenario clarity, and geographic relevance. Further details on study selection criteria and confidence level assignment are provided in Supplementary Material S3.

**Fig. 3:**
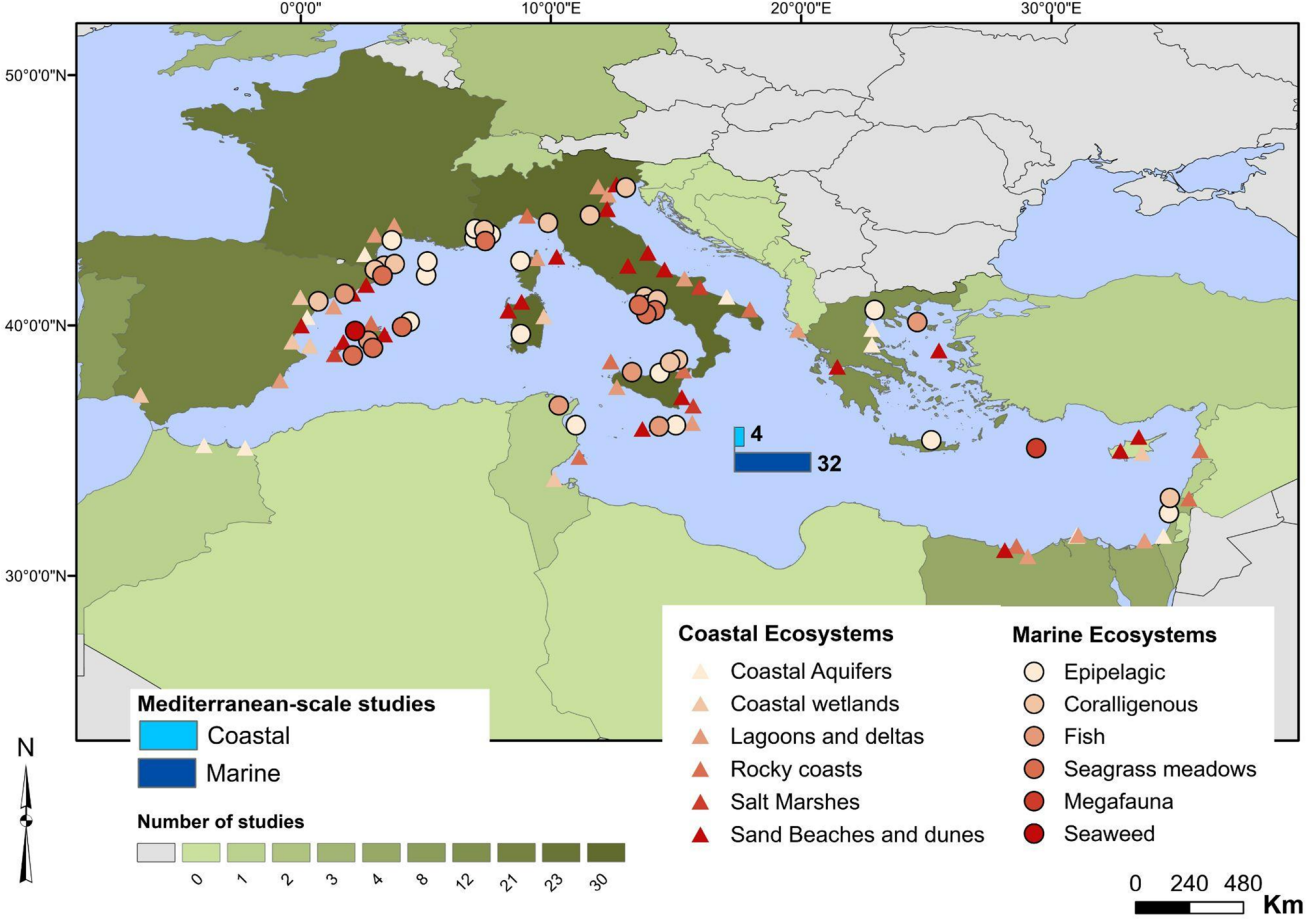
The geographic locations of the studies taken into consideration in the risk assessment (by country of the first author) and their study sites.

Data extracted from these studies comprise the emission scenarios used, the timing of the projections (mid- and/or end of the century), and other parameters taken into consideration to implement the projections (e.g., global atmospheric temperature, Mediterranean atmospheric temperature, seawater temperature, pH, SLR, etc.; see section II.3), the estimated risk(s), confidence level and the main drivers if available. Additional information was extracted from all relevant papers, such as the affiliation country of the first author, the study area, and the type of the study (i.e., modeling, laboratory experiment, mesocosm experiment, in situ study, observations near CO_2_ vents, remote sensing, review, etc.) (see Supplementary Material S2 for additional information). The locations of the study areas are shown in Fig. 3.

The publications assessed (*N* = 131) have very large geographical disparities in terms of the type of studies and study sites (Fig. 3); they also have unequal distribution across habitats. Only 11 were conducted in non-European (outside the European Union) Mediterranean countries. Italy, France, and Spain account together for 73 articles (56% of all studies). This disparity is reflected in the distribution of study sites across the Mediterranean Sea. In fact, 43 out of 50 open marine ecosystems sites and 51 out of 66 coastal sites are located in European countries. This results in a strongly biased distribution of study areas between the different Mediterranean Sub-basins, since most European countries are located in the Western realm of the Mediterranean Sea. Even for studies tackling multiple parts or the entire Mediterranean Sea (*n* = 36), 30 studies are led by researchers from Northwestern Mediterranean countries. Regional studies favor open marine ecosystems (32 out of 36). Overall, research in the Southern and Eastern Mediterranean marine and coastal areas is relatively scarce.

**Fig. 4:**
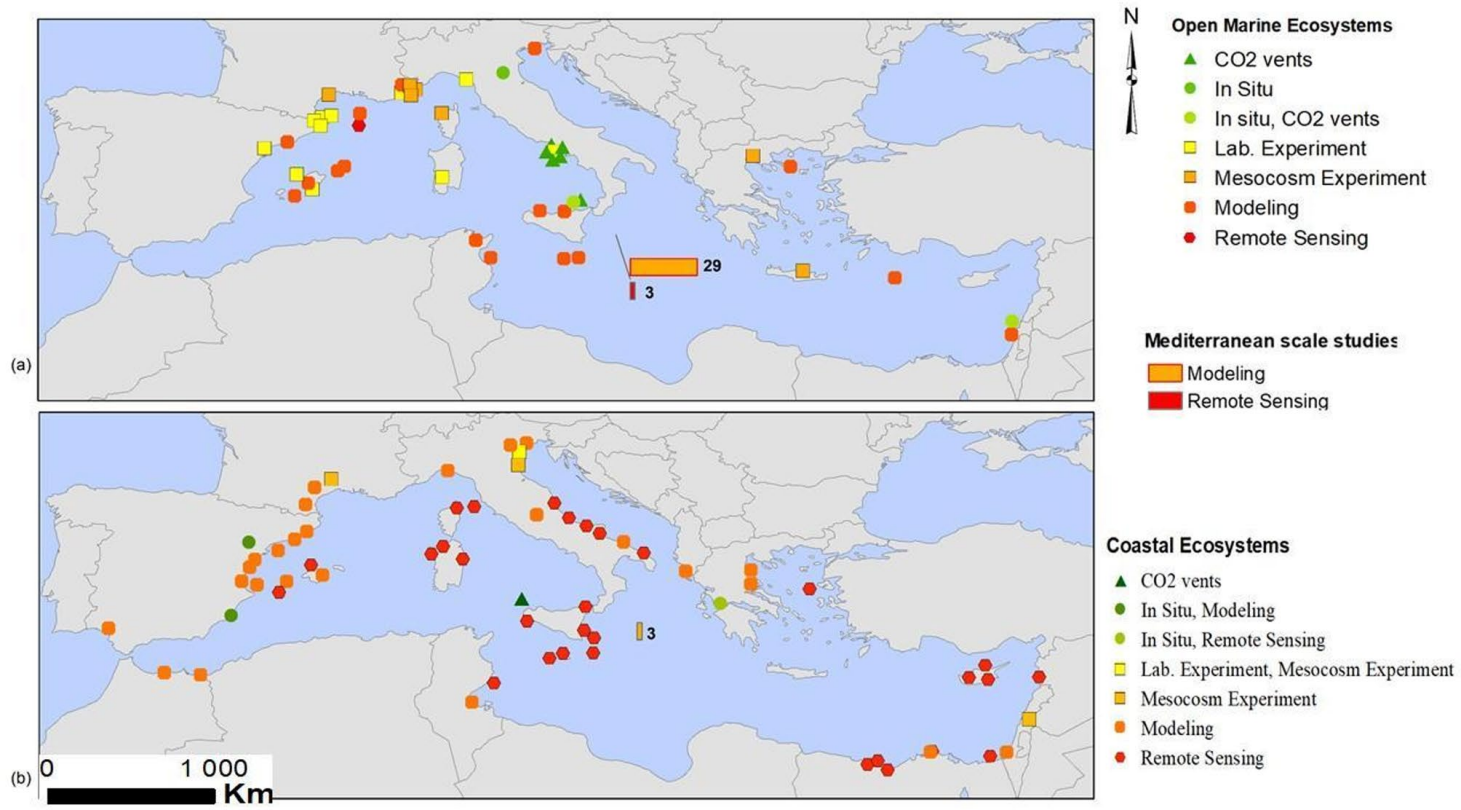
Types of the studies assessed in this paper.

Not all ecosystem types could be investigated equally. Among the 116 study sites, 66 are coastal (57%), while the remaining 50 are open marine ecosystems. The most studied open marine ecosystems are the epipelagic with 16 study sites and 11 regional studies, followed by fish populations (7 sites and 11 regional studies), and coralligenous ecosystems (17 sites and one regional study). Seagrass meadows, seaweeds, and megafauna are largely understudied with only 11, 3, and 4 study areas respectively. Only one study could be found for the deep-sea. As for coastal ecosystems, a large disparity also exists in terms of studied habitats. Sandy beaches and sand dunes are the most studied with 21 sites, followed by lagoons and deltas (15 sites and one regional study), and rocky coasts (10 sites and one regional study). Coastal aquifers, coastal wetlands and salt marshes account for 9, 8, and 5 studies respectively.

There is a disparity in the source of data used. By necessity, assessments of future conditions in most ecosystems cannot be observed – they are therefore based on well-constrained ecological model simulations. Specific local conditions in coastal ecosystems are often derived from remote sensing, while process understanding applied to the assessment is based on laboratory or in-situ experiments. Ecological model simulations are the main source for open marine ecosystems, while remote sensing is the most common for coastal ecosystems. The number of coastal ecosystem studies using modeling is also high (Fig. 4). Studies based on in situ observations are very scarce for both open marine and coastal ecosystems. Furthermore, the number of experimental studies for open marine ecosystems is relatively high (14 lab. Experiments and 7 mesocosms), there are only 4 experimental studies for coastal ecosystems. The majority of experimental studies are conducted in the Northwestern Mediterranean.

**Fig. 5:**
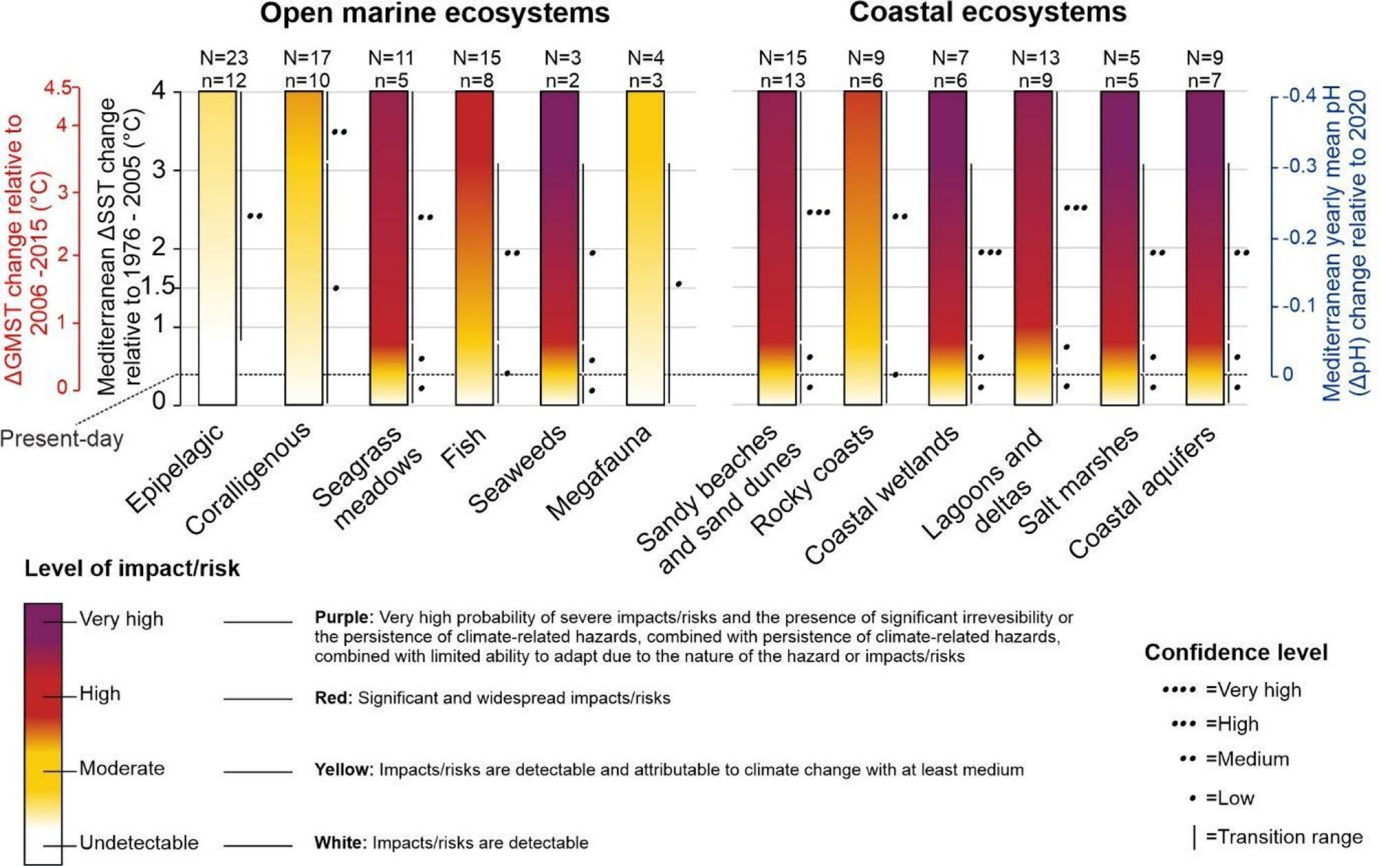
Risk assessment diagrams for open marine and coastal ecosystems in relation to observed and projected climate impacts on ecosystem structure, functioning and biodiversity. **N** is the total number of studies compiled, and **n** is the total number of studies taken into consideration in the bar. See Supplementary Materials S3 and S4 for details on the conversion of anomalies of the global mean surface temperature (ΔGMST), Mediterranean mean sea surface temperature (ΔSST) and Mediterranean pH (ΔpH) with respect to the reference and pre-industrial periods.

The detailed approach used to convert global scenarios into Mediterranean ones, to assign risks and confidence levels used to visualize the risks in Fig. 5 are all detailed in the Supplementary Material (S3). Risk levels and risk drivers with respect to the preindustrial values were also calculated, visualized and presented in S4. Finally, a comparison between risk levels in the Mediterranean vs. the ones in the global ocean are detailed in S5.

## Results and discussion

### Mediterranean key habitats undergoing change

**Fig. 6:**
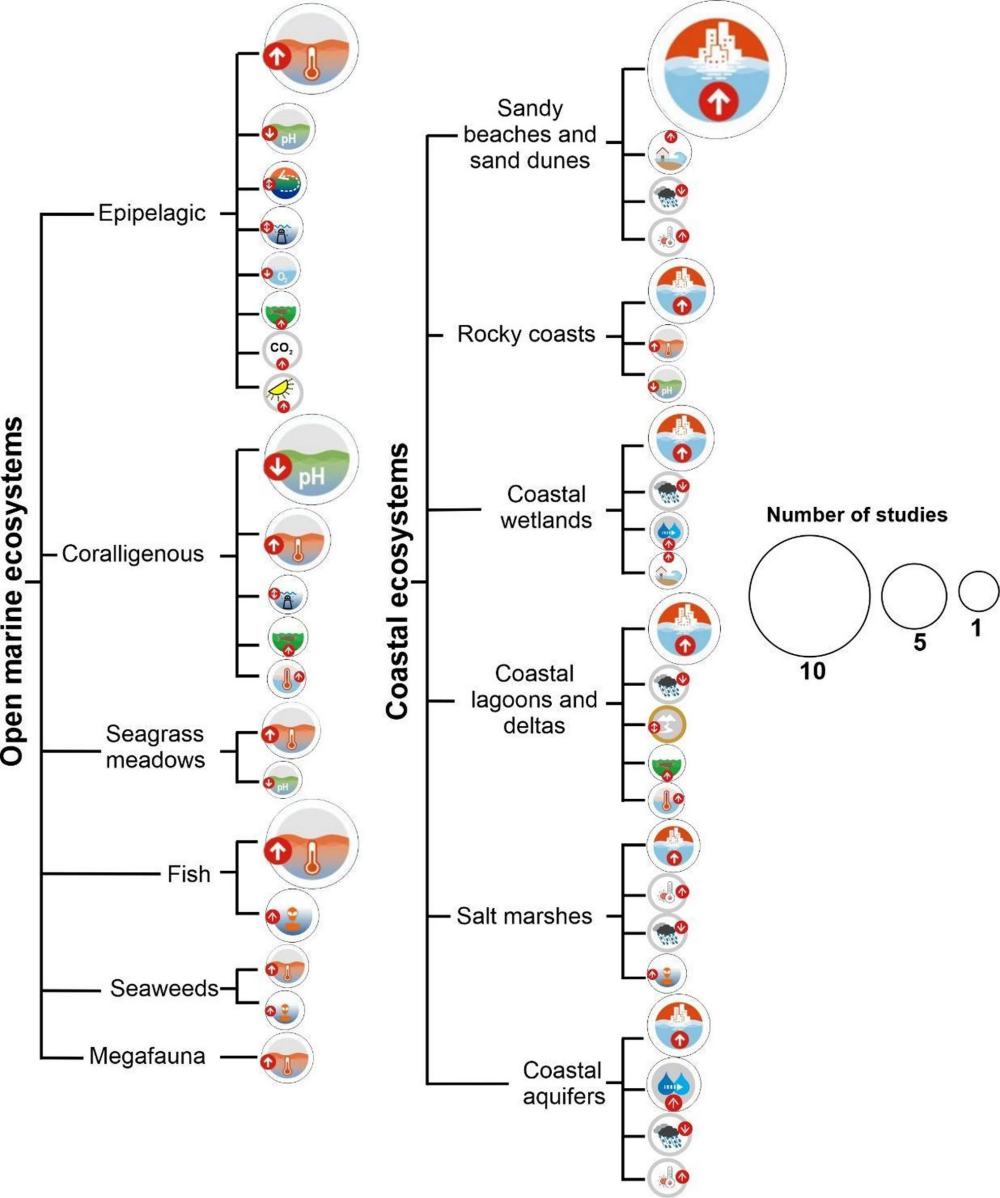
Schematic illustration of the main drivers assigned to the burning ember risk assessment for every habitat in marine and coastal ecosystems. The icons are the same as the ones used in Fig. 1. The diameter of the circle indicates the number of studies pointing out a specific driver.

Our assessment shows that severe risks on biodiversity, structure and function of coastal ecosystems are projected to be higher than for open marine ecosystems (high confidence) when Mediterranean Sea surface warming exceeds 1ºC above the reference period 1976–2005 (0.13 °C should be added to obtain the SST warming level with respect to preindustrial [see section S4]), combined with other climate-related hazards. Most coastal ecosystems assessed are projected to face an increasing risk level, from moderate to high under 2 °C ∆SST warming to high to very high above 2 °C relative to 1976–2005 (Fig. 5). The only exception is the “Rocky Coasts”, being relatively the least vulnerable. The main stressor for coastal ecosystems is linked to exposure to SLR (Fig. 6). This reflects the remarkable risks for coastal habitats posed by climate change in addition to those caused by anthropogenic pressures.

Among the open marine ecosystems, seagrass meadows and seaweeds will face the most severe risks while the least impacted will be the epipelagic (low to medium confidence level). The main stressors for the open marine ecosystems are predominantly linked to exposure to ocean warming and ocean acidification (Fig. 6).

Open marine ecosystems:

Epipelagic: Most studies highlight ocean warming as the main driver^40,41^^55–60^, with risks projected to be undetectable to moderate under + 0.8 °C <∆SST < + 6 °C (medium confidence) (*n* = 12; Fig. 5). Ocean acidification is the second most relevant driver^40,58,61^ (Fig. 5). Other less significant drivers include changes in ocean stratification/circulation^62,63^, changes in salinity^59,64^, nutrient enrichment^40^, deoxygenation^40^, atmospheric CO_2_/acidification^41^, and solar radiation^60^ (Fig. 6).

Ocean warming is expected to impact gross primary production, boosting phytoplankton exudation and bacterial growth. The planktonic community structure is generally expected to shift towards larger biomasses of small-size groups^56–58,62,65–67^, particularly pico- and nanophytoplankton, and bacteria^62^. However, several studies also highlight that the abundance of relatively large phytoplankton species (e.g., *Cyclotella* sp. and *Thalassiosira* sp.) is expected to decline due to warming, with an undetectable to moderate risk, potentially decreasing the export and energy transfer to higher trophic levels, with a very limited impact from ocean acidification/increasing *p*CO_2_^56–58,61,67^. Other studies (*n* = 11) demonstrate higher risks on epipelagic species (although with lower confidence levels). For example, ocean warming is expected to boost the expansion of Harmful Algal Blooms (HABs, e.g., *Ostreopsis ovata*) and thermophilic and/or exotic species of smaller size and of low trophic levels, which might produce biotoxins, a serious public health hazard^65,66,68–71^. Changes in species richness are also projected due to increasing temperature (predominantly during marine heat waves^72^; and changes in ocean stratification/circulation^63,73^. Additional factors are expected to change and consequently modify the epipelagic ecosystems, such as changes in nutrient concentrations (e.g., nitrite and nitrate concentrations are expected to decrease mainly due to rising temperatures and decreasing continental inputs; ^74^), weakened winter convection, surface layer warming and the changing variability of extreme meteorological events^62,75^.

In combination, these changes are expected to modify the seasonal blooms, as spring blooms may occur earlier^62,76^ and last longer^77^. Calcifiers, such as foraminifera, pteropods and coccolithophores, are among the most vulnerable organisms to combined warming and acidification effects impacting surface ocean stratification and food availability^78–80^. Epipelagic harvested species (e.g., fish, macroinvertebrate, cephalopods) are projected to significantly change in terms of stocks and distribution, mainly due to ocean warming^60,65,67,81^. Although our analysis focuses specifically on climate-related projections, it is important to acknowledge that the ecological responses of Mediterranean marine species—particularly harvested groups such as fish and invertebrates—are also shaped by long-standing non-climatic pressures, notably overfishing. Numerous studies have documented the persistent overexploitation of fish stocks across the region and its role in altering species composition, biomass, and ecosystem functioning (e.g^14,82,83^). While disentangling these effects lies outside the scope of this climate-focused synthesis, the interaction between climatic and anthropogenic drivers likely influences the magnitude and direction of projected biological changes. This reinforces the need for integrated management approaches that account for multiple, co-occurring stressors. Overall, these various contradictory findings reflect the specificities of sub-regions and drivers taken into consideration. Mediterranean basins and sub-basins will likely face non-uniform future risks, i.e., on primary production and species diversity^40,41,59,66,84^.

Coralligenous: Risks here are projected to be undetectable to moderate below ∆SST = + 3.1 °C (low confidence) and moderate to high (medium confidence) above ∆SST = + 3.1 °C (*n* = 10; Fig. 5). Most of the studies highlight acidification as the main driver^85–93^, followed by warming^86,90–93^ (Fig. 6). Other drivers were also mentioned, such as changes in salinity^93^, nutrient enrichment^93^, and marine heat waves^94^ (Fig. 6). Most coralligenous species are expected to undergo physiological or ecological changes in response to ocean warming, but current studies suggest that these changes are unlikely to lead to their complete disappearance, contrary to earlier projections^5^. At projected warming levels above + 3.1 °C, some studies suggest that ocean acidification may impact early life stages of corals such as *Astroides calycularis*^95^, while experimental and in situ studies indicate that combined effects of warming and acidification could alter rocky shore communities by reducing the presence of calcifiers such as scleractinian corals, sea urchins, and coralline algae^85^. Zooxanthellate coral species like *Cladocora caespitosa* and *Oculina patagonica*, and some cold-water coral species such as *Desmophyllum dianthus* will also face detrimental effects due to ocean acidification^87,89^. The Mediterranean red coral *Corallium rubrum*’s skeletal growth and spicule morphology could be detrimentally affected by low pH^88^. Multiple studies suggest that ocean warming and acidification may act synergistically to affect calcifying organisms in the Mediterranean. For example, warming can increase the metabolic demands of Mediterranean scleractinian corals, and when combined with lower pH, may impair cellular functions, potentially leading to mortality rates of up to 60%^91^. Similarly, for coralline algae such as *Lithophyllum cabiochae*, projections indicate that under combined warming and acidification, net dissolution may exceed calcification, increasing the risk of mortality^86^. While the physiological tolerance of some coral species to acidification has been documented^95^, calcifying algae living near their upper thermal limits are especially vulnerable and may no longer contribute effectively to reef accretion under future conditions^90^. Other studies reveal that risk levels for coralligenous reefs can be significantly higher under combined stressors and in specific sub-basin contexts, warranting a more cautious interpretation of regional projections. For example, under severe RCP 8.5 forcing, the Northern Adriatic may experience major contractions in coralligenous habitat^93^. This same study also showed that environmental variations (e.g., salinity, temperature, and nitrate concentration) under climate change conditions are expected to favor opportunistic organisms at the expense of vulnerable species in coralligenous ecosystems, potentially leading to biodiversity loss in certain regions, such as in the Northern Adriatic Sea^93^. Additionally^96–98^, demonstrated consistent patterns of reduced calcification, acidification-driven algae shifts, and cascading losses in ecosystem structure and services. These layered findings suggest that climate-driven stressors—particularly in acidified, nutrient-affected, or sub-basin-vulnerable areas—could elevate risks for coralligenous reefs beyond the mean projections.

Although the 10 studies used in our risk assessment diagram present concordant conclusions regarding the response of many coralligenous species to climate change effects, the rest of the assessed literature presents some ambiguities and contradictory results. Some coralligenous species are expected to show no physiological^99^ or mineralogical^100^ changes, even under high emission scenarios. For example, after several months of exposure to acidified conditions, the skeletal growth rate of *Dendrophyllia cornigera* showed no difference with control conditions^89^. Under low pH conditions, some species of crustose coralline algae become more resistant while others are becoming more sensitive^101^. *Ellisolandia elongata* may withstand projected temperature changes^102,103^, counteracting the effect of combined stressors (acidification and warming), although these stressors may cause shifts in the associated assemblages toward a less diverse structure, with possible dominance of the more opportunistic species^104^. Coverage of invertebrate calcifiers and crustose coralline algae appears not to be affected by the lowered pH^105^.

Although coralligenous species have a long evolutionary history in the Mediterranean, which may contribute to some degree of resilience, their persistence is contingent on environmental conditions remaining within their tolerance limits. Rising sea temperatures and increasing marine heatwave frequency are expected to drive species beyond these thresholds, placing them at high risk. Evidence from other oceanic areas shows that calcifying corals and reef habitats are already experiencing mass bleaching and mortality due to thermal stress, compounded by local stressors such as pollution and overfishing. Mediterranean corals might face similar escalating risks, underscoring the urgency of conservation efforts. The literature reveals considerable variability in species’ responses to climate-related stressors within this ecosystem, with some taxa showing tolerance or adaptability, while others exhibit signs of stress or decline^94^. This heterogeneity underscores the potential for shifts in community structure under future climate conditions.

Seagrass meadows: Risks are projected to be high to very high with ∆SST = + 0.8 °C (medium confidence) (*n* = 5; Fig. 5). Seagrass meadows are among the main Mediterranean ecological key ecosystems projected to face significant climate-related risks. Seagrass species’ responses to warming are complex, due to varying thermal performance. While some meadows exhibit thermal resilience, others suffer population declines. Under high CO_2_/low pH conditions, macroalgal communities undergo shifts with dominant species changing, while some species exhibit enhanced reproduction^106^. Ocean acidification contributes to changes in benthic communities, altering competitive dynamics between calcareous and fleshy seaweeds^107^. Projections for some seagrass species are showing generally negative results. Although negative impacts from ocean acidification on *Posidonia oceanica* epiphytic communities are projected to be smaller than previously expected^105^, *P. oceanica* might still lose 75% of suitable habitats by 2050 and is at risk of functional extinction by 2100 under high warming scenarios, as genetic diversity erosion and habitat loss are expected^108^, specifically in the Eastern Mediterranean^109^. Other studies are projecting functional extinction of *P. oceanica* by mid-century, even under relatively mild GHGs emissions^110^. Seagrass shoot mortality rates and losses are projected to increase with rising temperatures^111^, and younger life stages (i.e., seedlings of *P. oceanica*) may be particularly vulnerable^1^.

Warming in areas with excessive nutrient and organic inputs may exacerbate the risk for sediment anoxia and production of metabolites as sulfides, both detrimental for seagrass survival^110^. These results have been confirmed in a recent study^112^, projecting that *P. oceanica* meadows will experience a 70% population decline by mid-century giving way to the more resilient *Cymodocea nodosa*. Joint effects of warming and eutrophication are projected to further curtail the survival of *C. nodosa*^113^. Warming and acidification drive shifts in seagrass morphology, impacting seagrass shoot morphology and reproductive strategies and altering leaf and rhizome morphology which will affect nutrient storage, trophic interactions, and meadow resilience^114^. In addition, the joint effect of low light, increased turbidity, changes in water circulation, nutrients’ availability, and ocean warming may play a major role in the survival of *P. oceanica*, regardless of the genetic traits^115^ and the possible benefits from increased *p*CO_2_^116^. Otherwise, more suitable habitats could become available for both tropical species, *Halophila stipulacea* and *H. decipiens*, during this century under all RCP scenarios^117^. The predicted rapid expansion of these non-native species could alter the Mediterranean’s seagrass community and may have significant socio-economic consequences. Therefore, in addition to climate-driven stressors such as warming and ocean acidification, seagrass meadows are also significantly impacted by local environmental pressures, including nutrient enrichment, turbidity, habitat destruction from coastal development, dredging, and pollution from urban and agricultural runoff, all of which can exacerbate their decline.

Fish: Risks here are projected to be undetectable to moderate below ∆SST = + 0.8 °C (low confidence) and moderate to high (medium confidence) above ∆SST = + 0.8 °C (*n* = 8; Fig. 5). Most of the projection-based studies included in our assessment identify ocean warming as the primary climate-related driver of change in fish populations^65,118–124^, followed by invasive species^118,124,125^ (Fig. 6). However, other anthropogenic pressures, most notably overfishing and habitat degradation, also exert significant and compounding effects, particularly through the disruption of key fish habitats such as spawning grounds^126^. These pressures, though beyond the direct scope of this climate-focused synthesis, play a critical role in shaping fish population dynamics in the Mediterranean and should be recognized as essential co-drivers in comprehensive, integrated risk assessments. These studies are mainly predicting a significant fish stock reduction (~ 30% for RCP4.5 and ~ 40% for RCP8.5;^123^) together with a contraction of the distributional range of commercial species, with a general biogeographical displacement towards North European coasts^120^. This is in agreement with studies forecasting the shifts in suitable spawning habitats in all seasons to higher latitudes caused by warming and decreased plankton productivity affecting sardine stocks^121^. Higher temperatures are expected to boost the suitable areas for invasive species, even in protected areas, predominantly in the Eastern Mediterranean and to a lesser extent in the South Adriatic Sea and off South-West Italy^118^. For example, suitable conditions for the lionfish, *Pterois miles*, are likely to expand to the Northern and colder areas even under mild warming scenarios^124^. Ocean warming may weaken deep overturning circulation, increasing water column stratification and reducing nutrient flux to surface waters. This is projected to lower primary production, with potential additive impacts on sardines due to food scarcity and synergistic effects on anchovy and mackerel, whose responses also depend on thermal tolerance and reproductive timing^123^. More severe risks are projected by other studies (*n* = 3), predicting a significant loss of climatically suitable habitats for endemic species^127^ with climate-related local extinctions of the most harvested small pelagic species in Europe, mainly in the South-Eastern Mediterranean^81^, and a considerable expansion of *Pterois miles* towards new areas^125^. In contrast, other studies (*n* = 4) indicate only undetectable to moderate risks. These studies mostly predict an increase of suitable areas/gains (e.g., for anchovy spawning habitats), total biomass, total length at catch, total catch with some spatial and inter-species contracts with increases mainly projected in the Eastern Mediterranean and the Iberian Peninsula^60,65,121,128^ in parallel with potential distribution shifts northward (e.g., round sardinella;^128^).

Seaweeds: Risks here are projected to be undetectable to high below ∆SST = + 0.8 °C (low confidence) and high to very high (low confidence) above ∆SST = + 0.8 °C (*n* = 2; Fig. 5). These studies identify ocean warming^129,130^, followed by invasive species^129^ as main drivers (Fig. 6). The main risks include diversity loss (e.g., of *Cystoseira* macroalgae) due to habitat retractions and genetic erosion, mostly in the Eastern Mediterranean Sea^130^. This loss could have cascading effects on the whole ecosystem and its services^131,132^. Projections include triggering high abundance of invasive seaweeds in coastal areas (e.g., *Acrothamnion preissii*, *Lophocladoa lallemandii* and *Caulerpa cylindracea*), accelerating the decline of already threatened native habitats, such as seagrasses^111^ and gorgonians^133^. This process can be attributed to the reduction in biotic resistance of native communities to the arrival of non-indigenous seaweeds^129^. Another study, not taken into consideration in our risk assessment as it has low confidence level, reflects more complex projections showing that climate-induced range shifts may be less drastic and thus most species are unlikely to completely disappear (e.g., *Padina pavonica*,* Halopteris scoparia*;^103^). These results suggest marked differences in warming sensitivity within and between benthic communities^134^.

Megafauna: Risks are projected to be undetectable to moderate below ∆SST = + 3.1 °C (low confidence) (*n* = 3; Fig. 5). These studies are overwhelmingly stating ocean warming as the main driver^135–137^ (Fig. 6). The main risks include a disproportionate loss of functional diversity^137^, an increase in the daily energy expenditure and thus an alteration of the physiological functions of marine turtles^135^ with contractions of their foraging space^136^. There is low confidence in the identification of these risks as foraging areas are likely to increase by up to 10%, mainly in neritic zones^136^. Although ocean warming is identified as the primary climate-driven risk to megafauna, these impacts are largely indirect, occurring through habitat modification, shifts in prey distribution and abundance, and altered environmental cues affecting migratory species (e.g., cetaceans and turtles). The scarcity of studies on Mediterranean megafauna, particularly for cetaceans, limits our understanding of climate-related effects, which are often confounded by other stressors such as pollution, overfishing, vessel strikes, and habitat degradation. These non-climatic factors may exert a stronger or more immediate influence on population dynamics and species distributions, highlighting the need for integrative assessments of cumulative risks. Overall, megafauna-related projections are very limited in the Mediterranean, highlighting a notable research gap and emphasizing the need for further studies on how climate-driven changes may affect their distribution and ecological roles (e.g. for sharks and other megafaunal species). Already observed changes include a poleward shift and an alteration of the migration timing for some cetaceans^138^. While expected risks encompass megafaunal range shifts for some species, such as the westward expansion of loggerhead turtles within the Mediterranean^139^, others face the risk of local population collapse, as is the case of the critically endangered common dolphins *Delphinus delphis* in the Gulf of Corinth^140^. 21–31% of Mediterranean marine ecoregion species have high climate risk^11^, increasing the risk of extinction of critical species even in protected areas. While fin whales can leave the Western Mediterranean Sea through the Strait of Gibraltar, the 12 Hellenic Trench cetacean species^141^, primarily deep-diving cetaceans such as sperm whales and Cuvier’s beaked whales that inhabit the deep waters off southwestern Greece, are surrounded by much shallower seas that make it difficult to leave^142^. In addition, Mediterranean-wide shifts in prey distribution and abundance driven by climate change and anthropogenic disturbance are expected for the black anglerfish^143^, which we include here due to its ecological role as a large benthic predator frequently assessed in climate-related deep-sea ecosystem studies—although it does not fit traditional definitions of marine megafauna.

### Coastal ecosystems

Sandy beaches and sand dunes: Risks are projected to be undetectable to high below ∆SST = + 0.8 °C (low confidence) and high to very high (high confidence) above ∆SST = + 0.8 °C (Fig. 5). SLR is by far the dominant driver (Fig. 6)^144–155^. Other drivers include storm surges^146,155^, changing precipitation and warming^156^ (Fig. 6). The major risks of SLR are shoreline retreat and coastal inundation. Modeling results project very severe erosion and floodings from as early as mid-century particularly under the combined effects of the projected mean SLR and storm surges^144,145,147^^149^–^152,154,155^. Due to their low elevation and proximity to the sea, sandy beaches are at higher risk^146,153,157,158^ compared to dunes^147^. The transition dune habitat is projected to remain stable, although mobile and fixed dune habitats are projected to lose most of their actual distribution, the latter being more sensitive to climate change effects. The partial or total destruction of sandy beaches and dune habitats seriously threatens species and biodiversity hampering these ecosystems’ resilience^159^. For example, a SLR of 1.2 m is expected to cause a loss of 67.3% and 59.1% for loggerhead and green turtle nesting sites respectively^148^. The specificity of sandy beaches as narrow ecotones between sea and land may be lost, adversely affecting fine-tuned macrofaunal adaptations and therefore ecosystem functioning^159^.

Rocky Coasts: Risks are projected to be undetectable to moderate below ∆SST = + 0.8 °C (low confidence) and moderate to high (medium confidence) above ∆SST = + 0.8 °C (Fig. 5). Among climate-related stressors, SLR is identified as the major driver (Fig. 6)^149,152^^160–162^, with additional risks from ocean warming^161^ and ocean acidification^163^ (Fig. 6).

Compared to other coastal ecosystems, SLR-related risks appear lower for rocky shores at ∆SST above + 0.8 °C, primarily because their elevation provides some degree of protection^149,152^. However, localized impacts may be significant. For instance, certain rocky shore habitats in North-Eastern Sicily are projected to experience gravity collapse events due to SLR^162^. These physical changes will directly affect resident populations and biodiversity, as submerged horizontal rocky surfaces tend to host lower species richness compared to intertidal reef platforms^160^.

In intertidal rocky shore communities, biodiversity shifts are expected. When permanently submerged, these communities may transition into either a structurally different but still rich assemblage (when protected from grazing) or a much poorer turf-dominated community (when exposed to grazers), leading to a drastic reduction in reef community net production^160^. Additionally, insects inhabiting splash pools, such as the culicid *Acartomyia mariae*, are vulnerable to both SLR and warming due to their strong dependence on temperature and salinity^161^. While *A. mariae* itself is not known to pose direct sanitary risks, changes in splash pool habitats could indirectly influence the distribution and emergence of other mosquito species relevant to human health, with implications for coastal ecosystem management.

Ocean acidification will also contribute to the degradation of specific rocky shore habitats, particularly vermetid reefs. The recruitment of these reefs, especially of pH-sensitive gastropods, is expected to decline^163^. Without reductions in CO₂ emissions and active conservation efforts, these reefs face a high risk of extinction. Similarly, patellids are projected to undergo significant range shifts by 2050 and 2100, with southern populations declining and northern populations expanding^164^.

Overall, the impacts on rocky shores are highly habitat-dependent, with SLR driving physical changes, ocean warming influencing species distributions and community structure, and acidification threatening key ecosystem engineers like vermetid gastropods. These risks, coupled with seasonal and successional dynamics, emphasize the need for targeted conservation strategies for different rocky shore habitats.

Coastal wetlands: Risks to coastal wetlands are projected to be high to very high above ∆SST = + 0.8 °C (high confidence) (*n* = 6; Fig. 5). These risks are primarily driven by sea level rise (SLR)^146,149,165–167^ (Fig. 6), with additional contributions from changing precipitation patterns^168,169^, seawater intrusion^166^, and storm surges^146^ (Fig. 6). In this assessment, coastal wetlands refer specifically to intertidal and supratidal wetlands, including coastal freshwater and brackish wetlands, while excluding coastal lagoons, deltas, salt marshes, and coastal aquifers, which are analyzed separately. These wetlands are highly dynamic and sensitive to climate-induced hydrological shifts, with SLR and salinity intrusion expected to significantly alter wetland structure, water balance, and habitat stability.

Projected increases in salinity and sulfide concentrations may induce physiological stress in wetland biota, leading to community shifts and disruptions in ecosystem function^170^. In particular, altered hydrological conditions can impact crustacean and zooplankton communities hatching from resting egg banks, reducing the establishment of large branchiopods and copepods. This could shift wetland ecosystems from a zooplankton-rich clear-water state to a zooplankton-poor turbid state, fundamentally altering invertebrate diversity and food web dynamics^171^.

Beyond direct habitat degradation, cascading ecological consequences are projected for wetland-dependent species. Waterbird populations that rely on these wetlands for breeding and residency may experience habitat loss and declining environmental suitability^168^. However, some migratory and wintering species, particularly small wading birds, may benefit from habitat shifts, as muddy areas and open water expand under increased salinity, enhancing foraging conditions^168^.

Overall, climate-driven hydrological shifts, salinity changes, and extreme weather events are expected to fundamentally reshape coastal wetland structure and function, with significant implications for biodiversity, habitat stability, and ecosystem resilience. Without targeted conservation and adaptation efforts, these habitats will face increasing degradation and loss in the coming decades.

Coastal lagoons and deltas: Risks here are projected to be high to very high starting from a ∆SST = + 1.0 °C (high confidence) (*n* = 9; Fig. 5). SLR is by far the major driver affecting these ecosystems^149,153,172–174^ (Fig. 5). Other drivers include changing precipitation^175^, terrestrial runoff^175^, nutrient enrichment^176^, and marine heat waves^72^ (Fig. 6). The predicted impacts include a remarkable increase of SLR (e.g., up to 160% in 2100 in the Northern Adriatic;^174^), causing floodings^173^ and loss of important habitats nesting beaches, i.e., for the loggerhead (*Caretta caretta*), such as in the Egyptian coasts/Nile delta region^172^. These effects are expected to have significant environmental and socioeconomic consequences, as many lagoons and deltas will be highly vulnerable^153^. In addition, risks include drier soil moisture conditions, negative effects on water quality comprising anoxic crises, intensified terrestrial storm runoff, providing coastal ecosystems with large nutrient pulses and increased turbidity, with unknown consequences for the phytoplankton community^175^. These latter are expected to witness an altered natural succession due to heat waves, as cyanobacteria and chlorophytes are favored at the expense of haptophytes^72^. Also, it is predicted that *Caulerpa prolifera*, that is significantly uptaking nutrients avoiding thus the occurrence of high phytoplankton densities, will be negatively affected, worsening eutrophication^176^. Otherwise, high to very high risks with low confidence level include more coastal hazards, causing significant loss of coastal lands^149^ and continuous shoreline erosion^177^. Undetectable to moderate risks attributed to SLR are also expected in specific areas such as the Ebro Delta^178^ with relatively higher rates in the Eastern Mediterranean Sea (e.g., Egypt) compared to its Western part^157^. Moderate risks are forecasted for macroinvertebrates in coastal lagoons due to ocean acidification^179^. Other studies are expecting less drastic effects on the zooplankton community, with even positive influence, although their structure will be subjected to changing competitive interactions^180^. Other studies (not included in our analysis as they do not present projections) show site-specific impact combinations^181^. In addition to the increasing vulnerability of coastal areas due to SLR^182^, shifts in freshwater and nutrient runoff are projected to drive phytoplankton communities toward more extreme “famine or feast” dynamics—characterized by sudden blooms followed by periods of scarcity—altering ecosystem functioning in shallow coastal lagoons^183^. Human activities are expected to worsen the risks due to natural habitat destruction and alteration of the hydrological cycle (e.g^184^).

Salt marshes: Risks are projected to be high to very high above ∆SST = + 0.8 °C (medium confidence) (*n* = 5; Fig. 5), with SLR as the main driver^149,185^ (Fig. 5). Other drivers also include atmospheric warming, precipitation change^186^, and invasive species^187^ (Fig. 6). The risk of SLR was mostly assessed along the Italian coasts (Sicily and coastal areas of the Adriatic Sea;^149,150,185^). Projections show important flooding in mid- and end of the century resulting in habitat submersion. This change is expected to be accelerated by natural and anthropogenic land subsidence in some areas^150,185^. Risk maps for other low-lying areas, involving several islands (Sardinia, Elba Island in Italy; Corsica in France; Cyprus; Kerkennah in Tunisia; Majorca and Ibiza in Spain), provide an estimated potential land loss of about 150 km^2^ for the RCP8.5^149^. The physical destruction of such habitats, together with climate-related drivers such as air warming and droughts, is already altering the structure and function of Mediterranean salt marshes. In the North Adriatic Sea, for instance, the combined effects of inundation, rising temperatures, and reduced precipitation are leading to rapid vegetation shifts from perennial grasses to annual succulents^186^ - a potential early warning sign of ecosystem deterioration^188^. Additionally, the Mediterranean will potentially experience a sharp drop in the richness of *Spartina* spp. With higher potential for invader species of *Spartina* spp. (e.g., *S. anglica*) to expand northward^187^.

Coastal aquifers: Risks here are projected to be high to very high starting from a ∆SST = + 0.8 °C (medium confidence) (*n* = 7; Fig. 5). It is noteworthy that the same number of studies (*n* = 7) found moderate to high risk, but with low confidence level, which is why we assigned the first category to this ecosystem. The studies considered in this risk assessment (high to very high risk) clearly show that SLR is the main driver^189–193^ (Fig. 6). Other drivers include seawater intrusion^191,193,194^, decrease in precipitations^195^, and air warming^196^ (Fig. 5). The main projected risks include decreasing trends in groundwater levels, mostly in the recharge zone^196^ with growing effects of seawater intrusion^191,193,194^, considerable changes in flow velocity, the drainage of the aquifer upstream areas^189^, and losses in groundwater resources^190^. In addition, the decrease in precipitations is expected to increase groundwater consumption, exacerbating the withdrawal trend^192^. Other studies project moderate to high risks (*n* = 7), with a groundwater recharge decreasing trend as a response to changes in precipitation^197^. This variability in groundwater recharge posed by the high variability of precipitation will increase the aquifer’s deterioration potential of both its quantity and quality status, and clearly stating that seawater intrusion might have stronger impacts compared to SLR^198^. While not included in the formal risk assessment due to the absence of climate projections, other studies provide useful context, showing that extensive areas in coastal zones (e.g., the Nile Delta) may become submerged, with coastlines shifting inland by several kilometers^199^. Such changes are associated with salinization effects that negatively influence survival and reproduction in soil invertebrates^200,201^, and trigger avoidance behavior in species like earthworms^202^.

#### Effects of the methodological limitations on the risk assessment

Our methodological approach has certain limitations that should be acknowledged. First, the geographic and temporal disparities in available studies introduce biases, as most projections originate from the northern Mediterranean, with limited data from the southern and eastern sub-basins. This imbalance affects the regional representativeness of our findings. Second, our reliance on scenario-based projections means that studies focused solely on past or present observations were excluded from the risk assessment, potentially overlooking important baseline trends. Third, while we aimed to integrate multi-stressor interactions, most available studies assess individual climate drivers rather than their combined effects, limiting our ability to quantify synergistic or antagonistic responses. Additionally, uncertainties inherent in modeling, laboratory experiments, and mesocosm studies—such as variations in experimental duration, scale, and environmental representativeness—may influence projected impacts. To mitigate these limitations, we assigned confidence levels based on study agreement and methodological rigor, but we recognize the need for further research to improve the granularity and comprehensiveness of climate risk assessments in the Mediterranean.

## Conclusions

In order to determine the projected future risks in the Mediterranean Sea and its marine and coastal ecosystems, we conducted a systematic risk assessment based on the available literature, following the IPCC methodology. Our synthesis reveals a diversity of responses among key Mediterranean habitats and ecosystems to various climate-related hazards across different warming scenarios. When the increase in sea surface temperature (∆SST) exceeds 0.8 °C relative to the 1976–2005 baseline, risks are projected to be high to very high (low to medium confidence) for seagrass meadows and seaweeds, and moderate to high for fish populations. Epipelagic ecosystems are projected to be more resilient, with risks ranging from undetectable to moderate. For coralligenous assemblages, moderate to high risks are projected when ∆SST surpasses 3.1 °C (medium confidence). Additionally, our assessment shows that all examined coastal ecosystems are likely to experience high to very high risks beyond the 0.8 °C threshold (medium to high confidence), except for rocky shores, which appear more resilient, with a slower risk escalation from moderate to high (medium confidence). These results reflect patterns consistently reported across the literature and underscore the urgency of addressing climate-driven risks in the region’s most sensitive habitats.

Similar to global trends, all Mediterranean coastal ecosystems are projected to face higher risk levels than open marine ecosystems, partly due to their exposure to multiple anthropogenic stressors, including coastal development, pollution, and resource overexploitation, which intensify the impacts of climate-driven changes. The remarkably higher projected vulnerability of these ecosystems might be related to the rates of climate change in the Mediterranean that exceed global trends for most variables. However, climate change-related stressors seem to impact marine and coastal ecosystems differently than in the global ocean, as epipelagic and coralligenous ecosystems are expected to be more resilient while seagrass meadows and seaweed are predicted to have higher risks.

Our assessment also highlights a remarkable gap in studies projecting the future response of key ecosystems and biological groups such as deep-sea habitats, megafaunal populations, seaweeds, and salt marshes. Additionally, the lack of comprehensive baseline data and long-term observational studies significantly constrains the reliability and interpretation of these projections. Addressing these foundational gaps is essential to better understand current ecosystem conditions, assess changes over time, and improve the accuracy of future risk assessments. Furthermore, our findings underscore a significant geographical imbalance, with most studies being Euro-centric and predominantly focused on the Northern and Western sub-basins of the Mediterranean Sea.

This study ultimately highlights the main risks projected for key open marine and coastal ecosystems in the short- and long-term, under various climate change scenarios, and can be used as a baseline to guide researchers on gaps and areas where the uncertainty is high and need to be urgently addressed, and policymakers on the ecosystems that need urgent measures to efficiently improve their resilience.

## Directions for future research and applications

To enhance the scientific understanding and management of climate change risks on Mediterranean marine and coastal ecosystems, we propose the following directions for future research and applications. These recommendations address key gaps identified during this review process and aim to guide effective policymaking and adaptive management strategies. By addressing the highlighted gaps, future studies can significantly contribute to more accurate risk assessments, effective management practices, and informed policymaking.

1. Increased Granularity and Regional Specificity.

The Mediterranean Sea’s ecological diversity necessitates region-specific analyses to account for its heterogeneous sub-basins. Future research should **conduct** detailed, sub-regional assessments to capture the unique oceanographic and ecological dynamics of the Western, Central, and Eastern Mediterranean basins, **utilize** high-resolution climate models to provide localized projections of environmental stressors, such as sea temperature rise, marine heatwaves, and ocean acidification, and **investigate** the species-specific responses within different habitats to improve predictions of ecosystem resilience and vulnerability.

2. Methodological Enhancements in Risk Assessment

To strengthen the robustness and transparency of risk assessments, future studies should **implement** standardized systematic review protocols with clear inclusion and exclusion criteria to ensure consistency and reproducibility, **expand** the use of the IPCC risk framework, explicitly defining and quantifying risk as a function of hazard, exposure, and vulnerability, and **integrate** cross-validation techniques that compare model projections with in-situ observations, mesocosm experiments, and historical trend analyses.

3. Addressing Uncertainties and Multi-Stressor Interactions

A comprehensive understanding of climate risks requires rigorous examination of uncertainties and stressor interactions. Thus, we recommend **quantifying** uncertainties associated with different methodologies, including modeling projections, laboratory experiments, and mesocosm studies, **investigating** synergistic and antagonistic interactions among multiple climate-related stressors, such as warming, acidification, deoxygenation, and sea level rise, and **utilizing** advanced ecosystem modeling approaches that simulate complex multi-stressor scenarios, thereby enhancing predictive accuracy.

4. Comprehensive Literature Integration and Gap Analysis

To address geographic and thematic disparities in existing research, we suggest **expanding** research efforts in underrepresented regions, particularly in the Eastern and Southern Mediterranean sub-basins, **promoting** cross-disciplinary collaboration to incorporate socio-economic, cultural, and governance dimensions into climate risk assessments, and **developing** comprehensive meta-analyses that synthesize findings across disparate studies to provide unified risk narratives.

5. Policy and Management Implications

To bridge the gap between science and policy, future applications should **develop** region-specific adaptation strategies that account for the socio-economic contexts of vulnerable communities, **implement** ecosystem-based management (EBM) frameworks to address the cumulative impacts of climate change and non-climatic stressors, such as pollution and overfishing, **enhance** transboundary cooperation among Mediterranean countries to support coordinated conservation efforts and marine spatial planning.

6. Recommendations for Future Research Directions.

To advance the field and support evidence-based decision-making, future research should focus on:

- Multi-stressor experimental studies that quantify cumulative impacts on species interactions, biodiversity, and ecosystem functions.
- Spatially explicit vulnerability mapping to identify high-risk areas and inform targeted conservation strategies.
- Integrative modeling approaches that link ecological risks with socio-economic impacts, enhancing the relevance of scientific findings for policy development.

## Electronic supplementary material

Below is the link to the electronic supplementary material.

Supplementary Material 1

Supplementary Material 2

Supplementary Material 3

## Data Availability

All data generated or analyzed during this study are included in this published article [and its supplementary information files].

## References

[1] Balzan, M. V. et al. Ecosystems. In Climate and Environmental Change in the Mediterranean Basin—Current Situation and Risks for the Future, Plan Bleu, UNEP/MAP, Marseille, France, First Mediterranean Assessment Report (2020). 10.5281/zenodo.4768833

[2] Coll, M., Piroddi, C. & Steenbeek, J. The biodiversity of the mediterranean sea: Estimates, patterns, and threats. *PLoS ONE*. **5**, e11842. 10.1371/journal.pone.0011842 (2010). 20689844 10.1371/journal.pone.0011842 PMC2914016

[3] MedECC Climate and environmental change in the mediterranean Basin—Current situation and risks for the future. *Union Mediterranean Plan. Bleu UNEP/MAP Marseille France*. 10.5281/zenodo.4768833 (2020).

[4] Dos Santos, M. & Moncada, S. Development. In Climate and environmental change in the mediterranean Basin—Current situation and risks for the future. *Union Mediterranean*. 469–492. 10.5281/zenodo.7101111 (2020).

[5] Hassoun, A. E. R., Bantelman, A. & Canu, D. Ocean acidification research in the mediterranean sea: Status, trends and next steps. *Front. Mar. Sci.* **9** (892670). 10.3389/fmars.2022.892670 (2022).

[6] Henson, S. A. et al. Rapid emergence of climate change in environmental drivers of marine ecosystems, *Nat. Commun.* **8**(1), 1 (2017). 10.1038/ncomms14682 10.1038/ncomms14682 PMC5343512 28267144

[7] I.P.C.C. et al., Summary for Policymakers. In Climate Change and Land: an IPCC special report on climate change, desertification, land degradation, sustainable land management, food security, and greenhouse gas fluxes in terrestrial ecosystems (2019).

[8] Merheb, M., Moussa, R. & Abdallah, C. Hydrological response characteristics of mediterranean catchments at different time scales: A meta-analysis. *Hydrol. Sci. J.* **61**, 2520–2539. 10.1080/02626667.2016.1140174 (2016).

[9] Tanhua, T., Hainbucher, D. & Schroeder, K. The mediterranean sea system: A review and an introduction to the special issue. *Ocean Sci.* **9** (5), 789–803. 10.5194/os-9-789-2013 (2013).

[10] Cramer, W., Guiot, J. & Fader, M. Climate change and interconnected risks to sustainable development in the mediterranean. *Nat. Clim. Chang*. **8** (11), 972–980. 10.1038/s41558-018-0299-2 (2018).

[11] Chatzimentor, A., Doxa, A., Katsanevakis, S. & Mazaris, A. D. Are mediterranean marine threatened species at high risk by climate change? *Glob. Chang. Biol.* **29** (7), 1809–1821. 10.1111/gcb.16577 (2023). 10.1111/gcb.16577 36583369

[12] Álvarez, M. et al. Chapter 11 - Mediterranean sea general biogeochemistry, in Oceanography of the Mediterranean Sea, (eds Schroeder, K. & Chiggiato, J.) Elsevier, 387–451. 10.1016/B978-0-12-823692-5.00004-2. (2023).

[13] Rice, J. C. & Garcia, S. M. Fisheries, food security, climate change, and biodiversity: Characteristics of the sector and perspectives on emerging issues. *ICES J. Mar. Sci.* **68**(6), 1343–1353 (2011). 10.1093/icesjms/fsr041

[14] Barange, M. et al. *Impacts of Climate Change on Fisheries and Aquaculture. Synthesis of Current Knowledge, Adaptation, and Mitigation Options*. (2018).

[15] Intergovernmental Panel on Climate Change (IPCC). *The Ocean and Cryosphere in a Changing Climate: Special Report of the Intergovernmental Panel on Climate Change* (Cambridge University Press, 2022). 10.1017/9781009157964

[16] Cherif, S., Doblas-Miranda, E. & Lionello, P. Drivers of change. In Climate and Environmental Change in the Mediterranean Basin—Current Situation and Risks for the Future, *Union for the Mediterranean*. Plan Bleu, UNEP/MAP, Marseille, France, pp. 59–180, (2020). 10.5281/zenodo.7100601

[17] Cos, J., Doblas-Reyes, F. & Jury, M. The mediterranean climate change hotspot in the CMIP5 and CMIP6 projections. *Earth Syst. Dyn.* **13** (1), 321–340. 10.5194/esd-13-321-2022 (2022).

[18] Lionello, P. & Scarascia, L. The relation between climate change in the mediterranean region and global warming. *Reg. Envriron. Chang.* **18**, 1481–1493. 10.1007/s10113-018-1290-1 (2018).

[19] Pastor, F., Valiente, J. A. & Khodayar, S. A warming mediterranean: 38 years of increasing sea surface temperature. *Remote Sens.* **12** (17). 10.3390/rs12172687 (2020).

[20] Nabat, P., Somot, S. & Mallet, M. Direct and semi-direct aerosol radiative effect on the mediterranean climate variability using a coupled regional climate system model. *Clim. Dyn.* **44**, 1127–1155. 10.1007/s00382-014-2205-6 (2015).

[21] Darmaraki, S., Somot, S. & Sevault, F. Future evolution of marine heatwaves in the mediterranean sea. *Climat. Dyn.*, **53**, pp. 1371–1392 (2019a).

[22] Pisano, A., Marullo, S. & Artale, V. New evidence of mediterranean climate change and variability from sea surface temperature observations. *Remote Sens.* **12** (1). 10.3390/rs12010132 (2020).

[23] Fox-Kemper, B. et al. Ocean, cryosphere, and sea level change - Supplementary material, in Climate Change 2021: The Physical Science Basis. Contribution of Working Group I To the Sixth Assessment Report of the Intergovernmental Panel on Climate Change, (eds Masson-Delmotte, V., Zhai, P., Pirani, A., Connors, S. L., Péan, C., Berger, S., Caud, N., Chen, Y., Goldfarb, L., Gomis, M. I., Huang, M., Leitzell, K., Lonnoy, E., Matthews, J. B. R., Maycock, T. K., Waterfield, T., Yelekçi, Ö., Yu, R. & Zhou, B.) Cambridge University Press, (2021).

[24] Darmaraki, S., Somot, S. & Sevault, F. Past variability of mediterranean sea marine heatwaves. *Geophys. Res. Lett.* **46**, 16, pp. 9813–9823 (2019b). 10.1029/2019GL082933

[25] Vargas-Yáñez, M., Zunino, P. & Benali, A. How much is the Western mediterranean really warming and salting? *J. Geophys. Res. Oceans*. **115**, C04001 10.1029/2009JC005816 (2010).

[26] Skliris, N., Marsh, R. & Josey, S. A. Salinity changes in the world ocean since 1950 in relation to changing surface freshwater fluxes. *Clim. Dyn.* **43**, 709–736. 10.1007/s00382-014-2131-7 (2014).

[27] Schroeder, K., Chiggiato, J. & Bryden, H. L. Abrupt climate shift in the Western mediterranean sea. *Sci. Rep.* **6** (1). 10.1038/srep23009 (2016). 10.1038/srep23009 PMC4786855 26965790

[28] Soto-Navarro, J., Jordá, G. & Amores, Á. Evolution of mediterranean sea water properties under climate change scenarios in the Med-CORDEX ensemble. *Clim. Dyn.* **54**, 2135–2165. 10.1007/s00382-019-05105-4 (2020).

[29] Garrabou, J., Gómez-Gras, D. & Medrano, A. Marine heatwaves drive recurrent mass mortalities in the mediterranean sea. *Glob. Chang. Biol.* **28**, 5708–5725. 10.1111/gcb.16301 (2022). 10.1111/gcb.16301 PMC9543131 35848527

[30] Juza, M., Fernández-Mora, À. & Tintoré, J. Sub-regional marine heat waves in the mediterranean sea from observations: Long-term surface changes, sub-surface and coastal responses. *Front. Mar. Sci.* **9** (785771). 10.3389/fmars.2022.785771 (2022).

[31] Pastor, F. & Khodayar, S. Marine heat waves: Characterizing a major climate impact in the mediterranean. *Sci. Total Environ.* **861**, 160621. 10.1016/j.scitotenv.2022.160621 (2023). 10.1016/j.scitotenv.2022.160621 36462657

[32] Dayan, H., McAdam, R. & Juza, M. Marine heat waves in the mediterranean sea: An assessment from the surface to the subsurface to Meet National needs. *Front. Mar. Sci.* **10** (1045138). 10.3389/fmars.2023.1045138 (2023).

[33] Tramblay, Y. & Somot, S. Future evolution of extreme precipitation in the mediterranean. *Clim. Chang*. **151**, 289–302. 10.1007/s10584-018-2300-5 (2018).

[34] Lionello, P. & Scarascia, L. The relation of climate extremes with global warming in the mediterranean region and its North versus South contrast. *Reg. Envriron. Chang.* **20** (1). 10.1007/s10113-020-01610-z (2020).

[35] Powley, H. R., Krom, M. D. & Cappellen, P. Circulation and oxygen cycling in the mediterranean sea: Sensitivity to future climate change. *J. Geophys. Res. Oceans*. **121** (11), 8230–8247. 10.1002/2016JC012224 (2016).

[36] Ferreira, J. G., Andersen, J. H. & Borja, A. Overview of eutrophication indicators to assess environmental status within the European marine strategy framework directive. *Estuar. Coast. Shelf Sci.* **93** (2), 117–131. 10.1016/j.ecss.2011.03.014 (2011).

[37] Santinelli, C., Hansell, D. A. & d’Alcalà, M. R. Influence of stratification on marine dissolved organic carbon (DOC) dynamics: The mediterranean sea case. *Prog. Oceanogr.* **119**, 68–77. 10.1016/j.pocean.2013.06.001 (2013).

[38] Ngatia et al. Nitrogen and phosphorus eutrophication in marine ecosystems. *Monit. Mar. Pollut.*. 1–17. 10.5772/intechopen.81869 (2019).

[39] Goyet, C., Hassoun, A. E. R. & Gemayel, E. Thermodynamic forecasts of the mediterranean sea acidification. *Mediterr. Mar. Sci.* **17** (2), 508–518. 10.12681/mms.1487 (2016).

[40] Reale, M., Cossarini, G. & Lazzari, P. Acidification, deoxygenation, and nutrient and biomass declines in a warming mediterranean sea. *Biogeosciences* **19**, 4035–4065. 10.5194/bg-19-4035-2022 (2022).

[41] Solidoro, C., Cossarini, G. & Lazzari, P. Modeling carbon budgets and acidification in the mediterranean sea ecosystem under contemporary and future climate. *Front. Mar. Sci.* **8** (781522). 10.3389/fmars.2021.781522 (2022).

[42] Kwiatkowski, L. et al. Jul., Twenty-first century ocean warming, acidification, deoxygenation, and upper-ocean nutrient and primary production decline from CMIP6 model projections, *Biogeosciences* **17**(13), 3439–3470, (2020). 10.5194/bg-17-3439-2020

[43] Hassoun, A. E. R., Gemayel, E. & Krasakopoulou, E. Acidification of the Mediterranean Sea from anthropogenic carbon penetration, *Deep Sea Res. Part I Oceanogr. Res. Pap.* **102**, 1–15 (2015). 10.1016/j.dsr.2015.04.005

[44] McFadden, L., Spencer, T. & Nicholls, R. J. Broad-scale modelling of coastal wetlands: What is required? *Hydrobiologia* **577**, 5–15. 10.1007/s10750-006-0413-8 (2007).

[45] Satta, A., Puddu, M. & Venturini, S. Assessment of coastal risks to climate change related impacts at the regional scale: The case of the mediterranean region. *Int. J. Disaster Risk Reduct.* **24**, 284–296. 10.1016/j.ijdrr.2017.06.018 (2017).

[46] Ali, E., Cramer, W. & Carnicer, J. Cross-chapter paper 4: mediterranean region, in Climate Change 2022: Impacts, Adaptation and Vulnerability. Contribution of Working Group II To the Sixth Assessment Report of the Intergovernmental Panel on Climate Change [Pörtner, R. H.-O., T. D.C., and M., Eds., Cambridge, UK and New York, NY, USA: Cambridge University Press, 2233–2272. 10.1017/9781009325844.021. (2022).

[47] Zerbini, S., Raicich, F. & Prati, C. M. Sea-level change in the Northern mediterranean sea from long-period tide gauge time series. *Earth Sci. Rev.* **167**, 72–87. 10.1016/j.earscirev.2017.02.009 (2017).

[48] Wöppelmann, G. & Marcos, M. Coastal sea level rise in Southern Europe and the nonclimate contribution of vertical land motion. *J. Geophys. Res. Oceans*. **117**, C01007 10.1029/2011JC007469 (2012).

[49] Marcos, M., Wöppelmann, G. & Calafat, F. M. Mediterranean Sea level, in *Oceanography of the Mediterranean Sea*, K. Schroeder and J. Chiggiato, Eds., pp. 125–159. (2023). 10.1016/B978-0-12-823692-5.00012-1

[50] Slangen, A. B. A., Adloff, F. & Jevrejeva, S. A review of recent updates of sea-level projections at global and regional scales, in Integrative Study of the Mean Sea Level and its Components, (eds Cazenave, A., Champollion, N. & Paul, F.) (Springer, 2017). 10.1007/978-3-319-56490-6_17

[51] Liquete, C., Piroddi, C. & Macías, D. Ecosystem services sustainability in the mediterranean sea: Assessment of status and trends using multiple modelling approaches. *Sci. Rep.* **6** (1). 10.1038/srep34162 (2016). 10.1038/srep34162 PMC5043175 27686533

[52] Martín-López, B., Oteros-Rozas, E. & Cohen-Shacham, E. Ecosystem services supplied by Mediterranean Basin ecosystems. In *Routledge Handbook of Ecosystem Services*, M. Potschin, R. Haines-Young, and R. Fish, Eds., pp. 405–414. [Online]. (2016). https://www.taylorfrancis.com/chapters/edit/10.4324/9781315775302-35/ecosystem-services-supplied-mediterranean-basin-ecosystems-berta-mart%C3%ADn-l%C3%B3pez-elisa-oteros-rozas-emmanuelle-cohen-shacham-fernando-santos-mart%C3%ADn-marta-nieto-romero-claudia-carvalho-santos-jos%C3%A9-gonz%C3%A1lez-marina-garc%C3%ADa-llorente-keren-klass-ilse-geijzendorffer-carlos-montes-wolfgang-cramer

[53] Vafeidis, A., Neumann, B., Zimmerman, J. & MR9. : Analysis of land area and population in the low-elevation coastal zone (LECZ. London, GB, 2011. http://eprints.soton.ac.uk/id/eprint/207617

[54] Mastrandrea, M. D., Field, C. B. & Stocker, T. F. Guidance note for lead authors of the IPCC fifth assessment report on consistent treatment of uncertainties. 2010. https://www.ipcc.ch/site/assets/uploads/2018/05/uncertainty-guidance-note.pdf

[55] Lazzari, P., Mattia, G. & Solidoro, C. The impacts of climate change and environmental management policies on the trophic regimes in the mediterranean sea: Scenario analyses. *J. Mar. Syst.* **135**, 137–149. 10.1016/j.jmarsys.2013.06.005 (2014).

[56] Maugendre, L., Gattuso, J. P. & Louis, J. Effect of ocean warming and acidification on a plankton community in the NW mediterranean sea. *ICES J. Mar. Sci.* **72** (6), 1744–1755. 10.1093/icesjms/fsu161 (2015).

[57] Pulina, S., Brutemark, A. & Suikkanen, S. Effects of warming on a mediterranean phytoplankton community. *Web Ecol.* **16** (1), 89–92. 10.5194/we-16-89-2016 (2016).

[58] Gazeau et al. Limited impact of ocean acidification on phytoplankton community structure and carbon export in an oligotrophic environment: results from two short-term mesocosm studies in the mediterranean sea. *Estuar. Coast. Shelf Sci.*10.1016/j.ecss.2016.11.016 (2017).

[59] Benedetti, F., Guilhaumon, F. & Adloff, F. Investigating uncertainties in zooplankton composition shifts under climate change scenarios in the mediterranean sea. *Ecography* **41** (2), 345–360. 10.1111/ecog.02434 (2018).

[60] Moltó, V., Palmer, M. & Ospina-Álvarez, A. Projected effects of ocean warming on an iconic pelagic fish and its fishery. *Sci. Rep.* **11** (1). 10.1038/s41598-021-88171-1 (2021). 10.1038/s41598-021-88171-1 PMC8062520 33888813

[61] Maugendre, L., Gattuso, J. P. & Poulton, A. J. No detectable effect of ocean acidification on plankton metabolism in the NW oligotrophic mediterranean sea: Results from two mesocosm studies. *Estuar. Coast. Shelf Sci.* **186**, 89–99. 10.1016/j.ecss.2015.03.009 (2017).

[62] Herrmann, M., Estournel, C. & Adloff, F. Impact of climate change on the Northwestern mediterranean sea pelagic planktonic ecosystem and associated carbon cycle. *J. Geophys. Res. Oceans*. **119** (9), 5815–5836. 10.1002/2014JC010016 (2014).

[63] Macias, D. M., Garcia-Gorriz, E. & Stips, A. Productivity changes in the mediterranean sea for the twenty-first century in response to changes in the regional atmospheric forcing. *Front. Mar. Sci.* **2** (79). 10.3389/fmars.2015.00079 (2015).

[64] Stefanidou, N., Genitsaris, S. & Lopez-Bautista, J. Effects of heat shock and salinity changes on coastal mediterranean phytoplankton in a mesocosm experiment. *Mar. Biol.* **165**, 1–14. 10.1007/s00227-018-3415-y (2018).

[65] Moullec, F., Barrier, N. & Drira, S. An end-to-end model reveals losers and winners in a warming mediterranean sea. *Front. Mar. Sci.* **6** (345). 10.3389/fmars.2019.00345 (2019).

[66] Pagès, R. et al. Projected effects of climate-induced changes in hydrodynamics on the biogeochemistry of the mediterranean sea under the RCP 8.5 regional climate scenario. *Front. Mar. Sci.* **7**, 563615. 10.3389/fmars.2020.563615 (2020).

[67] Leeuwen, S. M. & Beecham, J. A. The mediterranean Rhodes gyre: Modelled impacts of climate change, acidification and fishing. *Mar. Ecol. Prog. Ser.* **690**, 31–50. 10.3354/meps14016 (2022).

[68] Accoroni, S., Romagnoli, T. & Penna, A. Ostreopsis Fattorussoi sp. Nov. (Dinophyceae), a new benthic toxic Ostreopsis species from the Eastern mediterranean sea. *J. Phycol.* **52** (6), 1064–1084. 10.1111/jpy.12464 (2016). 27633521 10.1111/jpy.12464

[69] Vila, M., Abós-Herràndiz, R. & Isern-Fontanet, J. Establishing the link between Ostreopsis cf. ovata blooms and human health impacts using ecology and epidemiology. *Sci. Mar.* **80**, 107–115. 10.3989/scimar.04395.08A (2016).

[70] Abboud-Abi Saab, M. & Hassoun, A. E. R. Effects of organic pollution on environmental conditions and the phytoplankton community in the central Lebanese coastal waters with special attention to toxic algae. *Reg. Stud. Mar. Sci.* **10**, 38–51. 10.1016/j.rsma.2017.01.003 (2017).

[71] Hassoun, A. E. R., Ujević, I. & Mahfouz, C. Occurrence of Domoic acid and Cyclic Imines in marine biota from Lebanon-Eastern mediterranean sea. *Sci. Total Environ.* **755**, 142542. 10.1016/j.scitotenv.2020.142542 (2021). 10.1016/j.scitotenv.2020.142542 33035983

[72] Soulié, T., Vidussi, F. & Mas, S. Functional and structural responses of plankton communities toward consecutive experimental heatwaves in mediterranean coastal waters. *Sci. Rep.* **13** (1). 10.1038/s41598-023-35311-4 (2023). 10.1038/s41598-023-35311-4 PMC10192389 37198394

[73] Howes, E. L., Joos, F. & Eakin, C. M. An updated synthesis of the observed and projected impacts of climate change on the chemical, physical and biological processes in the oceans. *Front. Mar. Sci.* **2** (36). 10.3389/fmars.2015.00036 (2015).

[74] Temino-Boes, R., García-Bartual, R. & Romero, I. Future trends of dissolved inorganic nitrogen concentrations in Northwestern mediterranean coastal waters under climate change. *J. Environ. Manag.* **282** (111739). 10.1016/j.jenvman.2020.111739 (2021). 10.1016/j.jenvman.2020.111739 33461817

[75] Totti, C., Romagnoli, T. & Accoroni, S. Phytoplankton communities in the Northwestern Adriatic sea: Interdecadal variability over a 30-years period (1988–2016) and relationships with meteoclimatic drivers. *J. Mar. Syst.* **193**, 137–153. 10.1016/j.jmarsys.2019.01.007 (2019).

[76] Volpe, G., Nardelli, B. B. & Cipollini, P. V. A., G. B., and P., Seasonal to interannual phytoplankton response to physical processes in the Mediterranean Sea from satellite observations. *Remote Sens. Environ. Remote Sensing Urban Environ.* **117**(4), 223–235 (2012). 10.1016/j.rse.2011.09.020Waterkeyn

[77] Macias, D., Garcia-Gorriz, E. & Stips, A. Deep winter convection and phytoplankton dynamics in the NW mediterranean sea under present climate and future (horizon 2030) scenarios. *Sci. Rep.* **8** (1). 10.1038/s41598-018-24965-0 (2018). 10.1038/s41598-018-24965-0 PMC5919909 29700363

[78] Meier, K. J. S., Beaufort, L. & Heussner, S. The role of ocean acidification in emiliania huxleyi coccolith thinning in the mediterranean sea. *Biogeosciences* **11** (10), 2857–2869. 10.5194/bg-11-2857-2014 (2014).

[79] Mallo, M., Ziveri, P. & Mortyn, P. G. Low planktic foraminiferal diversity and abundance observed in a spring 2013 west–east mediterranean sea plankton Tow transect. *Biogeosciences* **14**, 2245–2266. 10.5194/bg-14-2245-201Mancino (2017).

[80] D’Amario, B., Pérez, C. & Grelaud, M. Coccolithophore community response to ocean acidification and warming in the Eastern mediterranean sea: Results from a mesocosm experiment. *Sci. Rep.* **10** (1). 10.1038/s41598-020-69519-5 (2020). 10.1038/s41598-020-69519-5 PMC7387480 32724047

[81] Schickele, A., Goberville, E. & Leroy, B. European small pelagic fish distribution under global change scenarios. *Fish Fish.* **22** (1), 212–225. 10.1111/faf.12515 (2020).

[82] Coll, M. et al. The mediterranean sea under siege: Spatial overlap between marine biodiversity, cumulative threats and marine reserves. *Glob. Ecol. Biogeogr.* **21** (4), 465–480. 10.1111/j.1466-8238.2011.00697.x (2012).

[83] Tsikliras, A. C., Dinouli, A., Tsiros, V. Z. & Tsalkou, E. The mediterranean and black sea fisheries at risk from overexploitation. *PLoS ONE*. **10** (3), e0121188. 10.1371/journal.pone.0121188 (Mar. 2015). 10.1371/journal.pone.0121188 PMC4368760 25793975

[84] Richon, C., Dutay, J. C. & Bopp, L. Biogeochemical response of the mediterranean sea to the transient SRES-A2 climate change scenario. *Biogeosciences* **16** (1), 135–165. 10.5194/bg-16-135-2019 (2019).

[85] Hall-Spencer, J. M., Rodolfo-Metalpa, R. & Martin, S. Volcanic carbon dioxide vents show ecosystem effects of ocean acidification. *Nature* **454**, 96–99. 10.1038/nature07051 (2008). 18536730 10.1038/nature07051

[86] Martin, S. & Gattuso, J. P. Response of mediterranean coralline algae to ocean acidification and elevated temperature. *Glob. Chang. Biol.* **15** (8), 2089–2100. 10.1111/j.1365-2486.2009.01874.x (2009).

[87] Movilla, J., Calvo, E. & Pelejero, C. Calcification reduction and recovery in native and non-native mediterranean corals in response to ocean acidification. *J. Exp. Mar. Biol. Ecol.* **438**, 144–153. 10.1016/j.jembe.2012.09.014Movilla (2012).

[88] Bramanti, L., Movilla, J. & Guron, M. Detrimental effects of ocean acidification on the economically important mediterranean red coral (Corallium rubrum. *Glob. Chang. Biol.* **19** (6), 1897–1908. 10.1111/gcb.12171 (2013). 10.1111/gcb.12171 23505003

[89] Movilla Orejas, J. & Calvo, C. Differential response of two mediterranean cold-water coral species to ocean acidification. *Coral Reefs*. **33**, 675–686. 10.1007/s00338-014-1159-9 (2014).

[90] Fine, M., Tsadok, R. & Meron, D. Environmental sensitivity of Neogoniolithon brassica-florida associated with vermetid reefs in the mediterranean sea. *ICES J. Mar. Sci.* **74** (4), 1074–1082. 10.1093/icesjms/fsw167 (2017).

[91] Prada, F., Caroselli, E. & Mengoli, S. Ocean warming and acidification synergistically increase coral mortality. *Sci. Rep.* **7** (1). 10.1038/srep40842 (2017). 10.1038/srep40842 PMC5244398 28102293

[92] Marchini, C., Tortorelli, G. & Guidi, E. Reproduction of the azooxanthellate coral Caryophyllia inornata is not affected by temperature along an 850 Km gradient on the Western Italian Coast. *Front. Mar. Sci.* **6** (785). 10.3389/fmars.2019.00785 (2020).

[93] Vitelletti, M. L., Manea, E. & Bongiorni, L. Modelling distribution and fate of coralligenous habitat in the Northern Adriatic sea under a severe climate change scenario. *Front. Mar. Sci.* **10** (1050293). 10.3389/fmars.2023.1050293 (2023).

[94] Gómez-Gras, D., Linares, C. & Caralt, S. Response diversity in mediterranean coralligenous assemblages facing climate change: Insights from a multispecific thermotolerance experiment. *Ecol. Evol.* **9** (7), 4168–4180. 10.1002/ece3.5045 (2019). 31015996 10.1002/ece3.5045 PMC6468064

[95] Carbonne et al. Early life stages of a mediterranean coral are vulnerable to ocean warming and acidification. *Biogeosciences* **19**, 4767–4777. 10.5194/bg-19-4767-2022 (2022).

[96] Zunino, S., Canu, D. M., Bandelj, V. & Solidoro, C. Effects of ocean acidification on benthic organisms in the mediterranean sea under realistic Climatic scenarios: A meta-analysis. *Reg. Stud. Mar. Sci.* **10**, 86–96. 10.1016/j.rsma.2016.12.011 (Feb. 2017).

[97] Zunino, S., Canu, D. M., Zupo, V. & Solidoro, C. Direct and indirect impacts of marine acidification on the ecosystem services provided by coralligenous reefs and seagrass systems. *Global Ecol. Conserv.* **18**, e00625. 10.1016/j.gecco.2019.e00625 (Apr. 2019).

[98] Zunino, S., Libralato, S., Melaku Canu, D., Prato, G. & Solidoro, C. Impact of ocean acidification on ecosystem functioning and services in habitat-forming species and marine ecosystems. *Ecosystems* **24**(7), 1561–1575 (2021). 10.1007/s10021-021-00601-3

[99] Martin, S., Cohu, S. & Vignot, C. One-year experiment on the physiological response of the mediterranean crustose coralline alga, Lithophyllum cabiochae, to elevated pCO2 and temperature. *Ecol. Evol.* **3** (3), 676–693. 10.1002/ece3.475 (2013). 23533024 10.1002/ece3.475 PMC3605855

[100] Nash, M. C., Martin, S. & Gattuso, J. P. Mineralogical response of the mediterranean crustose coralline Alga Lithophyllum Cabiochae to near-future ocean acidification and warming. *Biogeosciences* **13** (21), 5937–5945. 10.5194/bg-13-5937-2016 (2016).

[101] Kamenos, N. A., Perna, G. & Gambi, M. C. Coralline algae in a naturally acidified ecosystem persist by maintaining control of skeletal mineralogy and size, *Proc. R. Soc. B Biol. Sci.* **283**(1840) (2016). 10.1098/rspb.2016.1159 10.1098/rspb.2016.1159 PMC5069505 27733544

[102] Nannini, M., Marchi, L. & Lombardi, C. Effects of thermal stress on the growth of an intertidal population of Ellisolandia elongata (Rhodophyta) from N–W mediterranean sea. 2015. 10.1016/j.marenvres.2015.05.005 10.1016/j.marenvres.2015.05.005 26004519

[103] Gamliel, I., Buba, Y. & Guy-Haim, T. Incorporating physiology into species distribution models moderates the projected impact of warming on selected mediterranean marine species. *Ecography* **43** (7), 1090–1106. 10.1111/ecog.04423 (2020).

[104] Marchini, A., Ragazzola, F. & Vasapollo, C. Intertidal mediterranean coralline algae habitat is expecting a shift toward a reduced growth and a simplified associated fauna under climate change. *Front. Mar. Sci.* **6**10.3389/fmars.2019.00106 (2019).

[105] Cox, T. E., Nash, M. & Gazeau, F. Effects of in situ CO_2_ enrichment on Posidonia oceanica epiphytic community composition and mineralogy. *Mar. Biol.* **164**, 1–16. 10.1007/s00227-017-3136-7 (2017).27980349

[106] Porzio, L., Buia, M. C. & Hall-Spencer, J. M. Effects of ocean acidification on macroalgal communities. *J. Exp. Mar. Biol. Ecol.* **400**, 1–2. 10.1016/j.jembe.2011.02.011 (2011).

[107] Porzio, L., Garrard, S. L. & Buia, M. C. The effect of ocean acidification on early algal colonization stages at natural CO_2_ vents. *Mar. Biol.* **160**, 2247–2259. 10.1007/s00227-013-2251-3 (2013).

[108] Chefaoui, R. M., Duarte, C. M. & Serrão, E. A. Dramatic loss of seagrass habitat under projected climate change in the mediterranean sea. *Glob. Chang. Biol.* **24** (10), 4919–4928. 10.1111/gcb.14401 (2018). 10.1111/gcb.14401 30006980

[109] Litsi-Mizan, V., Efthymiadis, P. T. & Gerakaris, V. Decline of seagrass (Posidonia oceanica) production over two decades in the face of warming of the Eastern mediterranean sea. *New Phytol.* **239** (6), 2126–2137. 10.1111/nph.19084 (2023). 37366062 10.1111/nph.19084

[110] Jordà, G., Marbà, N. & Duarte, C. Mediterranean seagrass vulnerable to regional climate warming. *Nat. Clim. Chang*. **2** (11), 821–824. 10.1038/nclimate1533 (2012).

[111] Marbà, N. & Duarte, C. M. Mediterranean warming triggers seagrass (Posidonia oceanica) shoot mortality. *Glob. Chang. Biol.* **16** (8), 2366–2375. 10.1111/j.1365-2486.2009.02130.x (2010).

[112] Llabrés, E., Blanco-Magadán, A. & Sales, M. Effect of global warming on Western mediterranean seagrasses: A preliminary agent-based modelling approach. *Mar. Ecol. Prog. Ser.* **710**, 43–56. 10.3354/meps14298 (2023).

[113] Ontoria, Y., Gonzalez-Guedes, E. & Sanmartí, N. Interactive effects of global warming and eutrophication on a fast-growing mediterranean seagrass. *Mar. Environ. Res.* **145**, 27–38. 10.1016/j.marenvres.2019.02.002 (2019). 30795849 10.1016/j.marenvres.2019.02.002

[114] Stipcich, P., Apostolaki, E. T. & Chartosia, N. Assessment of Posidonia oceanica traits along a temperature gradient in the mediterranean sea shows impacts of marine warming and heat waves. *Front. Mar. Sci.* **9** (895354). 10.3389/fmars.2022.895354 (2022).

[115] Martínez-Abraín, A., Castejón-Silvo, I. & Roiloa, S. Foreseeing the future of Posidonia oceanica meadows by accounting for the past evolution of the mediterranean sea. *ICES J. Mar. Sci.* **79** (10), 2597–2599. 10.1093/icesjms/fsac212 (2022).

[116] Hendriks, I. E., Olsen, Y. S. & Duarte, C. M. Light availability and temperature, not increased CO_2_, will structure future meadows of Posidonia oceanica. *Aquat. Bot.* **139**, 32–36. 10.1016/j.aquabot.2017.02.004 (2017).

[117] Beca-Carretero, P., Teichberg, M. & Winters, G. Projected rapid habitat expansion of tropical seagrass species in the mediterranean sea as climate change progresses. *Front. Plant Sci.* **11** (555376). 10.3389/fpls.2020.555376 (2020). 10.3389/fpls.2020.555376 PMC7701102 33304358

[118] D’Amen, M. & Azzurro, E. Lessepsian fish invasion in mediterranean marine protected areas: A risk assessment under climate change scenarios. *ICES J. Mar. Sci.* **77** (1), 388–397. 10.1093/icesjms/fsz207 (2020).

[119] Stavrakidis-Zachou, O., Lika, K. & Anastasiadis, P. Projecting climate change impacts on mediterranean finfish production: A case study in Greece. *Clim. Chang*. **165** (3). 10.1007/s10584-021-03096-y (2021).

[120] Ben Lamine, E., Schickele, A. & Goberville, E. Expected contraction in the distribution ranges of demersal fish of high economic value in the mediterranean and European seas. *Sci. Rep.* **12** (1). 10.1038/s41598-022-14151-8 (2022). 10.1038/s41598-022-14151-8 PMC9203508 35710852

[121] Lima, A. R., Baltazar-Soares, M. & Garrido, S. Forecasting shifts in habitat suitability across the distribution range of a temperate small pelagic fish under different scenarios of climate change. *Sci. Total Environ.* **804** (150167). 10.1016/j.scitotenv.2021.150167 (2022). 10.1016/j.scitotenv.2021.150167 34798731

[122] Tsagarakis, K., Libralato, S. & Giannoulaki, M. Drivers of the North Aegean sea ecosystem (Eastern Mediterranean) through time: Insights from multidecadal retrospective analysis and future simulations. *Front. Mar. Sci.* **9** (919793). 10.3389/fmars.2022.919793 (2022).

[123] van Leeuwen, S. M., Beecham, J. A., García-García, L. & Thorpe, R. The Mediterranean Rhodes Gyre: modelled impacts of climate change, acidification and fishing. *Mar. Ecol. Progr. Ser*. 10.3354/meps14016

[124] Loya-Cancino, K. F., Ángeles-González, L. E. & Yañez-Arenas, C. Predictions of current and potential global invasion risk in populations of Lionfish (Pterois volitans and Pterois miles) under climate change scenarios. *Mar. Biol.* **170** (3). 10.1007/s00227-023-04174-8 (2023).

[125] Dimitriadis, C., Galanidi, M. & Zenetos, A. Updating the occurrences of Pterois miles in the mediterranean sea, with considerations on thermal boundaries and future range expansion. *Mediterr. Mar. Sci.* **21** (1), 62–69. 10.12681/mms.21845 (2020).

[126] Colloca, F., Scarcella, G. & Libralato, S. Recent trends and impacts of fisheries exploitation on mediterranean stocks and ecosystems. *Front. Mar. Sci.* **4**10.3389/fmars.2017.00244 (Aug. 2017).

[127] Albouy, C., Guilhaumon, F. & Leprieur, F. Projected climate change and the changing biogeography of coastal mediterranean fishes. *J. Biogeogr.* **40** (3), 534–547. 10.1111/jbi.12013 (2013).

[128] Maynou, F., Sabatés, A. & Ramírez-Romero, E. Future distribution of early life stages of small pelagic fishes in the Northwestern mediterranean. *Clim. Chang*. **161** (4), 567–589. 10.1007/s10584-020-02723-4 (2020).

[129] Samperio-Ramos, G., Olsen, Y. S. & Tomas, F. Ecophysiological responses of three mediterranean invasive seaweeds (Acrothamnion preissii, Lophocladia lallemandii and Caulerpa cylindracea) to experimental warming. *Mar. Pollut. Bull.* **96**, 1–2. 10.1016/j.marpolbul.2015.05.024 (2015). 25986653 10.1016/j.marpolbul.2015.05.024

[130] Buonomo, R., Chefaoui, R. M. & Lacida, R. B. Predicted extinction of unique genetic diversity in marine forests of Cystoseira spp. *Mar. Environ. Res.* **138**, 119–128. 10.1016/j.marenvres.2018.04.013 (2018). 29716751 10.1016/j.marenvres.2018.04.013

[131] Thrush, S. F., Chiantore, M. & Asnaghi, V. Habitat–diversity relationships in Rocky shore algal turf infaunal communities. *Mar. Ecol. Prog Ser.* **424**, 119–132. 10.3354/meps08960 (2011).

[132] Araújo, R. M., Assis, J. & Aguillar, R. Status, trends and drivers of Kelp forests in Europe: An expert assessment. *Biodivers. Conserv.* **25**, 1319–1348. 10.1007/s10531-016-1141-7 (2016).

[133] Coma, R., Ribes, M. & Serrano, E. Global warming-enhanced stratification and mass mortality events in the Mediterranean, *Proc. Natl. Acad. Sci* **106**, 6176–6181 (2009). 10.1073/pnas.0805801106 10.1073/pnas.0805801106 PMC2669359 19332777

[134] Bennett, S., Vaquer-Sunyer, R. & Jordá, G. Thermal performance of seaweeds and seagrasses across a regional climate gradient. *Front. Mar. Sci.* **9**, 733315. 10.3389/fmars.2022.733315 (2022).

[135] Almpanidou, V., Markantonatou, V. & Mazaris, A. D. Thermal heterogeneity along the migration corridors of sea turtles: Implications for climate change ecology. *J. Exp. Mar. Biol. Ecol.* **520**, 151223. 10.1016/j.jembe.2019.151223 (2019).

[136] Chatzimentor, A., Almpanidou, V. & Doxa, A. Projected redistribution of sea turtle foraging areas reveals important sites for conservation. *Clim. Chang. Ecol.* **2** (100038). 10.1016/j.ecochg.2021.100038 (2021).

[137] Albouy, C., Delattre, V. & Donati, G. Global vulnerability of marine mammals to global warming. *Sci. Rep.* **10** (1). 10.1038/s41598-019-57280-3 (2020). 10.1038/s41598-019-57280-3 PMC6969058 31953496

[138] van Weelden, C., Towers, J. R. & Bosker, T. Impacts of climate change on cetacean distribution, habitat and migration. *Clim. Chang. Ecol.* **1** (100009). 10.1016/j.ecochg.2021.100009 (2021).

[139] Mancino, C., Canestrelli, D. & Maiorano, L. Going west: Range expansion for loggerhead sea turtles in the Mediterranean Sea under climate change. *Glob. Ecol. Conserv.* **38**, e02264 (2022). 10.1016/j.gecco.2022.e02264

[140] Santostasi, N. L., Bonizzoni, S. & Gimenez, O. Common dolphins in the Gulf of corinth are critically endangered. *Aquat. Conserv. Mar. Freshw. Ecosyst.* **31**, 101–109. 10.1002/aqc.2963 (2021).

[141] Frantzis, A., Alexiadou, P. & Paximadis, G. Current knowledge of the cetacean fauna of the Greek seas. *J. Cetacean Res. Manag.* **5**, 219–232. 10.47536/jcrm.v5i3.801 (2023).

[142] Grose, S. O., Pendleton, L. & Leathers, A. Climate change will re-draw the map for marine megafauna and the people who depend on them. *Front. Mar. Sci.* **7** (547). 10.3389/fmars.2020.00547 (2020).

[143] Haubrock, P. J., Innocenti, G. & Mueller, S. A. Prey availability and community composition: Diet analysis of the black angler fish Lophius budegassa spinola, 1807 in the south-eastern mediterranean sea. *Reg. Stud. Mar. Sci*. **33**, 100940, (2020). 10.1016/j.rsma.2019.100940

[144] Enríquez, A. R., Marcos, M. & Álvarez-Ellacuría, A. Changes in beach shoreline due to sea level rise and waves under climate change scenarios: Application to the Balearic Islands (western mediterranean. *Nat. Hazards Earth Syst. Sci.* **17** (7), 1075–1089. 10.5194/nhess-17-1075-2017 (2017).

[145] Monioudi, I. N., Velegrakis, A. F. & Chatzipavlis, A. E. Assessment of Island beach erosion due to sea level rise: The case of the Aegean Archipelago (Eastern mediterranean. *Nat. Hazards Earth Syst. Sci.* **17** (3), 449–466. 10.5194/nhess-17-449-2017 (2017).

[146] Rizzi, J., Torresan, S. & Zabeo, A. Assessing storm surge risk under future sea-level rise scenarios: A case study in the North Adriatic Coast. *J. Coastal. Conserv.* **21**, 453–471. 10.1007/s11852-017-0517-5 (2017).

[147] Sanuy, M., Duo, E. & Jäger, W. S. Linking source with consequences of coastal storm impacts for climate change and risk reduction scenarios for mediterranean sandy beaches. *Nat. Hazards Earth Syst. Sci.* **18** (7), 1825–1847. 10.5194/nhess-18-1825-2018 (2018).

[148] Varela, M. R., Patrício, A. R. & Anderson, K. Assessing climate change associated sea-level rise impacts on sea turtle nesting beaches using drones, photogrammetry and a novel GPS system. *Glob. Chang. Biol.* **25** (2), 753–762. 10.1111/gcb.14526 (2019). 10.1111/gcb.14526 30430701

[149] Antonioli, F., Falco, G. & Lo Presti, V. Relative sea-level rise and potential submersion risk for 2100 on 16 coastal plains of the mediterranean sea. *Water* **12** (8). 10.3390/w12082173 (2020).

[150] Anzidei, M., Scicchitano, G. & Scardino, G. Relative sea-level rise scenario for 2100 along the Coast of South Eastern Sicily (Italy) by InSAR data, satellite images and high-resolution topography. *Remote Sens.* **13** (6). 10.3390/rs13061108 (2021).

[151] Thiéblemont, R., Le Cozannet, G. & Rohmer, J. Deep uncertainties in shoreline change projections: An extra-probabilistic approach applied to sandy beaches. *Nat. Hazards Earth Syst. Sci.* **21** (7), 2257–2276. 10.5194/nhess-21-2257-2021 (2021).

[152] Rizzo, A., Vandelli, V. & Gauci, C. Potential sea level rise inundation in the mediterranean: From susceptibility assessment to risk scenarios for policy action. *Water* **14** (416). 10.3390/w14030416 (2022).

[153] Filippaki, E., Tsakalos, E. & Kazantzaki, M. Forecasting impacts on vulnerable shorelines: Vulnerability assessment along the coastal zone of Messolonghi area—Western Greece. *Climate* **11** (1). 10.3390/cli11010024 (2023).

[154] Vandelli, V., Sarkar, N. & Micallef, A. S. Coastal inundation scenarios in the north-eastern sector of the Island of Gozo (Malta, mediterranean Sea) as a response to sea level rise. *J. Maps*. **19** (1). 10.1080/17445647.2022.2145918 (2023).

[155] Monioudi, I. N. et al. Climate change—induced hazards on touristic Island beaches: Cyprus, Eastern mediterranean. *Front. Mar. Sci.* **10**10.3389/fmars.2023.1188896 (Jul. 2023).

[156] Prisco, I., Carboni, M. & Acosta, A. T. The fate of threatened coastal Dune habitats in Italy under climate change scenarios. *PLoS One*. **8** (7). 10.1371/journal.pone.0068850 (2013). 10.1371/journal.pone.0068850 PMC3706318 23874787

[157] Sharaan, M. & Udo, K. Projections of future beach loss along the mediterranean coastline of Egypt due to sea-level rise. *Appl. Ocean Res.* **94** (101972). 10.1016/j.apor.2019.101972 (2020).

[158] Sánchez-Artús, X., Gracia, V. & Espino, M. Present and future flooding and erosion along the NW Spanish mediterranean Coast. *Front. Mar. Sci.* **10** (1125138). 10.3389/fmars.2023.1125138 (2023).

[159] Scapini, F., Innocenti Degli, E. & Defeo, O. Behavioral adaptations of sandy beach macrofauna in face of climate change impacts: A conceptual framework. *Estuar. Coast Shelf Sci.* **225** (19), 106236. 10.1016/j.ecss.2019.05.018Scardino (2019).

[160] Rilov, G., David, N. & Guy-Haim, T. Sea level rise can severely reduce biodiversity and community net production on Rocky Shores. *Sci. Total Environ.* **791** (148377). 10.1016/j.scitotenv.2021.148377 (2021). 10.1016/j.scitotenv.2021.148377 34412382

[161] Bonello, G., Carpi, L. & Mucerino, L. Sea-level change and the supralittoral environment: Potential impact on a splashpool habitat on the Ligurian Coast (NW mediterranean. *J. Biol. Res.-Bollettino Della Società Italiana Di Biol. Sper.* **95** (2). 10.4081/jbr.2022.10485 (2022).

[162] Lo Presti, V., Antonioli, F. & Casalbore, D. Geohazard assessment of the north-eastern Sicily continental margin (SW Mediterranean): Coastal erosion, sea-level rise and retrogressive canyon head dynamics. *Mar. Geophys. Res.* **43** (1). 10.1007/s11001-021-09463-9 (2022).

[163] Milazzo, M., Rodolfo-Metalpa, R. & Chan, V. B. S. Ocean acidification impairs vermetid reef recruitment. *Sci. Rep.* **4** (1). 10.1038/srep04189 (2014). 10.1038/srep04189 PMC5379440 24577050

[164] Freitas, D., Borges, D. & Arenas, F. Forecasting distributional shifts of Patella spp. In the Northeast Atlantic ocean, under climate change. *Mar. Environ. Res.* **186** (105945). 10.1016/j.marenvres.2023.105945 (2023). 10.1016/j.marenvres.2023.105945 36907078

[165] Rizzi, J., Gallina, V. & Torresan, S. Regional risk assessment addressing the impacts of climate change in the coastal area of the Gulf of Gabes (Tunisia. *Sustain. Sci.* **11**, 455–476. 10.1007/s11625-015-0344-2 (2016).

[166] Mastrocicco, M., Busico, G. & Colombani, N. Modelling actual and future seawater intrusion in the variconi coastal wetland (Italy) due to climate and landscape changes. *Water* **11** (7). 10.3390/w11071502 (2019).

[167] Estrela-Segrelles, C., Gómez-Martinez, G. & Pérez-Martín, M. Á. Risk assessment of climate change impacts on mediterranean coastal wetlands. Application in Júcar river basin district (Spain. *Sci. Total Environ.* **790** (148032). 10.1016/j.scitotenv.2021.148032 (2021). 10.1016/j.scitotenv.2021.148032 34098275

[168] Ramírez, F., Rodríguez, C. & Seoane, J. How will climate change affect endangered mediterranean waterbirds? *PLoS ONE*. **13** (2). 10.1371/journal.pone.0192702 (2018). 10.1371/journal.pone.0192702 PMC5811028 29438428

[169] Lefebvre, G., Redmond, L. & Germain, C. Predicting the vulnerability of seasonally-flooded wetlands to climate change across the mediterranean basin. *Sci. Total Environ.* **692**, 546–555. 10.1016/j.scitotenv.2019.07.263 (2019). 31351296 10.1016/j.scitotenv.2019.07.263

[170] Herbert, E. R., Boon, P. & Burgin, A. J. A global perspective on wetland salinization: Ecological consequences of a growing threat to freshwater wetlands. *Ecosphere* **6** (10), 1–43. 10.1890/ES14-00534.1 (2015).

[171] Waterkeyn, A., Vanschoenwinkel, B., Grillas, P. & Brendoncka, L. Effect of salinity on seasonal community patterns of mediterranean temporary wetland crustaceans: A mesocosm study. *Limnol. Oceanogr.* **55** (4), 1712–1722. 10.4319/lo.2010.55.4.1712 (2010).

[172] Ben Haj, S. C. D., and L. A., Sub-regional report on vulnerability and impacts of climate change on marine and coastal biological diversity in the Mediterranean Arab Countries. UNEP-MAP RAC/SPA, Tunis, [Online]. (2009). https://rac-spa.org/sites/default/files/doc_climate_change/ccc_med_arab.pdf

[173] Shaltout, M., Tonbol, K. & Omstedt, A. Sea-level change and projected future flooding along the Egyptian mediterranean Coast. *Oceanologia* **57** (4), 293–307. 10.1016/j.oceano.2015.06.004 (2015).

[174] Lionello, P., Nicholls, R. J. & Umgiesser, G. Venice flooding and sea level: Past evolution, present issues, and future projections (introduction to the special issue. *Nat. Hazards Earth Syst. Sci.* **21** (8), 2633–2641. 10.5194/nhess-21-2633-2021 (2021).

[175] Jeunesse, I., Cirelli, C. & Sellami, H. Is the governance of the thau coastal lagoon ready to face climate change impacts? *Ocean. Coastal. Manag.* **118**, 234–246. 10.1016/j.ocecoaman.2015.05.014 (2015).

[176] Lloret, J., Marín, A. & Marín-Guirao, L. Is coastal lagoon eutrophication likely to be aggravated by global climate change? Estuarine. *Coastal. Shelf Sci.* **78** (2), 403–412. 10.1016/j.ecss.2008.01.003 (2008).

[177] Abd-Elhamid, H. F., Zeleňáková, M. & Barańczuk, J. Historical trend analysis and forecasting of shoreline change at the nile Delta using RS data and GIS with the DSAS tool. *Remote Sens.* **15** (7). 10.3390/rs15071737 (2023).

[178] Sánchez-Arcilla, A., Jiménez, J. A. & Valdemoro, H. I. Implications of Climatic change on Spanish mediterranean low-lying coasts: The Ebro delta case. *J. Coastal Res.* **24** (2), 306–316. 10.2112/07A-0005.1 (2008).

[179] Range, P., Chícharo, M. A. & Ben-Hamadou, R. Impacts of CO_2_-induced seawater acidification on coastal mediterranean bivalves and interactions with other Climatic stressors. *Reg. Envriron. Chang.* **14**, 19–30. 10.1007/s10113-013-0478-7 (2014).

[180] Simantiris, N. & Avlonitis, M. Effects of future climate conditions on the zooplankton of a mediterranean coastal lagoon. *Estuar. Coast. Shelf Sci.* **282** (108231). 10.1016/j.ecss.2023.108231 (2023).

[181] Day, J. W., Ibáñez, C. & Pont, D. Status and sustainability of mediterranean deltas: The case of the ebro, rhône, and Po deltas and Venice lagoon. pp. 237–249, (2019). 10.1016/B978-0-12-814003-1.00014-9

[182] Frihy, O. E. & El-Sayed, M. K. Vulnerability risk assessment and adaptation to climate change induced sea level rise along the Mediterranean coast of Egypt, *Mitigation Adaptation Strateg. Global Chang.* **18**, 1215–1237, (2013). 10.1007/s11027-012-9418-y

[183] Deininger, A., Faithfull, C. L. & Lange, K. Simulated terrestrial runoff triggered a phytoplankton succession and changed Seston stoichiometry in coastal lagoon mesocosms. *Mar. Environ. Res.* **119**, 40–50. 10.1016/j.marenvres.2016.05.001 (2016). 27209121 10.1016/j.marenvres.2016.05.001

[184] Cardoch, L., Day, J. W. & Ibàñez, C. Net primary productivity as an Indicator of sustainability in the Ebro and Mississippi deltas. *Ecol. Appl.* **12**, 1044–1055. 10.1890/1051-0761(2002)012 (2002).

[185] Scardino, G. et al. Jan., The impact of future sea-level rise on low-lying subsiding coasts: A case study of Tavoliere Delle Puglie (Southern Italy), *Remote Sens.* **14** (19), 19 (2022). 10.3390/rs14194936

[186] Strain, E. M., Belzen, J. & Comandini, P. The role of changing climate in driving the shift from perennial grasses to annual succulents in a mediterranean saltmarsh. *J. Ecol.* **105** (5), 1374–1385. 10.1111/1365-2745.12799 (2017).

[187] Borges, F. O., Santos, C. P. & Paula, J. R. Invasion and extirpation potential of native and invasive Spartina species under climate change. *Front. Mar. Sci.* **8** (696333). 10.3389/fmars.2021.696333 (2021).

[188] O’Leary, J. K., Micheli, F. & Airoldi, L. The resilience of marine ecosystems to Climatic disturbances. *BioScience* **67** (3), 208–220 (2017).

[189] Carneiro, J. F., Boughriba, M. & Correia, A. Evaluation of climate change effects in a coastal aquifer in Morocco using a density-dependent numerical model. *Environ. Earth Sci.* **61**, 241–252. 10.1007/s12665-009-0339-3 (2010).

[190] Sefelnasr, A. & Sherif, M. Impacts of seawater rise on seawater intrusion in the nile Delta aquifer, Egypt. *Groundwater* **52** (2), 264–276. 10.1111/gwat.12058 (2014). 10.1111/gwat.12058 23600466

[191] Romanazzi, A., Gentile, F. & Polemio, M. Modelling and management of a mediterranean karstic coastal aquifer under the effects of seawater intrusion and climate change. *Environ. Earth Sci.* **74**, 115–128. 10.1007/s12665-015-4423-6 (2015).

[192] Al-Najjar, H., Ceribasi, G. & Dogan, E. GCMs simulation-based assessment for the response of the mediterranean Gaza coastal aquifer to climate-induced changes. *J. Water Clim. Chang*. **13** (6), 2278–2297. 10.2166/wcc.2022.339 (2022).

[193] Schorpp, L., Dall’Alba, V. & Renard, P. Hydrogeological modeling of the Roussillon coastal aquifer (France): Stochastic inversion and analysis of future stresses. *Environ. Earth Sci.* **82** (9). 10.1007/s12665-023-10877-4 (2023).

[194] Lyra, A. & Loukas, A. Water and nitrogen use and agricultural production efficiency under climate change in a Mediterranean coastal watershed. *Environ. Sci. Proc.* **25** (1) (2023). 10.3390/ECWS-7-14180

[195] García-Ruiz, J. M., López-Moreno, J. I. & Vicente-Serrano, S. M. Mediterranean water resources in a global change scenario. *Earth Sci. Rev.* **105**, 3–4. 10.1016/j.earscirev.2011.01.006 (2011).

[196] Stigter, T. Y., Nunes, J. P. & Pisani, B. Comparative assessment of climate change and its impacts on three coastal aquifers in the mediterranean. *Reg. Envriron. Chang.* **14**, 41–56. 10.1007/s10113-012-0377-3 (2014).

[197] El Asri, H., Larabi, A. & Faouzi, M. Assessment of future climate trend based on multi-RCMs models and its impact on groundwater recharge of the Mediterranean coastal aquifer of Ghis-Nekkor (Morocco, in *Climate Change in the Mediterranean and Middle Eastern Region (Leal Filho*, M. W. and E., Eds., pp. 3–19. (2022). 10.1007/978-3-030-78566-6_1

[198] Pisinaras, V., Paraskevas, C. & Panagopoulos, A. Investigating the effects of agricultural water management in a mediterranean coastal aquifer under current and projected climate conditions. *Water* **13** (1). 10.3390/w13010108 (2021).

[199] Sherif, M., Sefelnasr, A. & Ebraheem, A. A. Quantitative and qualitative assessment of seawater intrusion in Wadi Ham under different pumping scenarios. *J. Hydrol. Eng.* **19** (5), 855–866. 10.1061/(ASCE)HE.1943-5584.000090 (2014).

[200] Owojori, O. J., Reinecke, A. J. & Rozanov, A. B. Effects of salinity on partitioning, uptake and toxicity of zinc in the earthworm Eisenia fetida. *Soil. Biol. Biochem. Spec. Sect. Enzymes Environ.* **40**, 2385–2393. 10.1016/j.soilbio.2008.05.019 (2008).

[201] Owojori, O. J., Waszak, K. & Roembke, J. Avoidance and reproduction tests with the predatory mite hypoaspis aculeifer: effects of different chemical substances. *Environ. Toxicol. Chem.* **33**, 230–237. 10.1002/etc.2421Pag (2014). 24122914 10.1002/etc.2421

[202] Bencherif, K., Boutekrabt, A. & Fontaine, J. Impact of soil salinity on arbuscular mycorrhizal fungi biodiversity and microflora biomass associated with Tamarix articulata Vahll rhizosphere in arid and semi-arid Algerian areas. *Sci. Total Environ.* **533**, 488–494. 10.1016/j.scitotenv.2015.07.007 (2015). 26184906 10.1016/j.scitotenv.2015.07.007

