# Supplementary material for: Climate change risks on key open marine and coastal mediterranean ecosystems
Source: Sci Rep. 2025 Jul 10;15:24907. doi: 10.1038/s41598-025-07858-x (PMC12246105; doi:10.1038/s41598-025-07858-x)
Supplement: Supplementary file 2 — Supplementary Material 2 [file 41598_2025_7858_MOESM2_ESM.docx]

**Supplementary Materials**

| **Ecosystems** | **Definition** |
| --- | --- |
| **Open marine ecosystems** | |
| Epipelagic | Epipelagic ecosystems refer to the uppermost part of the ocean with depth <200 m from the surface where there is enough sunlight to allow photosynthesis. |
| Coralligenous | The coralligenous is a typical Mediterranean underwater seascape, present on hard bottoms from ~15 to 120 m water depths, and is mainly produced by the accumulation of calcareous encrusting algae growing in dim light conditions and relatively calm waters. The coralligenous ecosystem fosters one of the richest assemblages found in the Mediterranean, most of which are long-lived algae and sessile invertebrates (sponges, corals, bryozoans and tunicates) [198], [199]. |
| Seagrass meadows | Seagrasses, marine flowering plants forming submerged meadows up to 40 m deep, constitute a major component of the Mediterranean marine ecosystems. The Mediterranean Sea is home to four seagrass genera (*Cymodocea, Halophila, Posidonia* and *Zostera*) encompassing four indigenous species (*C. nodosa, P. oceanica, Z. marina* and *Z. noltii*) and one non-indigenous species (*H. stipulacea*) [200]. |
| Fish | Fish is used as a collective term, including all types of native and invasive fish populations. As this category has a high socio-economic relevance, we chose to assess it independently, although it could be part of other ecosystems covered in our assessment. |
| Seaweeds | Seaweed includes types of *Rhodophyta* (red), *Phaeophyta* (brown) and *Chlorophyta* (green) macroalgae. Seaweeds are found over a wide depth range, from the surface to >100 m depth [201], [202]. Mediterranean seaweed ecosystems play several crucial roles (e.g., oxygen production, source of food and energy, and building and protective habitats). |
| Megafauna | Marine megafauna are broadly defined by their large size and important ecological functions. These include animals such as mammals, large fishes, and sea turtles [203], [204]. In the deep sea, where most organisms are small, some researchers extend "megafauna" to include benthic organisms visible in seafloor imagery (>1 cm to 10 cm). |
| **Coastal ecosystems** | |
| Sandy beaches and sand dunes | Sandy beaches and sand dunes ecosystems refer to all coastal Mediterranean sub- to supra-littoral sandy habitats. Risks were evaluated by considering their biological and physical aspects. |
| Rocky coasts | Rocky coasts ecosystems refer to rocky shores. Risks were evaluated by considering their biological and physical aspects. |
| Coastal wetlands | Coastal wetlands are dynamic ecosystems that exist at the interface between land and sea. Mediterranean coastal wetlands include a wide variety of natural habitats such as river deltas, coastal lagoons and salt marshes, intertidal wetlands, and coastal aquifers. Here, risks were evaluated based on articles tackling coastal wetlands in general without any distinction between the various habitats. |
| Coastal lagoons and deltas | Coastal lagoons and deltas are coastal wetlands. Coastal lagoons are shallow water bodies separated from the ocean by a barrier, connected to it at least temporarily by one or more restricted inlets. Deltas are coastal landforms composed of subaerial and subaqueous packages of fluvial-transported sediments that have formed an alluvial landscape by deposition at the mouth of a river. Here, risks were evaluated based on articles tackling specifically these habitats. |
| Salt marshes | Salt marshes are coastal wetlands that are flooded and drained by salt water brought in by the tides. Here, risks were evaluated based on articles tackling specifically these habitats. |
| Coastal aquifers | Coastal aquifers are groundwater systems that cross land-ocean boundaries. They provide freshwater and interact with coastal hazards and coastal ecosystems alike. |

**Supplementary material S1.** Specificities of the different marine and coastal ecosystems considered in the present study

**Supplementary material S2.** List of the publications assessed, the main drivers, the risks and the confidence level, ΔSST (°C) relative to the pre-industrial period and the corresponding projection scenarios, the study areas, the types of study, the country of the first authors. Modeling: no data generated and the study could be based on in situ data to feed models and neural networks; Lab. Experiment: Laboratory work; Mesocosm Experiment: Work that is conducted in natural environment with experiment manipulations; In situ: Data collected in the field; CO_2_ vents: Data/experiments produced/conducted in CO_2_ vents environments; Remote Sensing: Satellite and imagery Data; Review: Overview, systematic review, literature review, etc. In grey are highlighted the studies on which the risk assessment is based.


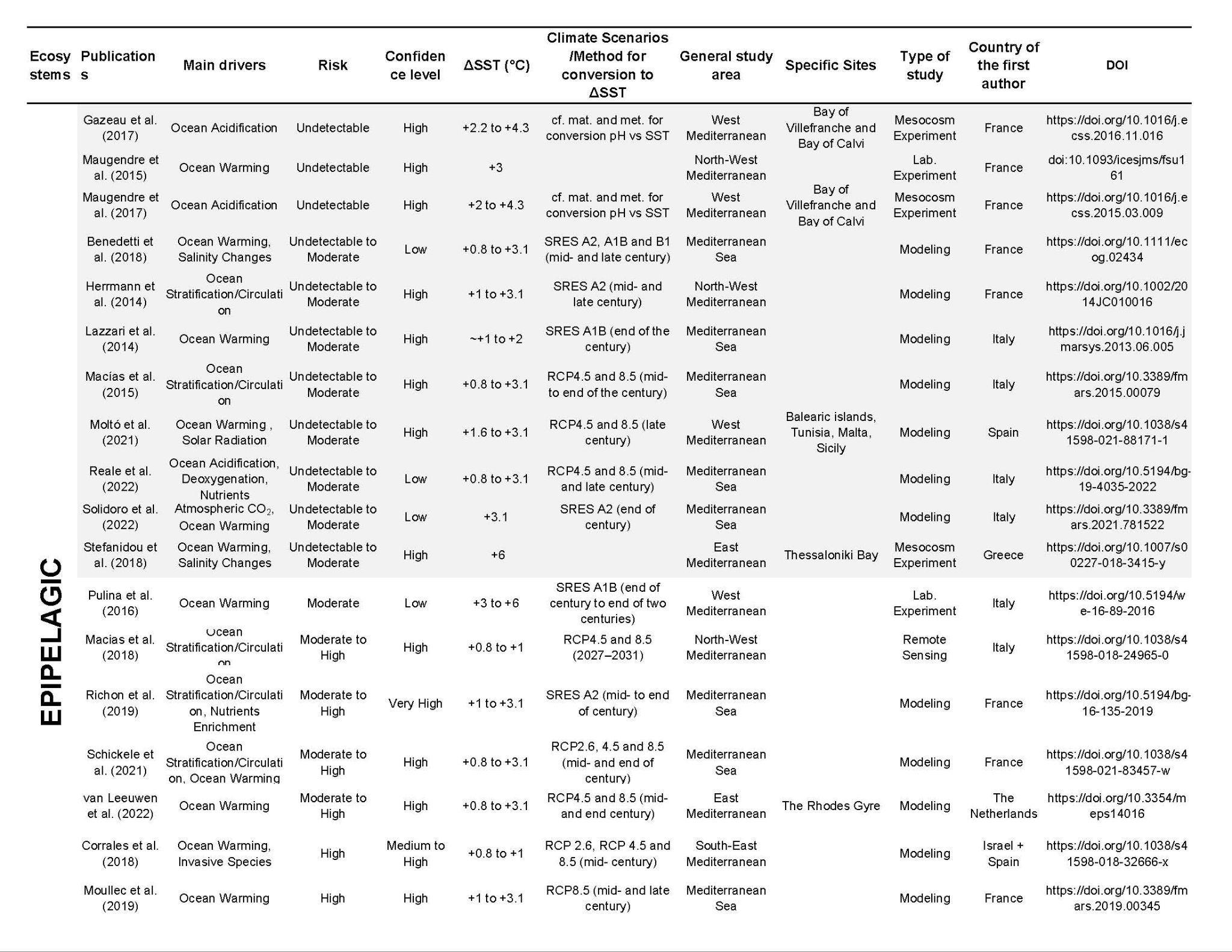

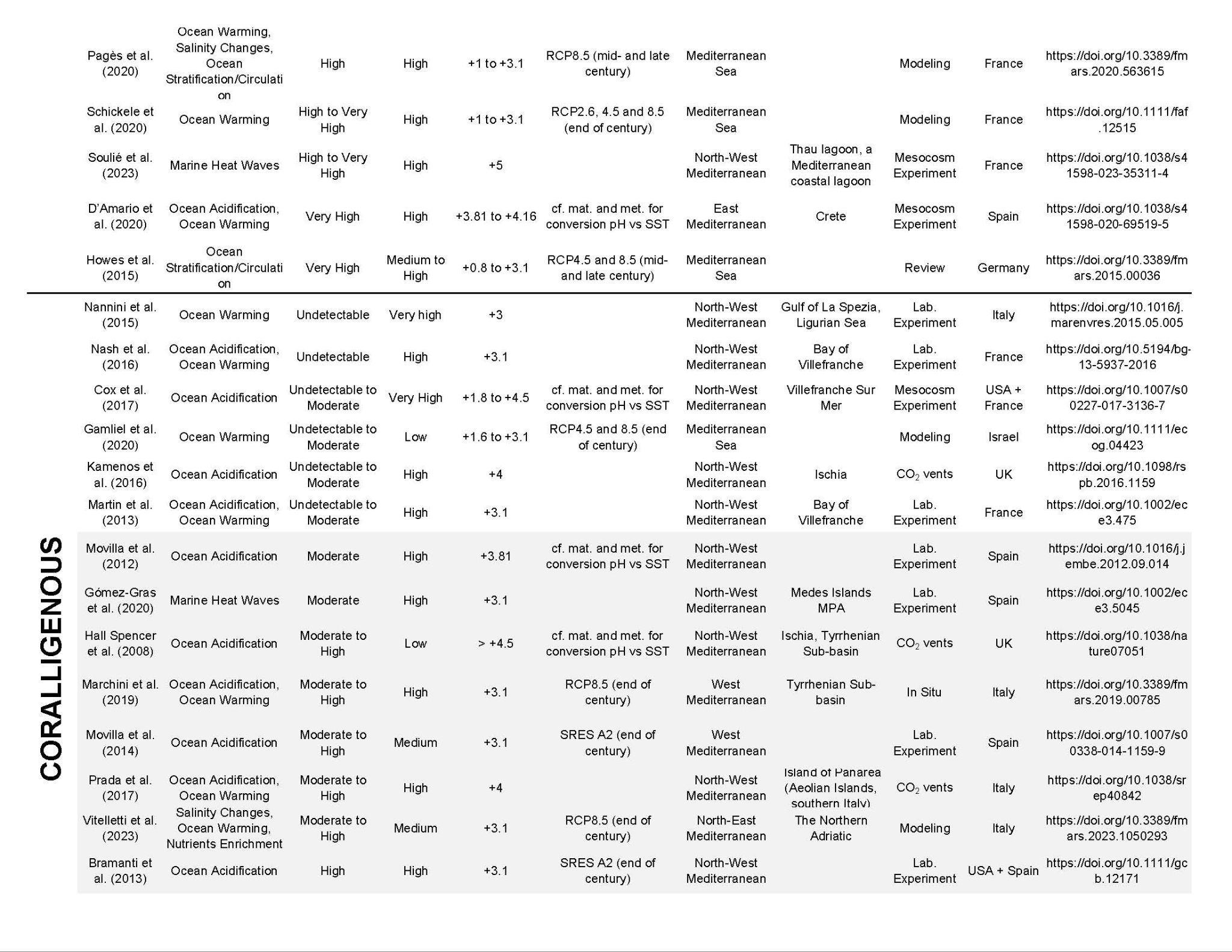

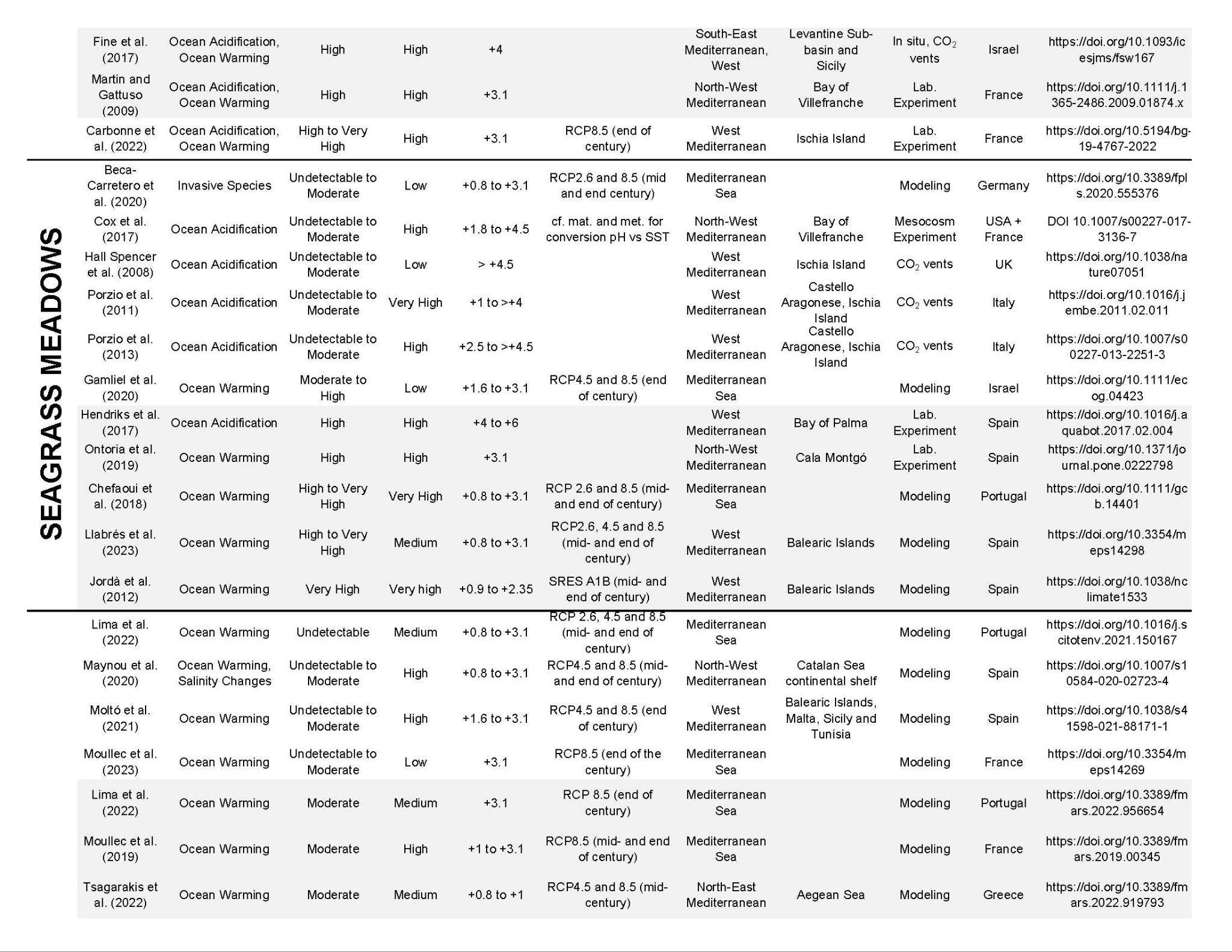

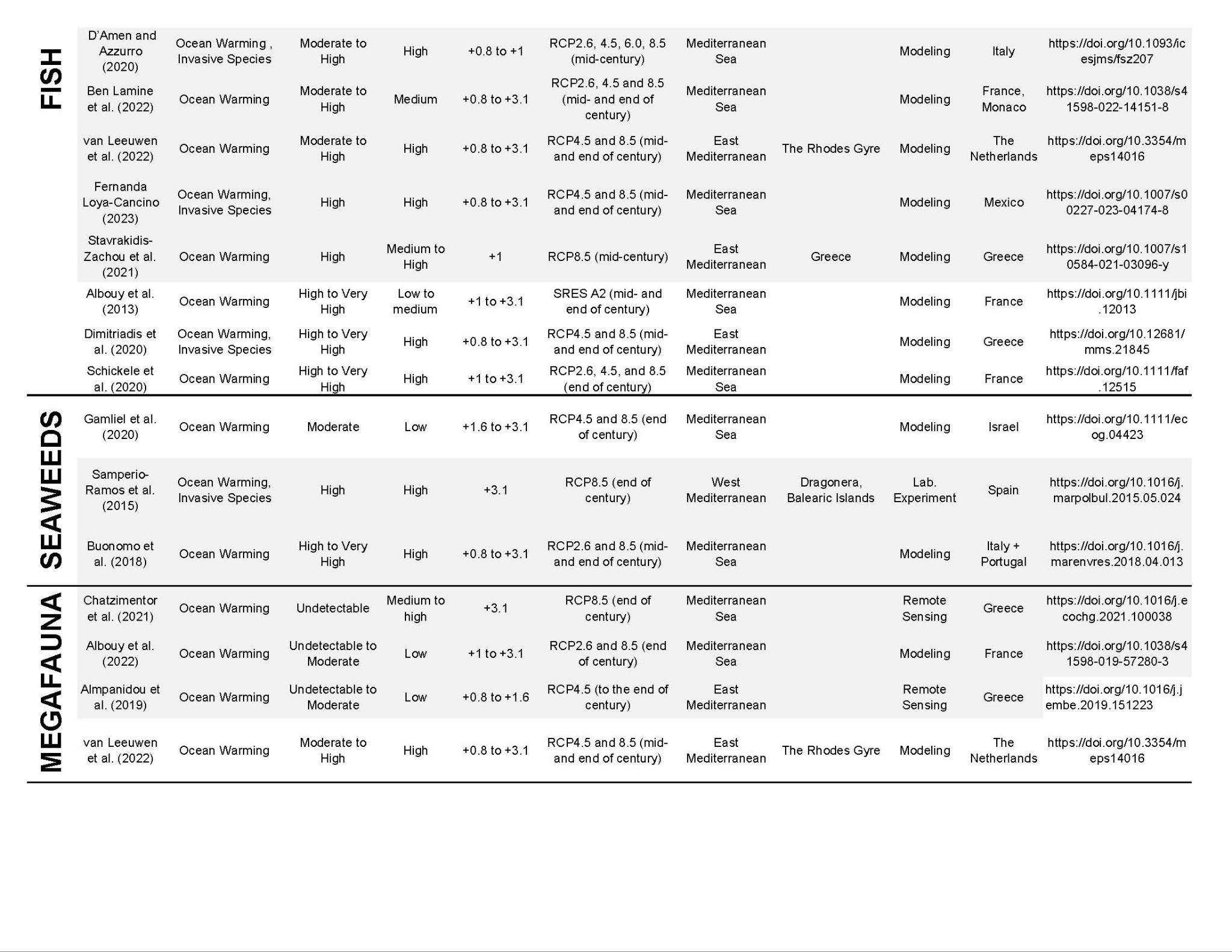


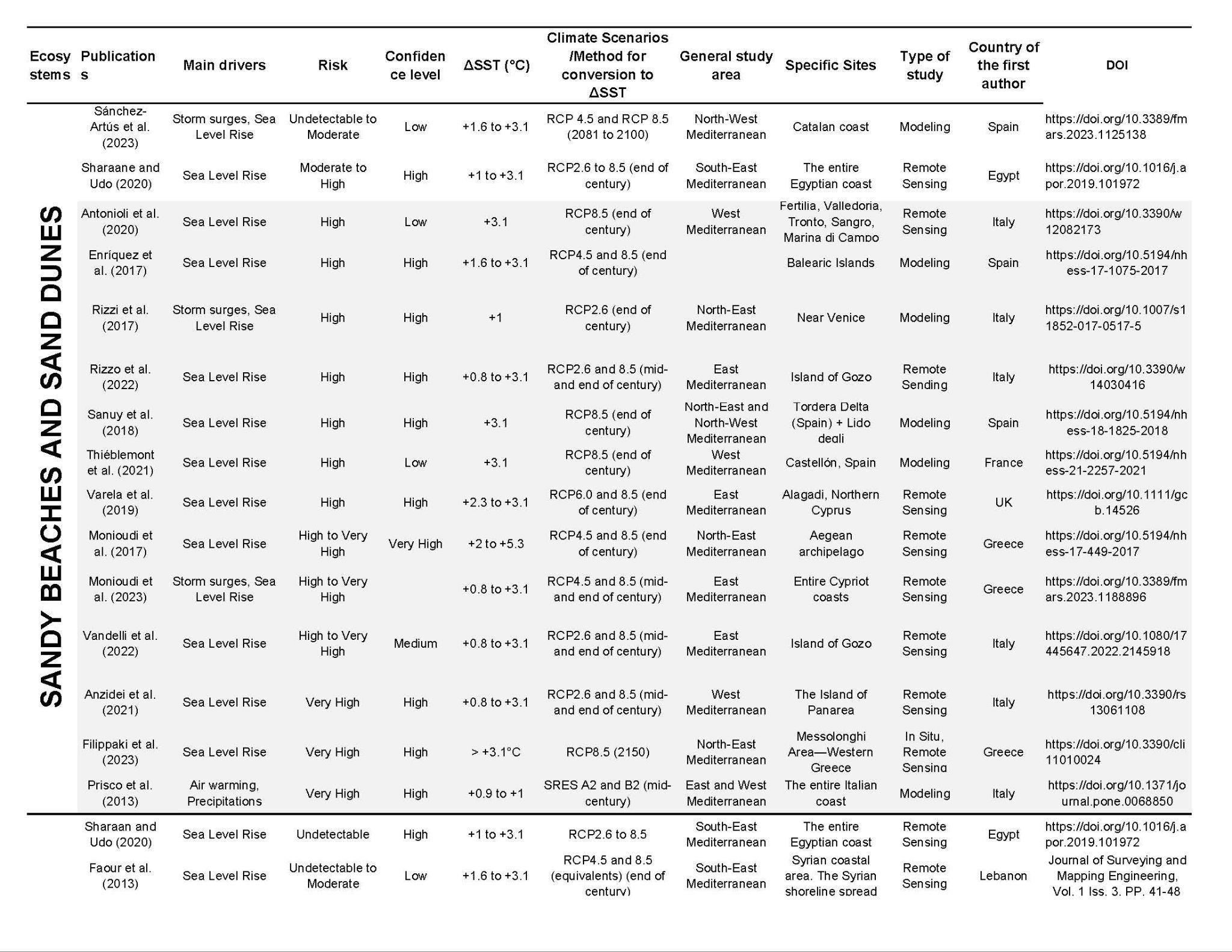

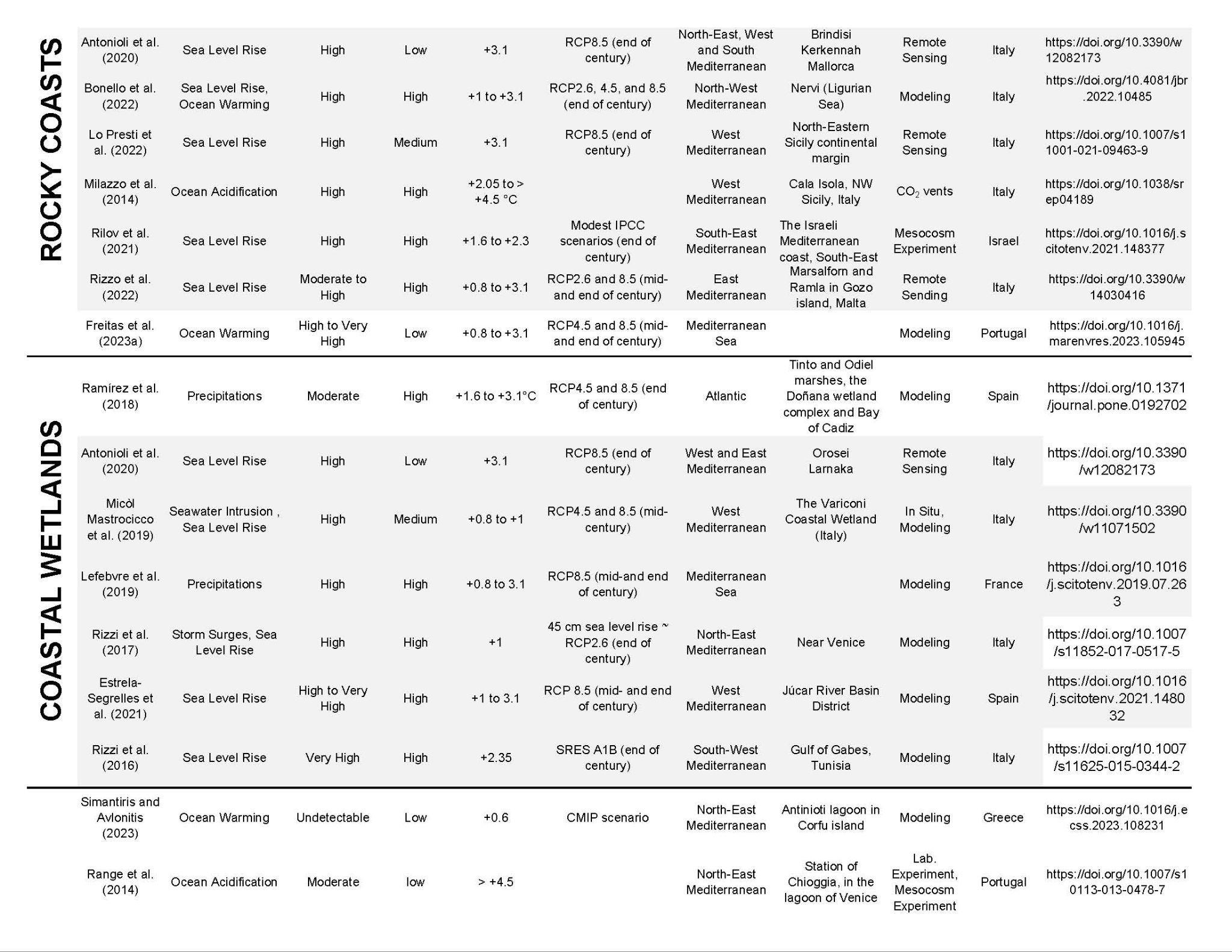

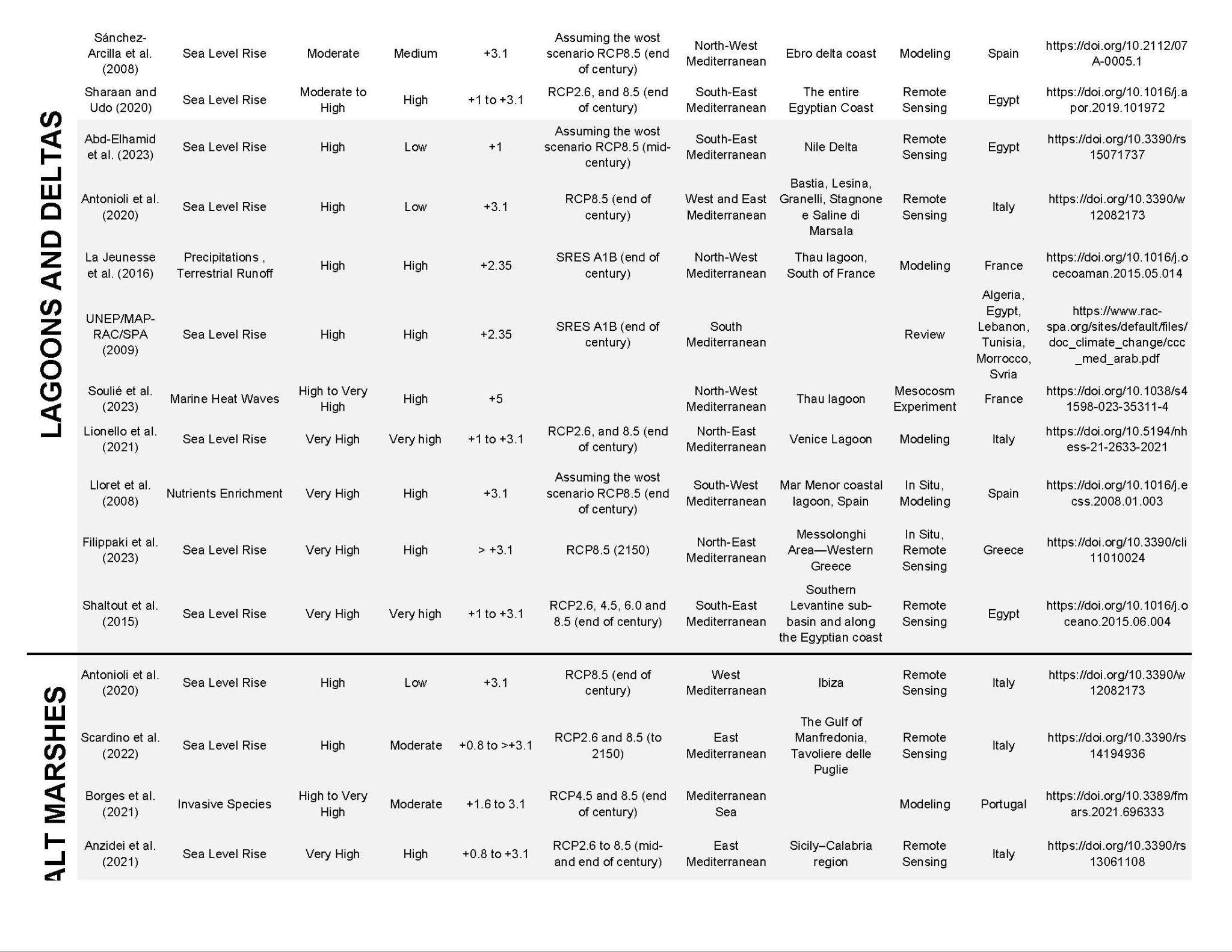

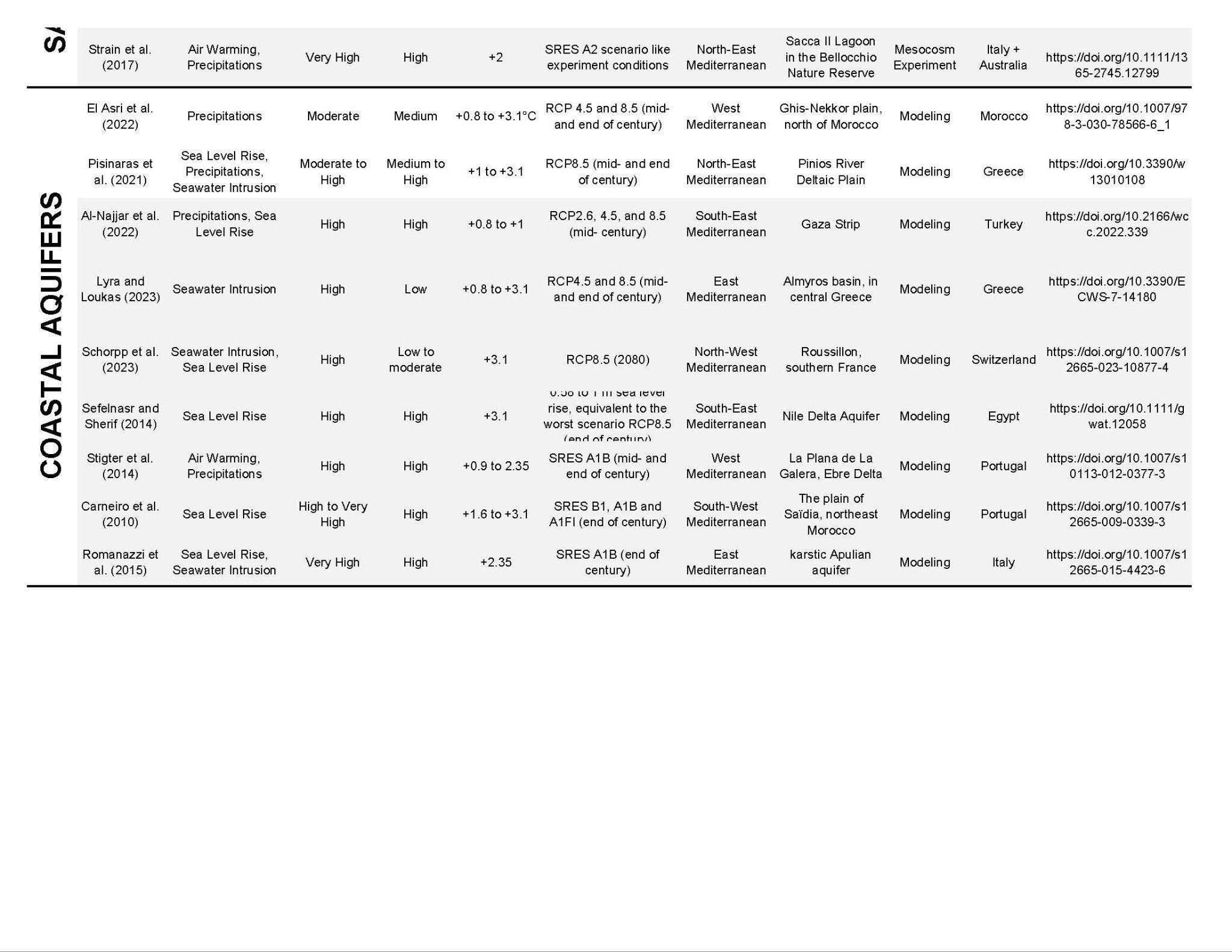


**Supplementary material S3.** Risk identification: Conversion approach and application to risk assessment

### Risk identification

We adopted the risk assessment approach used in multiple IPCC reports [205]. It distinguishes between four main risk levels (undetectable, moderate, high, very high) and three transition levels (undetectable-to-moderate, moderate-to-high, high-to-very high), which are all distributed along a color scale going from white when the risk is “undetectable” to purple when the risk is projected to be “very high”. The assessment considers the expected adaptive capacity of the ecosystems, their exposure and vulnerability. Impact and risk levels do not consider risk reduction strategies and/or future changes in non-climatic drivers. Risks for ecosystems were evaluated by considering biological, biogeochemical, geomorphological and physical aspects. Risks associated with composite effects of climate threats comprise habitat and biodiversity loss, changes in species composition and distribution ranges, and impacts/risks on ecosystem structure and functioning.

Our assessment of impacts and risks is based on changes in Mediterranean mean sea surface temperature (ΔSST) compared to the global mean surface temperature (∆GMST), as well as the corresponding changes in Mediterranean pH (ΔpH). In order to have the ∆GMST and ΔpH values that are equivalent to the Mediterranean ΔSST, the baseline of our risk assessment, we tried to have comparable reference periods. Therefore, we adjusted the ∆GMST and ΔpH values to obtain a reference period spanning from 1976 to 2020. The methodology can be summarized as follows:

**i)** Mediterranean ∆SST values were extracted from the study of [24] (multi-model values) relative to the reference period 1976–2005 (Table 1). These authors used numerical simulations to produce daily SST data between 1950–2005 for the historical experiment (HIST) and for 2006-2100 under various IPCC scenarios: the Representative Concentration Pathway RCP8.5 (high-emission scenario), RCP4.5 (moderate-emission scenario), and RCP2.6 (low-emission scenario). They defined 30-year periods from the HIST run between 1976–2005, the near future/mid-century (2021–2050) and the far future/end-of-century (2071–2100) which explains the range of time used for the calculated ∆SST.

**ii)** Anomalies of GMST (∆GMST) were first extracted from the IPCC (2019) relative to the period 1850-1900 and for two projected periods: near-term (2031-2050) and end-of-century (2081-2100) under the RCP8.5, RCP4.5 and RCP2.6 scenarios (Table 1). To convert the GMST anomalies from period 1850–1900 to period 2006–2015 (a reference period close to that of Mediterranean SST changes: 1976-2005, [24]), a 0.87ºC was subtracted as described in the Cross-Chapter Box 1 in the SPM of IPCC SR1.5 [7].

**iii)** Anomalies in pH for the Mediterranean Sea (∆pH) were extracted from the study of [39] for two projected periods: near-term (2050), and end-of-century (2100) under the Special Reports on Emission Scenarios (SRES) B1 and A1FI (Table 1). We used the values projected for the year 2020, in comparison to the pre-industrial period [39], as a reference to our ∆pH calculations, and we considered the average projected values between Eastern and Western Mediterranean Basins. To convert SRES temperature projections to RCP values, the equivalence table of [206] was used. Consequently, B1 was considered as RCP4.5 and A1FI as RCP8.5.

**Table 1.** Summary of the literature data used to construct the burning ember diagram. *Multi-model values; **Values calculated after subtracting 0.87°C from the ∆GMST (reference period 1850-1900); ***Average values between Western and Eastern Mediterranean Basins. Reference periods highlighted in grey are the ones considered for the burning ember.


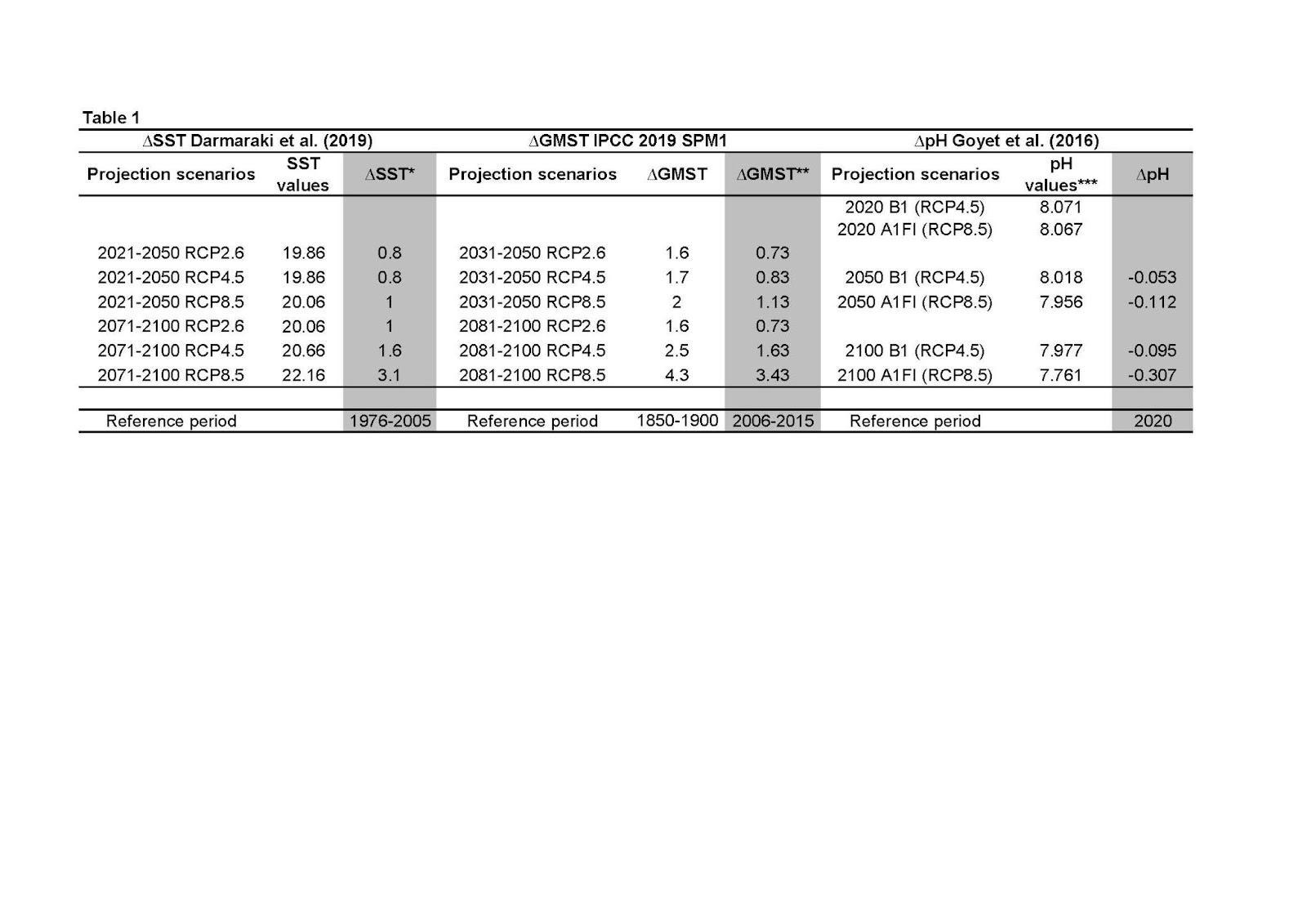


The outcomes in Table 1 (grey columns) were used to linearly plot ∆SST vs. ∆GMST and ∆SST vs. ∆pH in order to derive equations (1) and (2) that enabled us to convert ∆GMST and ∆pH into ∆SST in the studies where the latter was not used (plots are shown in the supplementary material):

***(S3.1)* ∆SST = (8.45 10-1 × ∆GMST) + 1.90 10-1**

***(S3.2)* ∆SST = (-8.80 × ∆pH) + 0.38**

where ∆SST is relative to the mean Mediterranean SST of 1976-2005, ∆GMST is relative to the mean GMST of the period 2006-2015 and ∆pH is relative to the Mediterranean pH in the year 2020 (projected in [39]).

Because the compiled studies usually presented different risks for a similar range of ∆SST, ∆GMST, or ∆pH, we took into consideration the majority of similar conclusions to assign risks while building the burning embers (n=86; Figure 2). In case of a tie, studies that were judged to be the most reliable (e.g., uncertainty assessment made, harmony with the assessments/conclusions of other studies, etc.) were used (see Supplementary material S3).

In order to identify risk levels in a logical and systematic way, we wanted to avoid having an abrupt change if the risk was assigned “moderate to high” or “high to very high”. To do so, we have applied a simple linear extrapolation, starting the risk transition with ∆SST=0°C as the minimum regional mean surface temperature change above the reference period, assuming that the risk is “undetectable”, unless it is mentioned differently at the low risk transition category. For example, when the minimum temperature was clearly stated for the middle risk transition “moderate to high”, we have divided the minimum temperature value to fill in the gaps in the lower risk transition category “undetectable to moderate” (these numbers are marked in red in the supplementary table to distinguish them from the ones clearly stated by the studies [in black]). The confidence level for the burning embers was assessed as explained in Supplementary material S3.

**Table 2.** Overview of the methodology applied for building the burning ember and assigning the confidence level.

| **Ecosystem** | **Risk extrapolated** | | **Risk based on the compiled studies** | | |
| --- | --- | --- | --- | --- | --- |
|  | **Risk** | **Confidence level** | **Risk** | **Justification** | **Confidence level** |
| Epipelagic |  |  | undetectable to moderate | based on 12 studies out of 23 because they present an agreement of results | medium confidence is assigned as nearly half of the compiled studies are projecting higher risks |
| Coralligenous | undetectable to moderate | low confidence due to the linear extrapolation applied | moderate to high | based on 10 studies out of 17 because it is the majority and they have concordant results | medium confidence is assigned as 7 out of the compiled 17 studies project other risks |
| Seagrass meadows | undetectable to high |  | high to very high | based on 5 studies out of 11 because they present concordant results | medium confidence is assigned as 6 out of the compiled 11 studies project other risks |
| Fish | undetectable to moderate |  | moderate to high | based on 8 studies out of 15 because they present concordant results | medium confidence is assigned as 7 out of the compiled 15 studies project other risks |
| Seaweeds | undetectable to high |  | high to very high | based on 2 studies out of 3 because they present concordant results | low confidence is assigned because of the very low number of studies |
| Megafauna |  |  | undetectable to moderate | based on 3 studies out of 4 | low confidence is assigned because of the limited number of studies |
| Sandy beaches and sand dunes | undetectable to high | low confidence due to the linear extrapolation applied | high to very high | based on 13 studies out of 15 | high confidence is assigned because the large majority of the studies agree on the risk |
| Rocky coasts | undetectable to moderate |  | moderate to high | based on 6 studies out of 9 | medium confidence is assigned because 6 out of 9 studies present high to very high risk - we selected studies stating moderate to high risk because they have higher confidence levels |
| Coastal wetlands | undetectable to high |  | high to very high | based on 6 studies out of 7 | high confidence is assigned because the large majority of the studies agree on the risk |
| Lagoons and deltas | undetectable to high |  | high to very high | based on 9 studies out of 13 | high confidence is assigned because the majority of the studies agree on the risk. |
| Salt marshes | undetectable to high |  | high to very high | based on 5 studies out of 5 | although all compiled studies agree on the risk, the limited number of studies explains the assigned medium confidence |
| Coastal aquifers | undetectable to high |  | high to very high | based on 7 studies out of 9 | medium confidence is assigned because 7 out of 9 studies present moderate to high risk - we selected studies stating high to very high risk because they have higher confidence levels |

**Supplementary material S4.** Risk levels and risk drivers with respect to the preindustrial values

This study is based on reference periods whose choice has been driven by the evidence of impacts in the scientific literature. The detailed methodology is described in S3. Specifically, the study adopts ∆SST_1976-2005_, Mediterranean Sea surface temperature difference with respect to the period 1976-2005, ∆GMST_2006-2015_, global mean surface temperature difference with respect to the period 2006-2015, and ∆pH_2000_ Mediterranean pH difference with respect to the year 2000. This section describes how the results can be transformed using the conventional 1850-2000 preindustrial period.

The maps of the IPCC Atlas [207], [208] allow to compute the ensemble mean sea surface temperature warming ΔSST_preindustrial_ with respect to the preindustrial values over the Mediterranean region (IPCC Atlas 2024) for fixed global warming levels (ΔGMST_preindustrial_ equal to 1.5°C, 2°C, 3°C, 4°C) and for different scenarios (SSP-2.6, 4.5, 7.0, 8.5) whose linear interpolation is

***(S4.1)*** ΔSST_Preindustrial_ = 0.868 ΔGMST_preindustrial_

Eqs. (S4.1) and (S3.1) represent analogue but not identical connections. The transformation of (S3.11) to preindustrial values can be carried out considering that GMST_2006-2015_ = GMST_preindustrial_ +0.87°C (according to observations (NOAA 2024) and consistently with IPCC reports and SST_1976-2005_ = SST_preindustrial_ +0.11°C (based on ERSST data [209], as compiled in [22]), which would transform (S4.1) to:

***(S4.2)*** ΔSST_Preindustrial_ = 0.845 ΔGMST_preindustrial_ -.43°C

Therefore, there is a substantial consistency on the rate of increase in (S4.1) and (S3.1) (the difference between the regression coefficients is lower than 3%), but (S3.1) has a cold bias. This bias is the consequence of a mismatch between the value SST warming in the period 1976-2005 according to climate model simulations (0.47°C warmer than preindustrial, as the 1976-2005 GMST is 0.54°C warmer than preindustrial) and the observed 1976-2005 SST of the Mediterranean. The difference is caused by multidecadal internal variability, namely by a strong cooling associated with a negative phase of AMOC [22]. The presence of this oscillation complicates the interpretation of the value of the SST driver in Figures 5 and 6. In the period 1976-2005 ΔGMST_preindustrial_=0.54°C, but the observed Mediterranean ΔSST_preindustrial_ corresponds to ΔGMST_preindustrial_=0.13°C. In the present condition (2014-2023) ΔGMST_preindustrial_ = 1.1°C, the observed ΔGMST_preindustrial_ = 1.17°C, which is 0.19°C warmer than the value provided by (S4.1), because of positive contribution of internal variability.

The IPCC Atlas allows also to compute the ensemble mean sea surface acidification ΔpH_preindustrial_ (by using the same procedure adopted for ΔSST_Preindustrial_) which results in linear relation:

***(S4.3)*** ΔpH_Preindustrial_ = -0.0917 ΔGMST_preindustrial_

On this basis (using ΔGMST_preindustrial_ =0.69°C) in 2000 would produce ΔpH_preindustrial_=-.063

and combining (S4.3) with (S4.1), one obtains:

***(S4.4)*** ΔSST_Preindustrial_= -9.43 ΔpH_preindustrial_ , - 0.48

In this case, the disagreement between (S4.3) and (S3.2) is important. The linear regression coefficient differs by about 7% between (S3.2) and (S4.4) and the latter implies an unrealistic acidity of 0.051 pH units of the Mediterranean Sea in the preindustrial period (when ΔSST_Preindustrial_ =0). A plausible explanation is that a linear relation does not reproduce correctly the evolution of ΔpH with global and SST warming. In fact, a quadratic expression improves appreciably, with respect to (S4.3), the values in the IPCC Atlas:

***(S4.5)*** ΔpH_Preindustrial_ =0.00775*ΔGMST^2^_preindustrial_- 0.1142 ΔGMST_preindustrial_

which suggests in year 2000 ΔpH_Preindustrial_ =-0.075, which is very close to the decrease of -0.8units estimated by [210] with respect to 1800.

A thumb rule for scaling the drivers of risks in Figures 5 and 6 would be:

● adding 0.87°C to ΔGMST.

● adding 0.47°C to ΔSST, being aware that the observed ΔSST can substantially deviate from the linear relation connecting it to ΔGMST, mostly because of the presence of oscillations with an amplitude of about 0.25°C and a period of 4 decades.

● subtracting 0.075 to ΔpH and considering that ΔpH values for large warming are likely overestimating the acidification of =.02 pH units.

**Supplementary material S5:** Mediterranean key habitats undergoing changes *vs* global ocean projections

Similar to global ocean projections [7], [211], all Mediterranean coastal ecosystems assessed in this study are projected to face higher risk levels than open marine ecosystems under all climate scenarios (Figure 6). However, climate-related stressors in the Mediterranean Sea seem to impact open marine and coastal ecosystems differently than in the global ocean (Figure S4).

Mediterranean epipelagic, coralligenous and rocky coasts ecosystems are projected to be more resilient (Figure S4). In the Mediterranean Sea, climate change seems to benefit primary production with a shift towards larger biomasses of small-sized groups [56], [57], [58], [62], [65], [66], [118] (medium confidence). In the global ocean, however, net primary production is projected to decline significantly especially in the tropics, under RCP8.5, and the sinking flux of organic matter from the upper ocean is projected to decrease (high confidence) [7]. These contrasting assessments may indicate a Mediterranean specificity due to its long evolutionary history, the presence of a variety of climatic and hydrological situations, and species belonging to several biogeographical categories. These differences may result in a higher adaptability of the epipelagic ecosystem to climate-related changes. There is also a possibility of an assessment bias since it is relying on the majority of studies (Table 3), although other studies showed higher projected risk resembling the ones predicted for the global ocean [62], [74], [75], [76]. Coralligenous habitats of the Mediterranean Sea and coral reefs in the tropical ocean are both home to a myriad of different species, and are both sensitive (or expected to be) mainly to ocean acidification and warming [212], [213], [214]. Several studies show that most coralligenous species in the Mediterranean will undergo moderate to high risk above ∆SST=+3.1°C, but are unlikely to completely disappear in a warming climate [84], [85], [87], [89], [90], [91]. Some coralligenous species are even expected to show no physiological [94] or mineralogical [95] changes, even under high *p*CO_2_. In the global ocean however, almost all warm-water coral reefs are already suffering and projected to suffer significant losses of area and local extinctions, even if global warming is limited to 1.5ºC (high confidence) and habitat-forming cold-water corals are expected to be significantly harmed through decreased calcification, increased dissolution of skeletons, and bioerosion (medium confidence) (IPCC, 2019).


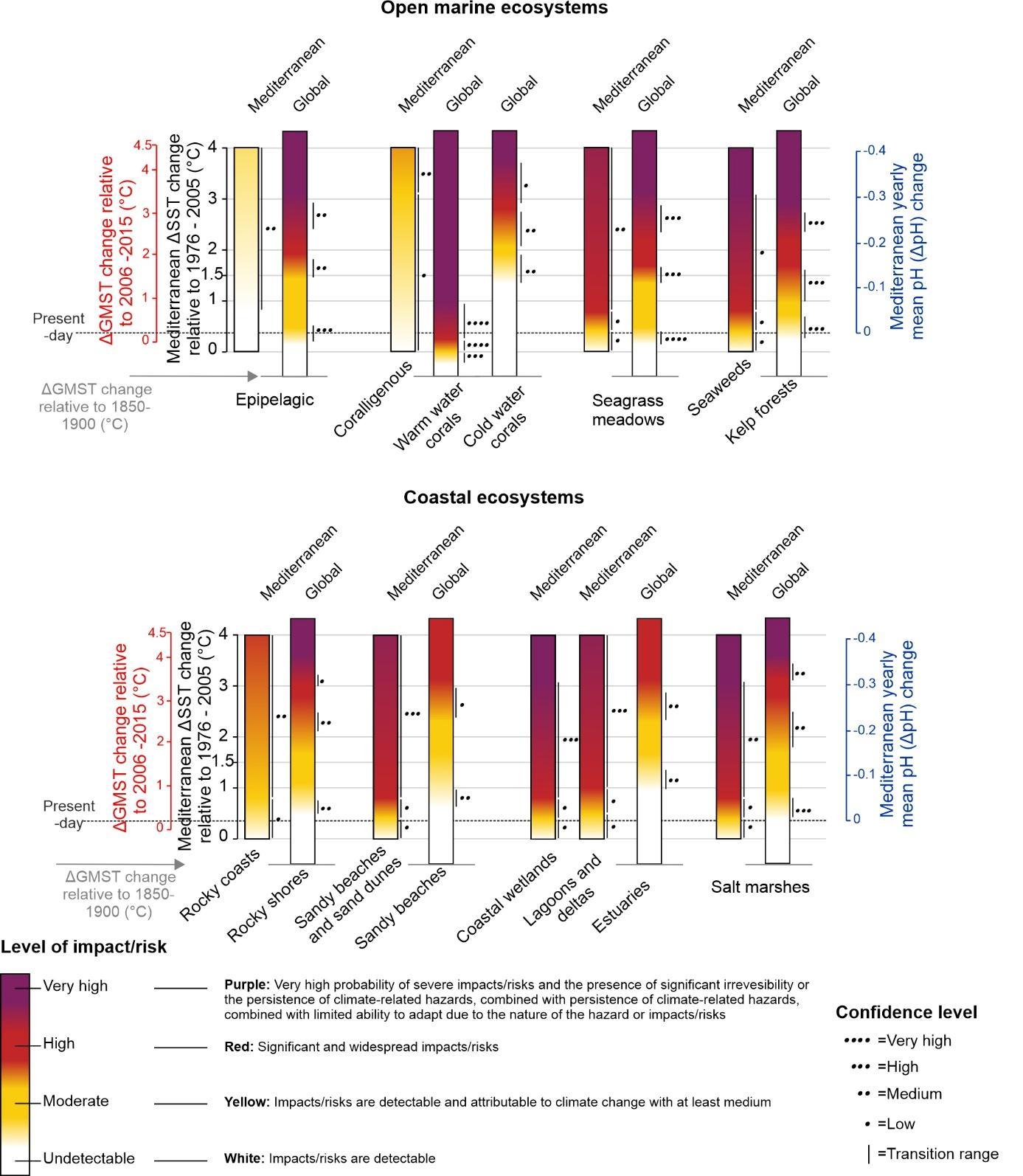


**Figure S5.** Comparison between projected risks for the global ocean ecosystems [7] and the Mediterranean Sea (this study).

Recent projections indicate negative effects on coral reefs (for RCP4.5 and RCP8.5), even under an optimistic scenario [214]. Our results show that Mediterranean coralligenous might be more resilient to climate change than previously expected. They also show heterogeneous effects of ocean acidification and ocean warming depending on the species assessed, suggesting the presence of potential winners and losers [92], [96]. These responses may reveal an ancestral plasticity within the group especially in species from highly fluctuating environments, thus making them potentially adaptable to global changes [97]. Climate-induced risks on Mediterranean rocky coasts are projected to be moderate to high (medium confidence) above ∆SST=+0.8°C (Figures 5; 6) [143], [155], [156], [157], [215]. In the global ocean, rocky shore ecosystems are projected to be at very high risk by 2100 under RCP8.5 (medium confidence) due to exposure to warming, as well as to acidification, SLR, and loss of calcifying species and biodiversity (high confidence) [7]. The differences in projections between the Mediterranean Sea and the global ocean might be due to the fact that most studies on which we based our assessment tackled the physical aspects of the rocky coasts [143], [156], [157], [215], while only few projected risks for the organisms inhabiting these ecosystems [155], [158]. If one only considers risks on the organisms of these habitats, the risk might be higher. This is attributed to projections in the Mediterranean showing that net primary production of intertidal reef communities will drastically drop under permanent submersion [155]. Some species will shift their habitats northwards [159], and others will be in danger of extinction within this century under low pH conditions [158].

Among Mediterranean marine ecosystems, seagrass meadows and seaweeds seem to be highly impacted at ΔGMST_(ref:2006-2015)_ warming of ~0.8°C (ΔGMST_(ref:1850-1900)_ ~1.6°C) (medium and low confidence, respectively) whereas globally, high risks are projected to be reached only when ΔGMST_(ref:2006-2015)_ exceeds ~1.3-1.5°C (ΔGMST_(ref:1850-1900)_ ~2.2-2.4°C) (high confidence) [7]; [211]. In the global ocean, above the aforementioned temperatures, these ecosystems will face altered structure (medium confidence). Following heat waves, a loss of seagrass meadows and seaweeds of 36–43% is projected in some areas (medium confidence), and disturbances in their growth rates and the occurrence of diseases are expected (e.g., [103], [106], [216], [217], [218]). Nonetheless, ocean acidification is expected to benefit photosynthesis and growth of seagrass meadows [219]. In the Mediterranean Sea, seagrass communities are predicted to have complex responses to ocean warming and acidification (see section III.1). The same applies for seaweeds, as high abundances of invasive species are forecasted in Mediterranean coastal areas [106], [127], similarly to what is projected for the global ocean where this can create more complex trophic interactions [220]. It is noteworthy that kelp forests were not well covered in projection studies conducted in the Mediterranean Sea, unlike the global ocean [7]. This might be attributed to the existence of poor kelp data, probably because the Mediterranean mostly hosts deep-water populations which are difficult to access [126].

The remarkably higher projected vulnerability of Mediterranean sandy beaches and sand dunes, coastal wetlands, lagoons and deltas and salt marshes to climate-related changes compared to the global ocean (Figure S4) could be attributed to the fact that the observed rates of climate change in the Mediterranean Basin exceed global trends for most variables that are majorly impacting these coastal ecosystems (SLR and warming; [10]) (Fig. 5). Despite the difference between the Mediterranean Sea and the global ocean in terms of temperature at which risks are expected to occur, the consequences on biodiversity and infrastructure might be comparable for these ecosystems (See section III.1 and [7]).
