# Supplementary material for: Climate change risks on key open marine and coastal mediterranean ecosystems
Source: Sci Rep. 2025 Jul 10;15:24907. doi: 10.1038/s41598-025-07858-x (PMC12246105; doi:10.1038/s41598-025-07858-x)
Supplement: Supplementary file 3 — Supplementary Material 3 [file 41598_2025_7858_MOESM3_ESM.pdf]

Supplementary Materials

Supplementary material S1. Specificities of the different marine and coastal ecosystems considered in the present study

Ecosystems	Definition
**Open marine ecosystems**	
Epipelagic	Epipelagic ecosystems refer to the uppermost part of the ocean with depth <200 m from the surface where there is enough sunlight to allow photosynthesis.
Coralligenous	The coralligenous is a typical Mediterranean underwater seascape, present on hard bottoms from ~15 to 120 m water depths, and is mainly produced by the accumulation of calcareous encrusting algae growing in dim light conditions and relatively calm waters. The coralligenous ecosystem fosters one of the richest assemblages found in the Mediterranean, most of which are long-lived algae and sessile invertebrates (sponges, corals, bryozoans and tunicates) [198], [199].
Seagrass meadows	Seagrasses, marine flowering plants forming submerged meadows up to 40 m deep, constitute a major component of the Mediterranean marine ecosystems. The Mediterranean Sea is home to four seagrass genera (*Cymodocea*, *Halophila*, *Posidonia* and *Zostera*) encompassing four indigenous species (*C. nodosa, P. oceanica, Z. marina* and *Z. noltii*) and one non-indigenous species (*H. stipulacea*) [200].
Fish	Fish is used as a collective term, including all types of native and invasive fish populations. As this category has a high socio-economic relevance, we chose to assess it independently, although it could be part of other ecosystems covered in our assessment.
Seaweeds	Seaweed includes types of *Rhodophyta* (red), *Phaeophyta* (brown) and *Chlorophyta* (green) macroalgae. Seaweeds are found over a wide depth range, from the surface to >100 m depth [201], [202]. Mediterranean seaweed ecosystems play several crucial roles (e.g., oxygen production, source of food and energy, and building and protective habitats).
Megafauna	Marine megafauna are broadly defined by their large size and important ecological functions. These include animals such as mammals, large fishes, and sea turtles [203], [204]. In the deep sea, where most organisms are small, some researchers extend "megafauna" to include benthic organisms visible in seafloor imagery (>1 cm to 10 cm).
**Coastal ecosystems**	
Sandy beaches and sand dunes	Sandy beaches and sand dunes ecosystems refer to all coastal Mediterranean sub- to supra-littoral sandy habitats. Risks were evaluated by considering their biological and physical aspects.
Rocky coasts	Rocky coasts ecosystems refer to rocky shores. Risks were evaluated by considering their biological and physical aspects.
Coastal wetlands	Coastal wetlands are dynamic ecosystems that exist at the interface between land and sea. Mediterranean coastal wetlands include a wide variety of natural habitats such as river deltas, coastal lagoons and salt marshes, intertidal wetlands, and coastal aquifers. Here, risks were evaluated based on articles tackling coastal wetlands in general without any distinction between the various habitats.
Coastal lagoons and deltas	Coastal lagoons and deltas are coastal wetlands. Coastal lagoons are shallow water bodies separated from the ocean by a barrier, connected to it at least temporarily by one or more restricted inlets. Deltas are coastal landforms composed of subaerial and subaqueous packages of fluvial-transported sediments that have formed an alluvial landscape by deposition at the mouth of a river. Here, risks were evaluated based on articles tackling specifically these habitats.
Salt marshes	Salt marshes are coastal wetlands that are flooded and drained by salt water brought in by the tides. Here, risks were evaluated based on articles tackling specifically these habitats.
Coastal aquifers	Coastal aquifers are groundwater systems that cross land-ocean boundaries. They provide freshwater and interact with coastal hazards and coastal ecosystems alike.

Supplementary material S2. List of the publications assessed, the main drivers, the risks and the confidence level, ΔSST (°C) relative to the pre-industrial period and the corresponding projection scenarios, the study areas, the types of study, the country of the first authors. Modeling: no data generated and the study could be based on in situ data to feed models and neural networks; Lab. Experiment: Laboratory work; Mesocosm Experiment: Work that is conducted in natural environment with experiment manipulations; In situ: Data collected in the field; CO_2 vents: Data/experiments produced/conducted in CO_2 vents environments; Remote Sensing: Satellite and imagery Data; Review: Overview, systematic review, literature review, etc. In grey are highlighted the studies on which the risk assessment is based.

Ecosystems	Publications	Main drivers	Risk	Confidence level	ΔSST (°C)	Climate Scenarios /Method for conversion to ΔSST	General study area	Specific Sites	Type of study	Country of the first author	DOI
EPIPELAGIC	Gazeau et al. (2017)	Ocean Acidification	Undetectable	High	+2.2 to +4.3	cf. mat. and met. for conversion pH vs SST	West Mediterranean	Bay of Villefranche and Bay of Calvi	Mesocosm Experiment	France	https://doi.org/10.1016/j.ecss.2016.11.016
	Maugendre et al. (2015)	Ocean Warming	Undetectable	High	+3		North-West Mediterranean		Lab. Experiment	France	doi:10.1093/icesjms/fsu161
	Maugendre et al. (2017)	Ocean Acidification	Undetectable	High	+2 to +4.3	cf. mat. and met. for conversion pH vs SST	West Mediterranean	Bay of Villefranche and Bay of Calvi	Mesocosm Experiment	France	https://doi.org/10.1016/j.ecss.2015.03.009
	Benedetti et al. (2018)	Ocean Warming, Salinity Changes	Undetectable to Moderate	Low	+0.8 to +3.1	SRES A2, A1B and B1 (mid- and late century)	Mediterranean Sea		Modeling	France	https://doi.org/10.1111/ecog.02434
	Herrmann et al. (2014)	Ocean Stratification/Circulation	Undetectable to Moderate	High	+1 to +3.1	SRES A2 (mid- and late century)	North-West Mediterranean		Modeling	France	https://doi.org/10.1002/2014JC010016
	Lazzari et al. (2014)	Ocean Warming	Undetectable to Moderate	High	~+1 to +2	SRES A1B (end of the century)	Mediterranean Sea		Modeling	Italy	https://doi.org/10.1016/j.jmarsys.2013.06.005
	Macías et al. (2015)	Ocean Stratification/Circulation	Undetectable to Moderate	High	+0.8 to +3.1	RCP4.5 and 8.5 (mid- to end of the century)	Mediterranean Sea		Modeling	Italy	https://doi.org/10.3389/fmars.2015.00079
	Moltó et al. (2021)	Ocean Warming , Solar Radiation	Undetectable to Moderate	High	+1.6 to +3.1	RCP4.5 and 8.5 (late century)	West Mediterranean	Balearic islands, Tunisia, Malta, Sicily	Modeling	Spain	https://doi.org/10.1038/s41598-021-88171-1
	Reale et al. (2022)	Ocean Acidification, Deoxygenation, Nutrients	Undetectable to Moderate	Low	+0.8 to +3.1	RCP4.5 and 8.5 (mid- and late century)	Mediterranean Sea		Modeling	Italy	https://doi.org/10.5194/bg-19-4035-2022
	Solidoro et al. (2022)	Atmospheric CO_2, Ocean Warming	Undetectable to Moderate	Low	+3.1	SRES A2 (end of century)	Mediterranean Sea		Modeling	Italy	https://doi.org/10.3389/fmars.2021.781522
	Stefanidou et al. (2018)	Ocean Warming, Salinity Changes	Undetectable to Moderate	High	+6		East Mediterranean	Thessaloniki Bay	Mesocosm Experiment	Greece	https://doi.org/10.1007/s00227-018-3415-y
	Pulina et al. (2016)	Ocean Warming	Moderate	Low	+3 to +6	SRES A1B (end of century to end of two centuries)	West Mediterranean		Lab. Experiment	Italy	https://doi.org/10.5194/we-16-89-2016
	Macias et al. (2018)	Ocean Stratification/Circulation	Moderate to High	High	+0.8 to +1	RCP4.5 and 8.5 (2027–2031)	North-West Mediterranean		Remote Sensing	Italy	https://doi.org/10.1038/s41598-018-24965-0
	Richon et al. (2019)	Ocean Stratification/Circulation, Nutrients Enrichment	Moderate to High	Very High	+1 to +3.1	SRES A2 (mid- to end of century)	Mediterranean Sea		Modeling	France	https://doi.org/10.5194/bg-16-135-2019
	Schickele et al. (2021)	Ocean Stratification/Circulation, Ocean Warming	Moderate to High	High	+0.8 to +3.1	RCP2.6, 4.5 and 8.5 (mid- and end of century)	Mediterranean Sea		Modeling	France	https://doi.org/10.1038/s41598-021-83457-w
	van Leeuwen et al. (2022)	Ocean Warming	Moderate to High	High	+0.8 to +3.1	RCP4.5 and 8.5 (mid- and end century)	East Mediterranean	The Rhodes Gyre	Modeling	The Netherlands	https://doi.org/10.3354/meps14016
	Corrales et al. (2018)	Ocean Warming, Invasive Species	High	Medium to High	+0.8 to +1	RCP 2.6, RCP 4.5 and 8.5 (mid- century)	South-East Mediterranean		Modeling	Israel + Spain	https://doi.org/10.1038/s41598-018-32666-x
	Moullec et al. (2019)	Ocean Warming	High	High	+1 to +3.1	RCP8.5 (mid- and late century)	Mediterranean Sea		Modeling	France	https://doi.org/10.3389/fmars.2019.00345

	Pagès et al. (2020)	Ocean Warming, Salinity Changes, Ocean Stratification/Circulation	High	High	+1 to +3.1	RCP8.5 (mid- and late century)	Mediterranean Sea		Modeling	France	https://doi.org/10.3389/fmars.2020.563615
	Schickele et al. (2020)	Ocean Warming	High to Very High	High	+1 to +3.1	RCP2.6, 4.5 and 8.5 (end of century)	Mediterranean Sea		Modeling	France	https://doi.org/10.1111/faf.12515
	Soulié et al. (2023)	Marine Heat Waves	High to Very High	High	+5		North-West Mediterranean	Thau lagoon, a Mediterranean coastal lagoon	Mesocosm Experiment	France	https://doi.org/10.1038/s41598-023-35311-4
	D'Amario et al. (2020)	Ocean Acidification, Ocean Warming	Very High	High	+3.81 to +4.16	cf. mat. and met. for conversion pH vs SST	East Mediterranean	Crete	Mesocosm Experiment	Spain	https://doi.org/10.1038/s41598-020-69519-5
	Howes et al. (2015)	Ocean Stratification/Circulation	Very High	Medium to High	+0.8 to +3.1	RCP4.5 and 8.5 (mid- and late century)	Mediterranean Sea		Review	Germany	https://doi.org/10.3389/fmars.2015.00036
CORALLIGENOUS	Nannini et al. (2015)	Ocean Warming	Undetectable	Very high	+3		North-West Mediterranean	Gulf of La Spezia, Ligurian Sea	Lab. Experiment	Italy	https://doi.org/10.1016/j.marenvres.2015.05.005
	Nash et al. (2016)	Ocean Acidification, Ocean Warming	Undetectable	High	+3.1		North-West Mediterranean	Bay of Villefranche	Lab. Experiment	France	https://doi.org/10.5194/bg-13-5937-2016
	Cox et al. (2017)	Ocean Acidification	Undetectable to Moderate	Very High	+1.8 to +4.5	cf. mat. and met. for conversion pH vs SST	North-West Mediterranean	Villefranche Sur Mer	Mesocosm Experiment	USA + France	https://doi.org/10.1007/s00227-017-3136-7
	Gamliel et al. (2020)	Ocean Warming	Undetectable to Moderate	Low	+1.6 to +3.1	RCP4.5 and 8.5 (end of century)	Mediterranean Sea		Modeling	Israel	https://doi.org/10.1111/ecog.04423
	Kamenos et al. (2016)	Ocean Acidification	Undetectable to Moderate	High	+4		North-West Mediterranean	Ischia	CO_2 vents	UK	https://doi.org/10.1098/rspb.2016.1159
	Martin et al. (2013)	Ocean Acidification, Ocean Warming	Undetectable to Moderate	High	+3.1		North-West Mediterranean	Bay of Villefranche	Lab. Experiment	France	https://doi.org/10.1002/ece3.475
	Movilla et al. (2012)	Ocean Acidification	Moderate	High	+3.81	cf. mat. and met. for conversion pH vs SST	North-West Mediterranean		Lab. Experiment	Spain	https://doi.org/10.1016/j.jembe.2012.09.014
	Gómez-Gras et al. (2020)	Marine Heat Waves	Moderate	High	+3.1		North-West Mediterranean	Medes Islands MPA	Lab. Experiment	Spain	https://doi.org/10.1002/ece3.5045
	Hall Spencer et al. (2008)	Ocean Acidification	Moderate to High	Low	> +4.5	cf. mat. and met. for conversion pH vs SST	North-West Mediterranean	Ischia, Tyrrhenian Sub-basin	CO_2 vents	UK	https://doi.org/10.1038/nature07051
	Marchini et al. (2019)	Ocean Acidification, Ocean Warming	Moderate to High	High	+3.1	RCP8.5 (end of century)	West Mediterranean	Tyrrhenian Sub-basin	In Situ	Italy	https://doi.org/10.3389/fmars.2019.00785
	Movilla et al. (2014)	Ocean Acidification	Moderate to High	Medium	+3.1	SRES A2 (end of century)	West Mediterranean		Lab. Experiment	Spain	https://doi.org/10.1007/s00338-014-1159-9
	Prada et al. (2017)	Ocean Acidification, Ocean Warming	Moderate to High	High	+4		North-West Mediterranean	Island of Panarea (Aeolian Islands, southern Italy)	CO_2 vents	Italy	https://doi.org/10.1038/srep40842
	Vitelletti et al. (2023)	Salinity Changes, Ocean Warming, Nutrients Enrichment	Moderate to High	Medium	+3.1	RCP8.5 (end of century)	North-East Mediterranean	The Northern Adriatic	Modeling	Italy	https://doi.org/10.3389/fmars.2023.1050293
	Bramanti et al. (2013)	Ocean Acidification	High	High	+3.1	SRES A2 (end of century)	North-West Mediterranean		Lab. Experiment	USA + Spain	https://doi.org/10.1111/gcb.12171

	Fine et al. (2017)	Ocean Acidification, Ocean Warming	High	High	+4		South-East Mediterranean, West	Levantine Sub-basin and Sicily	In situ, CO_2 vents	Israel	https://doi.org/10.1093/icesjms/fsw167
	Martin and Gattuso (2009)	Ocean Acidification, Ocean Warming	High	High	+3.1		North-West Mediterranean	Bay of Villefranche	Lab. Experiment	France	https://doi.org/10.1111/j.1365-2486.2009.01874.x
	Carbonne et al. (2022)	Ocean Acidification, Ocean Warming	High to Very High	High	+3.1	RCP8.5 (end of century)	West Mediterranean	Ischia Island	Lab. Experiment	France	https://doi.org/10.5194/bg-19-4767-2022
SEAGRASS MEADOWS	Beca-Carretero et al. (2020)	Invasive Species	Undetectable to Moderate	Low	+0.8 to +3.1	RCP2.6 and 8.5 (mid and end century)	Mediterranean Sea		Modeling	Germany	https://doi.org/10.3389/fpls.2020.555376
	Cox et al. (2017)	Ocean Acidification	Undetectable to Moderate	High	+1.8 to +4.5	cf. mat. and met. for conversion pH vs SST	North-West Mediterranean	Bay of Villefranche	Mesocosm Experiment	USA + France	DOI 10.1007/s00227-017-3136-7
	Hall Spencer et al. (2008)	Ocean Acidification	Undetectable to Moderate	Low	> +4.5		West Mediterranean	Ischia Island	CO_2 vents	UK	https://doi.org/10.1038/nature07051
	Porzio et al. (2011)	Ocean Acidification	Undetectable to Moderate	Very High	+1 to >+4		West Mediterranean	Castello Aragonese, Ischia Island	CO_2 vents	Italy	https://doi.org/10.1016/j.jembe.2011.02.011
	Porzio et al. (2013)	Ocean Acidification	Undetectable to Moderate	High	+2.5 to >+4.5		West Mediterranean	Castello Aragonese, Ischia Island	CO_2 vents	Italy	https://doi.org/10.1007/s00227-013-2251-3
	Gamliel et al. (2020)	Ocean Warming	Moderate to High	Low	+1.6 to +3.1	RCP4.5 and 8.5 (end of century)	Mediterranean Sea		Modeling	Israel	https://doi.org/10.1111/ecog.04423
	Hendriks et al. (2017)	Ocean Acidification	High	High	+4 to +6		West Mediterranean	Bay of Palma	Lab. Experiment	Spain	https://doi.org/10.1016/j.aquabot.2017.02.004
	Ontoria et al. (2019)	Ocean Warming	High	High	+3.1		North-West Mediterranean	Cala Montgó	Lab. Experiment	Spain	https://doi.org/10.1371/journal.pone.0222798
	Chefaoui et al. (2018)	Ocean Warming	High to Very High	Very High	+0.8 to +3.1	RCP 2.6 and 8.5 (mid- and end of century)	Mediterranean Sea		Modeling	Portugal	https://doi.org/10.1111/gcb.14401
	Llabrés et al. (2023)	Ocean Warming	High to Very High	Medium	+0.8 to +3.1	RCP2.6, 4.5 and 8.5 (mid- and end of century)	West Mediterranean	Balearic Islands	Modeling	Spain	https://doi.org/10.3354/meps14298
	Jordà et al. (2012)	Ocean Warming	Very High	Very high	+0.9 to +2.35	SRES A1B (mid- and end of century)	West Mediterranean	Balearic Islands	Modeling	Spain	https://doi.org/10.1038/nclimate1533
	Lima et al. (2022)	Ocean Warming	Undetectable	Medium	+0.8 to +3.1	RCP 2.6, 4.5 and 8.5 (mid- and end of century)	Mediterranean Sea		Modeling	Portugal	https://doi.org/10.1016/j.scitotenv.2021.150167
	Maynou et al. (2020)	Ocean Warming, Salinity Changes	Undetectable to Moderate	High	+0.8 to +3.1	RCP4.5 and 8.5 (mid- and end of century)	North-West Mediterranean	Catalan Sea continental shelf	Modeling	Spain	https://doi.org/10.1007/s10584-020-02723-4
	Moltó et al. (2021)	Ocean Warming	Undetectable to Moderate	High	+1.6 to +3.1	RCP4.5 and 8.5 (end of century)	West Mediterranean	Balearic Islands, Malta, Sicily and Tunisia	Modeling	Spain	https://doi.org/10.1038/s41598-021-88171-1
	Moullec et al. (2023)	Ocean Warming	Undetectable to Moderate	Low	+3.1	RCP8.5 (end of the century)	Mediterranean Sea		Modeling	France	https://doi.org/10.3354/meps14269
	Lima et al. (2022)	Ocean Warming	Moderate	Medium	+3.1	RCP 8.5 (end of century)	Mediterranean Sea		Modeling	Portugal	https://doi.org/10.3389/fmars.2022.956654
	Moullec et al. (2019)	Ocean Warming	Moderate	High	+1 to +3.1	RCP8.5 (mid- and end of century)	Mediterranean Sea		Modeling	France	https://doi.org/10.3389/fmars.2019.00345
	Tsagarakis et al. (2022)	Ocean Warming	Moderate	Medium	+0.8 to +1	RCP4.5 and 8.5 (mid-century)	North-East Mediterranean	Aegean Sea	Modeling	Greece	https://doi.org/10.3389/fmars.2022.919793

FISH	D'Amen and Azzurro (2020)	Ocean Warming , Invasive Species	Moderate to High	High	+0.8 to +1	RCP2.6, 4.5, 6.0, 8.5 (mid-century)	Mediterranean Sea		Modeling	Italy	https://doi.org/10.1093/icesjms/fsz207
	Ben Lamine et al. (2022)	Ocean Warming	Moderate to High	Medium	+0.8 to +3.1	RCP2.6, 4.5 and 8.5 (mid- and end of century)	Mediterranean Sea		Modeling	France, Monaco	https://doi.org/10.1038/s41598-022-14151-8
	van Leeuwen et al. (2022)	Ocean Warming	Moderate to High	High	+0.8 to +3.1	RCP4.5 and 8.5 (mid- and end of century)	East Mediterranean	The Rhodes Gyre	Modeling	The Netherlands	https://doi.org/10.3354/meps14016
	Fernanda Loya-Cancino (2023)	Ocean Warming, Invasive Species	High	High	+0.8 to +3.1	RCP4.5 and 8.5 (mid- and end of century)	Mediterranean Sea		Modeling	Mexico	https://doi.org/10.1007/s00227-023-04174-8
	Stavrakidis-Zachou et al. (2021)	Ocean Warming	High	Medium to High	+1	RCP8.5 (mid-century)	East Mediterranean	Greece	Modeling	Greece	https://doi.org/10.1007/s10584-021-03096-y
	Albouy et al. (2013)	Ocean Warming	High to Very High	Low to medium	+1 to +3.1	SRES A2 (mid- and end of century)	Mediterranean Sea		Modeling	France	https://doi.org/10.1111/jbi.12013
	Dimitriadis et al. (2020)	Ocean Warming, Invasive Species	High to Very High	High	+0.8 to +3.1	RCP4.5 and 8.5 (mid- and end of century)	East Mediterranean		Modeling	Greece	https://doi.org/10.12681/mms.21845
	Schickele et al. (2020)	Ocean Warming	High to Very High	High	+1 to +3.1	RCP2.6, 4.5, and 8.5 (end of century)	Mediterranean Sea		Modeling	France	https://doi.org/10.1111/faf.12515
SEAWEEDS	Gamliel et al. (2020)	Ocean Warming	Moderate	Low	+1.6 to +3.1	RCP4.5 and 8.5 (end of century)	Mediterranean Sea		Modeling	Israel	https://doi.org/10.1111/ecog.04423
	Samperio-Ramos et al. (2015)	Ocean Warming, Invasive Species	High	High	+3.1	RCP8.5 (end of century)	West Mediterranean	Dragonera, Balearic Islands	Lab. Experiment	Spain	https://doi.org/10.1016/j.marpolbul.2015.05.024
	Buonomo et al. (2018)	Ocean Warming	High to Very High	High	+0.8 to +3.1	RCP2.6 and 8.5 (mid- and end of century)	Mediterranean Sea		Modeling	Italy + Portugal	https://doi.org/10.1016/j.marenvres.2018.04.013
MEGAFAUNA	Chatzimentor et al. (2021)	Ocean Warming	Undetectable	Medium to high	+3.1	RCP8.5 (end of century)	Mediterranean Sea		Remote Sensing	Greece	https://doi.org/10.1016/j.ecochg.2021.100038
	Albouy et al. (2022)	Ocean Warming	Undetectable to Moderate	Low	+1 to +3.1	RCP2.6 and 8.5 (end of century)	Mediterranean Sea		Modeling	France	https://doi.org/10.1038/s41598-019-57280-3
	Almpanidou et al. (2019)	Ocean Warming	Undetectable to Moderate	Low	+0.8 to +1.6	RCP4.5 (to the end of century)	East Mediterranean		Remote Sensing	Greece	https://doi.org/10.1016/j.jembe.2019.151223
	van Leeuwen et al. (2022)	Ocean Warming	Moderate to High	High	+0.8 to +3.1	RCP4.5 and 8.5 (mid- and end of century)	East Mediterranean	The Rhodes Gyre	Modeling	The Netherlands	https://doi.org/10.3354/meps14016

Ecosystems	Publications	Main drivers	Risk	Confidence level	ΔSST (°C)	Climate Scenarios /Method for conversion to ΔSST	General study area	Specific Sites	Type of study	Country of the first author	DOI
SANDY BEACHES AND SAND DUNES	Sánchez-Artús et al. (2023)	Storm surges, Sea Level Rise	Undetectable to Moderate	Low	+1.6 to +3.1	RCP 4.5 and RCP 8.5 (2081 to 2100)	North-West Mediterranean	Catalan coast	Modeling	Spain	https://doi.org/10.3389/fmars.2023.1125138
	Sharaane and Udo (2020)	Sea Level Rise	Moderate to High	High	+1 to +3.1	RCP2.6 to 8.5 (end of century)	South-East Mediterranean	The entire Egyptian coast	Remote Sensing	Egypt	https://doi.org/10.1016/j.apor.2019.101972
	Antonioli et al. (2020)	Sea Level Rise	High	Low	+3.1	RCP8.5 (end of century)	West Mediterranean	Fertilia, Valledoria, Tronto, Sangro, Marina di Campo	Remote Sensing	Italy	https://doi.org/10.3390/w12082173
	Enríquez et al. (2017)	Sea Level Rise	High	High	+1.6 to +3.1	RCP4.5 and 8.5 (end of century)		Balearic Islands	Modeling	Spain	https://doi.org/10.5194/nhess-17-1075-2017
	Rizzi et al. (2017)	Storm surges, Sea Level Rise	High	High	+1	RCP2.6 (end of century)	North-East Mediterranean	Near Venice	Modeling	Italy	https://doi.org/10.1007/s11852-017-0517-5
	Rizzo et al. (2022)	Sea Level Rise	High	High	+0.8 to +3.1	RCP2.6 and 8.5 (mid- and end of century)	East Mediterranean	Island of Gozo	Remote Sending	Italy	https://doi.org/10.3390/w14030416
	Sanuy et al. (2018)	Sea Level Rise	High	High	+3.1	RCP8.5 (end of century)	North-East and North-West Mediterranean	Tordera Delta (Spain) + Lido degli	Modeling	Spain	https://doi.org/10.5194/nhess-18-1825-2018
	Thiéblemont et al. (2021)	Sea Level Rise	High	Low	+3.1	RCP8.5 (end of century)	West Mediterranean	Castellón, Spain	Modeling	France	https://doi.org/10.5194/nhess-21-2257-2021
	Varela et al. (2019)	Sea Level Rise	High	High	+2.3 to +3.1	RCP6.0 and 8.5 (end of century)	East Mediterranean	Alagadi, Northern Cyprus	Remote Sensing	UK	https://doi.org/10.1111/gcb.14526
	Monioudi et al. (2017)	Sea Level Rise	High to Very High	Very High	+2 to +5.3	RCP4.5 and 8.5 (end of century)	North-East Mediterranean	Aegean archipelago	Remote Sensing	Greece	https://doi.org/10.5194/nhess-17-449-2017
	Monioudi et al. (2023)	Storm surges, Sea Level Rise	High to Very High		+0.8 to +3.1	RCP4.5 and 8.5 (mid- and end of century)	East Mediterranean	Entire Cypriot coasts	Remote Sensing	Greece	https://doi.org/10.3389/fmars.2023.1188896
	Vandelli et al. (2022)	Sea Level Rise	High to Very High	Medium	+0.8 to +3.1	RCP2.6 and 8.5 (mid- and end of century)	East Mediterranean	Island of Gozo	Remote Sensing	Italy	https://doi.org/10.1080/17445647.2022.2145918
	Anzidei et al. (2021)	Sea Level Rise	Very High	High	+0.8 to +3.1	RCP2.6 and 8.5 (mid- and end of century)	West Mediterranean	The Island of Panarea	Remote Sensing	Italy	https://doi.org/10.3390/rs13061108
	Filippaki et al. (2023)	Sea Level Rise	Very High	High	> +3.1°C	RCP8.5 (2150)	North-East Mediterranean	Messolonghi Area—Western Greece	In Situ, Remote Sensing	Greece	https://doi.org/10.3390/cli11010024
	Prisco et al. (2013)	Air warming, Precipitations	Very High	High	+0.9 to +1	SRES A2 and B2 (mid-century)	East and West Mediterranean	The entire Italian coast	Modeling	Italy	https://doi.org/10.1371/journal.pone.0068850
	Sharaan and Udo (2020)	Sea Level Rise	Undetectable	High	+1 to +3.1	RCP2.6 to 8.5	South-East Mediterranean	The entire Egyptian coast	Remote Sensing	Egypt	https://doi.org/10.1016/j.apor.2019.101972
	Faour et al. (2013)	Sea Level Rise	Undetectable to Moderate	Low	+1.6 to +3.1	RCP4.5 and 8.5 (equivalents) (end of century)	South-East Mediterranean	Syrian coastal area. The Syrian shoreline spread	Remote Sensing	Lebanon	Journal of Surveying and Mapping Engineering, Vol. 1 Iss. 3, PP. 41-48

ROCKY COASTS	Antonioli et al. (2020)	Sea Level Rise	High	Low	+3.1	RCP8.5 (end of century)	North-East, West and South Mediterranean	Brindisi Kerkennah Mallorca	Remote Sensing	Italy	https://doi.org/10.3390/w 12082173
	Bonello et al. (2022)	Sea Level Rise, Ocean Warming	High	High	+1 to +3.1	RCP2.6, 4.5, and 8.5 (end of century)	North-West Mediterranean	Nervi (Ligurian Sea)	Modeling	Italy	https://doi.org/10.4081/jbr .2022.10485
	Lo Presti et al. (2022)	Sea Level Rise	High	Medium	+3.1	RCP8.5 (end of century)	West Mediterranean	North-Eastern Sicily continental margin	Remote Sensing	Italy	https://doi.org/10.1007/s1 1001-021-09463-9
	Milazzo et al. (2014)	Ocean Acidification	High	High	+2.05 to > +4.5 °C		West Mediterranean	Cala Isola, NW Sicily, Italy	CO_2 vents	Italy	https://doi.org/10.1038/sr ep04189
	Rilov et al. (2021)	Sea Level Rise	High	High	+1.6 to +2.3	Modest IPCC scenarios (end of century)	South-East Mediterranean	The Israeli Mediterranean coast, South-East	Mesocosm Experiment	Israel	https://doi.org/10.1016/j.s citotenv.2021.148377
	Rizzo et al. (2022)	Sea Level Rise	Moderate to High	High	+0.8 to +3.1	RCP2.6 and 8.5 (mid- and end of century)	East Mediterranean	Marsalforn and Ramla in Gozo island, Malta	Remote Sending	Italy	https://doi.org/10.3390/w 14030416
	Freitas et al. (2023a)	Ocean Warming	High to Very High	Low	+0.8 to +3.1	RCP4.5 and 8.5 (mid- and end of century)	Mediterranean Sea		Modeling	Portugal	https://doi.org/10.1016/j. marenvres.2023.105945
COASTAL WETLANDS	Ramírez et al. (2018)	Precipitations	Moderate	High	+1.6 to +3.1°C	RCP4.5 and 8.5 (end of century)	Atlantic	Tinto and Odiel marshes, the Doñana wetland complex and Bay of Cadiz	Modeling	Spain	https://doi.org/10.1371 /journal.pone.0192702
	Antonioli et al. (2020)	Sea Level Rise	High	Low	+3.1	RCP8.5 (end of century)	West and East Mediterranean	Orosei Larnaka	Remote Sensing	Italy	https://doi.org/10.3390 /w12082173
	Micòl Mastrocicco et al. (2019)	Seawater Intrusion , Sea Level Rise	High	Medium	+0.8 to +1	RCP4.5 and 8.5 (mid-century)	West Mediterranean	The Variconi Coastal Wetland (Italy)	In Situ, Modeling	Italy	https://doi.org/10.3390 /w11071502
	Lefebvre et al. (2019)	Precipitations	High	High	+0.8 to 3.1	RCP8.5 (mid-and end of century)	Mediterranean Sea		Modeling	France	https://doi.org/10.1016 /j.scitotenv.2019.07.26 3
	Rizzi et al. (2017)	Storm Surges, Sea Level Rise	High	High	+1	45 cm sea level rise ~ RCP2.6 (end of century)	North-East Mediterranean	Near Venice	Modeling	Italy	https://doi.org/10.1007 /s11852-017-0517-5
	Estrela-Segrelles et al. (2021)	Sea Level Rise	High to Very High	High	+1 to 3.1	RCP 8.5 (mid- and end of century)	West Mediterranean	Júcar River Basin District	Modeling	Spain	https://doi.org/10.1016 /j.scitotenv.2021.1480 32
	Rizzi et al. (2016)	Sea Level Rise	Very High	High	+2.35	SRES A1B (end of century)	South-West Mediterranean	Gulf of Gabes, Tunisia	Modeling	Italy	https://doi.org/10.1007 /s11625-015-0344-2
	Simantiris and Avlonitis (2023)	Ocean Warming	Undetectable	Low	+0.6	CMIP scenario	North-East Mediterranean	Antinioti lagoon in Corfu island	Modeling	Greece	https://doi.org/10.1016/j.e css.2023.108231
	Range et al. (2014)	Ocean Acidification	Moderate	low	> +4.5		North-East Mediterranean	Station of Chioggia, in the lagoon of Venice	Lab. Experiment, Mesocosm Experiment	Portugal	https://doi.org/10.1007/s1 0113-013-0478-7

	Sánchez-Arcilla et al. (2008)	Sea Level Rise	Moderate	Medium	+3.1	Assuming the wost scenario RCP8.5 (end of century)	North-West Mediterranean	Ebro delta coast	Modeling	Spain	https://doi.org/10.2112/07A-0005.1
	Sharaan and Udo (2020)	Sea Level Rise	Moderate to High	High	+1 to +3.1	RCP2.6, and 8.5 (end of century)	South-East Mediterranean	The entire Egyptian Coast	Remote Sensing	Egypt	https://doi.org/10.1016/j.apor.2019.101972
LAGOONS AND DELTAS	Abd-Elhamid et al. (2023)	Sea Level Rise	High	Low	+1	Assuming the wost scenario RCP8.5 (mid-century)	South-East Mediterranean	Nile Delta	Remote Sensing	Egypt	https://doi.org/10.3390/rs15071737
	Antonioli et al. (2020)	Sea Level Rise	High	Low	+3.1	RCP8.5 (end of century)	West and East Mediterranean	Bastia, Lesina, Granelli, Stagnone e Saline di Marsala	Remote Sensing	Italy	https://doi.org/10.3390/w12082173
	La Jeunesse et al. (2016)	Precipitations , Terrestrial Runoff	High	High	+2.35	SRES A1B (end of century)	North-West Mediterranean	Thau lagoon, South of France	Modeling	France	https://doi.org/10.1016/j.ocecoaman.2015.05.014
	UNEP/MAP-RAC/SPA (2009)	Sea Level Rise	High	High	+2.35	SRES A1B (end of century)	South Mediterranean		Review	Algeria, Egypt, Lebanon, Tunisia, Morrocco, Syria	https://www.rac-spa.org/sites/default/files/doc_climate_change/ccc_med_arab.pdf
	Soulié et al. (2023)	Marine Heat Waves	High to Very High	High	+5		North-West Mediterranean	Thau lagoon	Mesocosm Experiment	France	https://doi.org/10.1038/s41598-023-35311-4
	Lionello et al. (2021)	Sea Level Rise	Very High	Very high	+1 to +3.1	RCP2.6, and 8.5 (end of century)	North-East Mediterranean	Venice Lagoon	Modeling	Italy	https://doi.org/10.5194/nhess-21-2633-2021
	Lloret et al. (2008)	Nutrients Enrichment	Very High	High	+3.1	Assuming the wost scenario RCP8.5 (end of century)	South-West Mediterranean	Mar Menor coastal lagoon, Spain	In Situ, Modeling	Spain	https://doi.org/10.1016/j.ecss.2008.01.003
	Filippaki et al. (2023)	Sea Level Rise	Very High	High	> +3.1	RCP8.5 (2150)	North-East Mediterranean	Messolonghi Area—Western Greece	In Situ, Remote Sensing	Greece	https://doi.org/10.3390/cli11010024
	Shaltout et al. (2015)	Sea Level Rise	Very High	Very high	+1 to +3.1	RCP2.6, 4.5, 6.0 and 8.5 (end of century)	South-East Mediterranean	Southern Levantine sub-basin and along the Egyptian coast	Remote Sensing	Egypt	https://doi.org/10.1016/j.ocean.2015.06.004
ALT MARSHES	Antonioli et al. (2020)	Sea Level Rise	High	Low	+3.1	RCP8.5 (end of century)	West Mediterranean	Ibiza	Remote Sensing	Italy	https://doi.org/10.3390/w12082173
	Scardino et al. (2022)	Sea Level Rise	High	Moderate	+0.8 to >+3.1	RCP2.6 and 8.5 (to 2150)	East Mediterranean	The Gulf of Manfredonia, Tavoliere delle Puglie	Remote Sensing	Italy	https://doi.org/10.3390/rs14194936
	Borges et al. (2021)	Invasive Species	High to Very High	Moderate	+1.6 to 3.1	RCP4.5 and 8.5 (end of century)	Mediterranean Sea		Modeling	Portugal	https://doi.org/10.3389/fmars.2021.696333
	Anzidei et al. (2021)	Sea Level Rise	Very High	High	+0.8 to +3.1	RCP2.6 to 8.5 (mid- and end of century)	East Mediterranean	Sicily–Calabria region	Remote Sensing	Italy	https://doi.org/10.3390/rs13061108

SA	Strain et al. (2017)	Air Warming, Precipitations	Very High	High	+2	SRES A2 scenario like experiment conditions	North-East Mediterranean	Sacca II Lagoon in the Bellocchio Nature Reserve	Mesocosm Experiment	Italy + Australia	https://doi.org/10.1111/1365-2745.12799
COASTAL AQUIFERS	El Asri et al. (2022)	Precipitations	Moderate	Medium	+0.8 to +3.1°C	RCP 4.5 and 8.5 (mid- and end of century)	West Mediterranean	Ghis-Nekkor plain, north of Morocco	Modeling	Morocco	https://doi.org/10.1007/978-3-030-78566-6_1
	Pisinaras et al. (2021)	Sea Level Rise, Precipitations, Seawater Intrusion	Moderate to High	Medium to High	+1 to +3.1	RCP8.5 (mid- and end of century)	North-East Mediterranean	Pinios River Deltaic Plain	Modeling	Greece	https://doi.org/10.3390/w13010108
	Al-Najjar et al. (2022)	Precipitations, Sea Level Rise	High	High	+0.8 to +1	RCP2.6, 4.5, and 8.5 (mid- century)	South-East Mediterranean	Gaza Strip	Modeling	Turkey	https://doi.org/10.2166/wcc.2022.339
	Lyra and Loukas (2023)	Seawater Intrusion	High	Low	+0.8 to +3.1	RCP4.5 and 8.5 (mid- and end of century)	East Mediterranean	Almyros basin, in central Greece	Modeling	Greece	https://doi.org/10.3390/ECWS-7-14180
	Schorpp et al. (2023)	Seawater Intrusion, Sea Level Rise	High	Low to moderate	+3.1	RCP8.5 (2080)	North-West Mediterranean	Roussillon, southern France	Modeling	Switzerland	https://doi.org/10.1007/s12665-023-10877-4
	Sefelnasr and Sherif (2014)	Sea Level Rise	High	High	+3.1	0.58 to 1 m sea level rise, equivalent to the worst scenario RCP8.5 (end of century)	South-East Mediterranean	Nile Delta Aquifer	Modeling	Egypt	https://doi.org/10.1111/gwat.12058
	Stigter et al. (2014)	Air Warming, Precipitations	High	High	+0.9 to 2.35	SRES A1B (mid- and end of century)	West Mediterranean	La Plana de La Galera, Ebre Delta	Modeling	Portugal	https://doi.org/10.1007/s10113-012-0377-3
	Carneiro et al. (2010)	Sea Level Rise	High to Very High	High	+1.6 to +3.1	SRES B1, A1B and A1FI (end of century)	South-West Mediterranean	The plain of Saïdia, northeast Morocco	Modeling	Portugal	https://doi.org/10.1007/s12665-009-0339-3
	Romanazzi et al. (2015)	Sea Level Rise, Seawater Intrusion	Very High	High	+2.35	SRES A1B (end of century)	East Mediterranean	karstic Apulian aquifer	Modeling	Italy	https://doi.org/10.1007/s12665-015-4423-6

Supplementary material S3. Risk identification: Conversion approach and application to risk assessment

Risk identification

We adopted the risk assessment approach used in multiple IPCC reports [205]. It distinguishes between four main risk levels (undetectable, moderate, high, very high) and three transition levels (undetectable-to-moderate, moderate-to-high, high-to-very high), which are all distributed along a color scale going from white when the risk is "undetectable" to purple when the risk is projected to be "very high". The assessment considers the expected adaptive capacity of the ecosystems, their exposure and vulnerability. Impact and risk levels do not consider risk reduction strategies and/or future changes in non-climatic drivers. Risks for ecosystems were evaluated by considering biological, biogeochemical, geomorphological and physical aspects. Risks associated with composite effects of climate threats comprise habitat and biodiversity loss, changes in species composition and distribution ranges, and impacts/risks on ecosystem structure and functioning.

Our assessment of impacts and risks is based on changes in Mediterranean mean sea surface temperature (ΔSST) compared to the global mean surface temperature (ΔGMST), as well as the corresponding changes in Mediterranean pH (ΔpH). In order to have the ΔGMST and ΔpH values that are equivalent to the Mediterranean ΔSST, the baseline of our risk assessment, we tried to have comparable reference periods. Therefore, we adjusted the ΔGMST and ΔpH values to obtain a reference period spanning from 1976 to 2020. The methodology can be summarized as follows:

i) Mediterranean ΔSST values were extracted from the study of [24] (multi-model values) relative to the reference period 1976–2005 (Table 1). These authors used numerical simulations to produce daily SST data between 1950–2005 for the historical experiment (HIST) and for 2006-2100 under various IPCC scenarios: the Representative Concentration Pathway RCP8.5 (high-emission scenario), RCP4.5 (moderate-emission scenario), and RCP2.6 (low-emission scenario). They defined 30-year periods from the HIST run between 1976–2005, the near future/mid-century (2021–2050) and the far future/end-of-century (2071–2100) which explains the range of time used for the calculated ΔSST.

ii) Anomalies of GMST (ΔGMST) were first extracted from the IPCC (2019) relative to the period 1850-1900 and for two projected periods: near-term (2031-2050) and end-of-century (2081-2100) under the RCP8.5, RCP4.5 and RCP2.6 scenarios (Table 1). To convert the GMST anomalies from period 1850–1900 to period 2006–2015 (a reference period close to that of Mediterranean SST changes: 1976-2005, [24]), a 0.87ºC was subtracted as described in the Cross-Chapter Box 1 in the SPM of IPCC SR1.5 [7].

iii) Anomalies in pH for the Mediterranean Sea (ΔpH) were extracted from the study of [39] for two projected periods: near-term (2050), and end-of-century (2100) under the Special Reports on Emission Scenarios (SRES) B1 and A1FI (Table 1). We used the values projected for the year 2020, in comparison to the pre-industrial period [39], as a reference to our ΔpH calculations, and we considered the average projected values between Eastern and Western Mediterranean Basins. To convert SRES temperature projections to RCP values, the

equivalence table of [206] was used. Consequently, B1 was considered as RCP4.5 and A1FI as RCP8.5.

Table 1. Summary of the literature data used to construct the burning ember diagram. *Multi-model values; **Values calculated after subtracting 0.87°C from the ΔGMST (reference period 1850-1900); ***Average values between Western and Eastern Mediterranean Basins. Reference periods highlighted in grey are the ones considered for the burning ember.

ΔSST Darmaraki et al. (2019)			ΔGMST IPCC 2019 SPM1			ΔpH Goyet et al. (2016)		
Projection scenarios	SST values	ΔSST*	Projection scenarios	ΔGMST	ΔGMST**	Projection scenarios	pH values***	ΔpH
						2020 B1 (RCP4.5)	8.071	
						2020 A1FI (RCP8.5)	8.067	
2021-2050 RCP2.6	19.86	0.8	2031-2050 RCP2.6	1.6	0.73			
2021-2050 RCP4.5	19.86	0.8	2031-2050 RCP4.5	1.7	0.83	2050 B1 (RCP4.5)	8.018	-0.053
2021-2050 RCP8.5	20.06	1	2031-2050 RCP8.5	2	1.13	2050 A1FI (RCP8.5)	7.956	-0.112
2071-2100 RCP2.6	20.06	1	2081-2100 RCP2.6	1.6	0.73			
2071-2100 RCP4.5	20.66	1.6	2081-2100 RCP4.5	2.5	1.63	2100 B1 (RCP4.5)	7.977	-0.095
2071-2100 RCP8.5	22.16	3.1	2081-2100 RCP8.5	4.3	3.43	2100 A1FI (RCP8.5)	7.761	-0.307
Reference period		1976-2005	Reference period	1850-1900	2006-2015	Reference period		2020

The outcomes in Table 1 (grey columns) were used to linearly plot ΔSST vs. ΔGMST and ΔSST vs. ΔpH in order to derive equations (1) and (2) that enabled us to convert ΔGMST and ΔpH into ΔSST in the studies where the latter was not used (plots are shown in the supplementary material):

***(S3.1)* ΔSST = (8.45 10-1 × ΔGMST) + 1.90 10-1**

***(S3.2)* ΔSST = (-8.80 × ΔpH) + 0.38**

where ΔSST is relative to the mean Mediterranean SST of 1976-2005, ΔGMST is relative to the mean GMST of the period 2006-2015 and ΔpH is relative to the Mediterranean pH in the year 2020 (projected in [39]).

Because the compiled studies usually presented different risks for a similar range of ΔSST, ΔGMST, or ΔpH, we took into consideration the majority of similar conclusions to assign risks while building the burning embers (n=86; Figure 2). In case of a tie, studies that were judged to be the most reliable (e.g., uncertainty assessment made, harmony with the assessments/conclusions of other studies, etc.) were used (see Supplementary material S3).

In order to identify risk levels in a logical and systematic way, we wanted to avoid having an abrupt change if the risk was assigned “moderate to high” or “high to very high”. To do so, we have applied a simple linear extrapolation, starting the risk transition with ΔSST=0°C as the minimum regional mean surface temperature change above the reference period, assuming that the risk is “undetectable”, unless it is mentioned differently at the low risk transition category. For example, when the minimum temperature was clearly stated for the middle risk transition “moderate to high”, we have divided the minimum temperature value to fill in the gaps in the lower risk transition category “undetectable to moderate” (these numbers are marked in red in the supplementary table to distinguish them from the ones clearly stated by the studies [in

black]). The confidence level for the burning embers was assessed as explained in Supplementary material S3.

Table 2. Overview of the methodology applied for building the burning ember and assigning the confidence level.

**Ecosystem**	**Risk extrapolated**		**Risk based on the compiled studies**		
	**Risk**	**Confidence level**	**Risk**	**Justification**	**Confidence level**
Epipelagic			undetectable to moderate	based on 12 studies out of 23 because they present an agreement of results	medium confidence is assigned as nearly half of the compiled studies are projecting higher risks
Coralligenous	undetectable to moderate	low confidence due to the linear extrapolation applied	moderate to high	based on 10 studies out of 17 because it is the majority and they have concordant results	medium confidence is assigned as 7 out of the compiled 17 studies project other risks
Seagrass meadows	undetectable to high		high to very high	based on 5 studies out of 11 because they present concordant results	medium confidence is assigned as 6 out of the compiled 11 studies project other risks
Fish	undetectable to moderate		moderate to high	based on 8 studies out of 15 because they present concordant results	medium confidence is assigned as 7 out of the compiled 15 studies project other risks
Seaweeds	undetectable to high		high to very high	based on 2 studies out of 3 because they present concordant results	low confidence is assigned because of the very low number of studies
Megafauna			undetectable to moderate	based on 3 studies out of 4	low confidence is assigned because of the limited number of studies
Sandy beaches and sand dunes	undetectable to high	low confidence due to the linear extrapolation applied	high to very high	based on 13 studies out of 15	high confidence is assigned because the large majority of the studies agree on the risk
Rocky coasts	undetectable to moderate		moderate to high	based on 6 studies out of 9	medium confidence is assigned because 6 out of 9 studies present high to very

Ecosystem	Risk extrapolated		Risk based on the compiled studies		
	**Risk**	**Confidence level**	**Risk**	**Justification**	**Confidence level**
					high risk - we selected studies stating moderate to high risk because they have higher confidence levels
Coastal wetlands	undetectable to high		high to very high	based on 6 studies out of 7	high confidence is assigned because the large majority of the studies agree on the risk
Lagoons and deltas	undetectable to high		high to very high	based on 9 studies out of 13	high confidence is assigned because the majority of the studies agree on the risk.
Salt marshes	undetectable to high		high to very high	based on 5 studies out of 5	although all compiled studies agree on the risk, the limited number of studies explains the assigned medium confidence
Coastal aquifers	undetectable to high		high to very high	based on 7 studies out of 9	medium confidence is assigned because 7 out of 9 studies present moderate to high risk - we selected studies stating high to very high risk because they have higher confidence levels

Supplementary material S4. Risk levels and risk drivers with respect to the preindustrial values

This study is based on reference periods whose choice has been driven by the evidence of impacts in the scientific literature. The detailed methodology is described in S3. Specifically, the study adopts $\Delta SST_{1976\text{-}2005}$, Mediterranean Sea surface temperature difference with respect to the period 1976-2005, $\Delta GMST_{2006\text{-}2015}$, global mean surface temperature difference with respect to the period 2006-2015, and ΔpH_{2000} Mediterranean pH difference with respect to the year 2000. This section describes how the results can be transformed using the conventional 1850-2000 preindustrial period.

The maps of the IPCC Atlas [207], [208] allow to compute the ensemble mean sea surface temperature warming $\Delta SST_{preindustrial}$ with respect to the preindustrial values over the Mediterranean region (IPCC Atlas 2024) for fixed global warming levels ($\Delta GMST_{preindustrial}$ equal to 1.5°C, 2°C, 3°C, 4°C) and for different scenarios (SSP-2.6, 4.5, 7.0, 8.5) whose linear interpolation is

$$\textbf{\textit{(S4.1)}}\ \Delta SST_{Preindustrial} = 0.868\ \Delta GMST_{preindustrial}$$

Eqs. (S4.1) and (S3.1) represent analogue but not identical connections. The transformation of (S3.11) to preindustrial values can be carried out considering that $GMST_{2006\text{-}2015}$ = $GMST_{preindustrial}$ +0.87°C (according to observations (NOAA 2024) and consistently with IPCC reports and $SST_{1976\text{-}2005}$ = $SST_{preindustrial}$ +0.11°C (based on ERSST data [209], as compiled in [22]), which would transform (S4.1) to:

$$\textbf{\textit{(S4.2)}}\ \Delta SST_{Preindustrial} = 0.845\ \Delta GMST_{preindustrial}\ \text{-.43°C}$$

Therefore, there is a substantial consistency on the rate of increase in (S4.1) and (S3.1) (the difference between the regression coefficients is lower than 3%), but (S3.1) has a cold bias. This bias is the consequence of a mismatch between the value SST warming in the period 1976-2005 according to climate model simulations (0.47°C warmer than preindustrial, as the 1976-2005 GMST is 0.54°C warmer than preindustrial) and the observed 1976-2005 SST of the Mediterranean. The difference is caused by multidecadal internal variability, namely by a strong cooling associated with a negative phase of AMOC [22]. The presence of this oscillation complicates the interpretation of the value of the SST driver in Figures 5 and 6. In the period 1976-2005 $\Delta GMST_{preindustrial}$=0.54°C, but the observed Mediterranean $\Delta SST_{preindustrial}$ corresponds to $\Delta GMST_{preindustrial}$=0.13°C. In the present condition (2014-2023) $\Delta GMST_{preindustrial}$ = 1.1°C, the observed $\Delta GMST_{preindustrial}$ = 1.17°C, which is 0.19°C warmer than the value provided by (S4.1), because of positive contribution of internal variability.

The IPCC Atlas allows also to compute the ensemble mean sea surface acidification $\Delta pH_{preindustrial}$ (by using the same procedure adopted for $\Delta SST_{Preindustrial}$) which results in linear relation:

$$\textbf{\textit{(S4.3)}}\ \Delta pH_{Preindustrial} = -0.0917\ \Delta GMST_{preindustrial}$$

On this basis (using $\Delta GMST_{preindustrial}$ =0.69°C) in 2000 would produce $\Delta pH_{preindustrial}$=-.063 and combining (S4.3) with (S4.1), one obtains:

$$\textbf{\textit{(S4.4)}}\ \Delta SST_{Preindustrial} = -9.43\ \Delta pH_{preindustrial}\ ,\ -\ 0.48$$

In this case, the disagreement between (S4.3) and (S3.2) is important. The linear regression coefficient differs by about 7% between (S3.2) and (S4.4) and the latter implies an unrealistic acidity of 0.051 pH units of the Mediterranean Sea in the preindustrial period (when $\Delta SST_{Preindustrial}$ =0). A plausible explanation is that a linear relation does not reproduce correctly the evolution of ΔpH with global and SST warming. In fact, a quadratic expression improves appreciably, with respect to (S4.3), the values in the IPCC Atlas:

$$\textbf{\textit{(S4.5)}}\ \Delta pH_{Preindustrial} = 0.00775 * \Delta GMST^2_{preindustrial} - 0.1142\ \Delta GMST_{preindustrial}$$

which suggests in year 2000 $\Delta pH_{Preindustrial}$ =-0.075, which is very close to the decrease of -0.8units estimated by [210] with respect to 1800.

A thumb rule for scaling the drivers of risks in Figures 5 and 6 would be:

- adding 0.87°C to ΔGMST.
- adding 0.47°C to ΔSST, being aware that the observed ΔSST can substantially deviate from the linear relation connecting it to ΔGMST, mostly because of the presence of oscillations with an amplitude of about 0.25°C and a period of 4 decades.
- subtracting 0.075 to ΔpH and considering that ΔpH values for large warming are likely overestimating the acidification of =.02 pH units.

Supplementary material S5: Mediterranean key habitats undergoing changes *vs* global ocean projections

Similar to global ocean projections [7], [211], all Mediterranean coastal ecosystems assessed in this study are projected to face higher risk levels than open marine ecosystems under all climate scenarios (Figure 6). However, climate-related stressors in the Mediterranean Sea seem to impact open marine and coastal ecosystems differently than in the global ocean (Figure S4).

Mediterranean epipelagic, coralligenous and rocky coasts ecosystems are projected to be more resilient (Figure S4). In the Mediterranean Sea, climate change seems to benefit primary production with a shift towards larger biomasses of small-sized groups [56], [57], [58], [62], [65], [66], [118] (medium confidence). In the global ocean, however, net primary production is projected to decline significantly especially in the tropics, under RCP8.5, and the sinking flux of organic matter from the upper ocean is projected to decrease (high confidence) [7]. These contrasting assessments may indicate a Mediterranean specificity due to its long evolutionary history, the presence of a variety of climatic and hydrological situations, and species belonging to several biogeographical categories. These differences may result in a higher adaptability of the epipelagic ecosystem to climate-related changes. There is also a possibility of an assessment bias since it is relying on the majority of studies (Table 3), although other studies showed higher projected risk resembling the ones predicted for the global ocean [62], [74], [75], [76]. Coralligenous habitats of the Mediterranean Sea and coral reefs in the tropical ocean are both home to a myriad of different species, and are both sensitive (or expected to be) mainly to ocean acidification and warming [212], [213], [214]. Several studies show that most coralligenous species in the Mediterranean will undergo moderate to high risk above ΔSST=+3.1°C, but are unlikely to completely disappear in a warming climate [84], [85], [87], [89], [90], [91]. Some coralligenous species are even expected to show no physiological [94] or mineralogical [95] changes, even under high pCO_2. In the global ocean however, almost all warm-water coral reefs are already suffering and projected to suffer significant losses of area and local extinctions, even if global warming is limited to 1.5ºC (high confidence) and habitat-forming cold-water corals are expected to be significantly harmed through decreased calcification, increased dissolution of skeletons, and bioerosion (medium confidence) (IPCC, 2019).

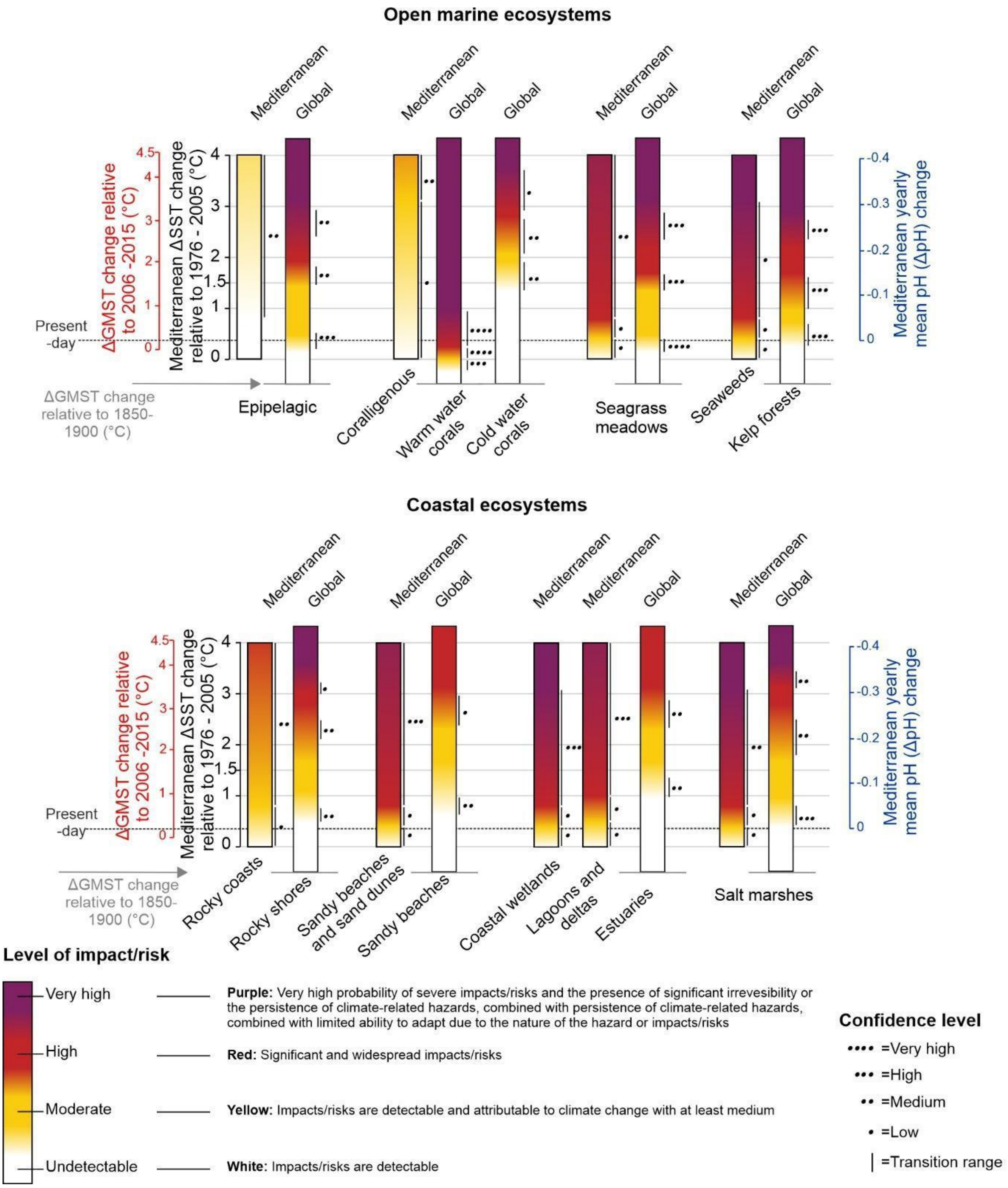

Figure S5. Comparison between projected risks for the global ocean ecosystems [7] and the Mediterranean Sea (this study).

Recent projections indicate negative effects on coral reefs (for RCP4.5 and RCP8.5), even under an optimistic scenario [214]. Our results show that Mediterranean coralligenous might be more resilient to climate change than previously expected. They also show heterogeneous effects of ocean acidification and ocean warming depending on the species assessed, suggesting the presence of potential winners and losers [92], [96]. These responses may reveal an ancestral plasticity within the group especially in species from highly fluctuating environments, thus making them potentially adaptable to global changes [97]. Climate-induced risks on Mediterranean rocky coasts are projected to be moderate to high (medium confidence) above

ΔSST=+0.8°C (Figures 5; 6) [143], [155], [156], [157], [215]. In the global ocean, rocky shore ecosystems are projected to be at very high risk by 2100 under RCP8.5 (medium confidence) due to exposure to warming, as well as to acidification, SLR, and loss of calcifying species and biodiversity (high confidence) [7]. The differences in projections between the Mediterranean Sea and the global ocean might be due to the fact that most studies on which we based our assessment tackled the physical aspects of the rocky coasts [143], [156], [157], [215], while only few projected risks for the organisms inhabiting these ecosystems [155], [158]. If one only considers risks on the organisms of these habitats, the risk might be higher. This is attributed to projections in the Mediterranean showing that net primary production of intertidal reef communities will drastically drop under permanent submersion [155]. Some species will shift their habitats northwards [159], and others will be in danger of extinction within this century under low pH conditions [158].

Among Mediterranean marine ecosystems, seagrass meadows and seaweeds seem to be highly impacted at $\Delta GMST_{(ref:2006-2015)}$ warming of ~0.8°C ($\Delta GMST_{(ref:1850-1900)}$ ~1.6°C) (medium and low confidence, respectively) whereas globally, high risks are projected to be reached only when $\Delta GMST_{(ref:2006-2015)}$ exceeds ~1.3-1.5°C ($\Delta GMST_{(ref:1850-1900)}$ ~2.2-2.4°C) (high confidence) [7]; [211]. In the global ocean, above the aforementioned temperatures, these ecosystems will face altered structure (medium confidence). Following heat waves, a loss of seagrass meadows and seaweeds of 36–43% is projected in some areas (medium confidence), and disturbances in their growth rates and the occurrence of diseases are expected (e.g., [103], [106], [216], [217], [218]). Nonetheless, ocean acidification is expected to benefit photosynthesis and growth of seagrass meadows [219]. In the Mediterranean Sea, seagrass communities are predicted to have complex responses to ocean warming and acidification (see section III.1). The same applies for seaweeds, as high abundances of invasive species are forecasted in Mediterranean coastal areas [106], [127], similarly to what is projected for the global ocean where this can create more complex trophic interactions [220]. It is noteworthy that kelp forests were not well covered in projection studies conducted in the Mediterranean Sea, unlike the global ocean [7]. This might be attributed to the existence of poor kelp data, probably because the Mediterranean mostly hosts deep-water populations which are difficult to access [126].

The remarkably higher projected vulnerability of Mediterranean sandy beaches and sand dunes, coastal wetlands, lagoons and deltas and salt marshes to climate-related changes compared to the global ocean (Figure S4) could be attributed to the fact that the observed rates of climate change in the Mediterranean Basin exceed global trends for most variables that are majorly impacting these coastal ecosystems (SLR and warming; [10]) (Fig. 5). Despite the difference between the Mediterranean Sea and the global ocean in terms of temperature at which risks are expected to occur, the consequences on biodiversity and infrastructure might be comparable for these ecosystems (See section III.1 and [7]).
